# Supplementary material for: Multi-ancestry GWAS of the electrocardiographic PR interval identifies 202 loci underlying cardiac conduction
Source: Nat Commun. 2020 May 21;11:2542. doi: 10.1038/s41467-020-15706-x (PMC7242331; doi:10.1038/s41467-020-15706-x)
Supplement: Supplementary file 1 — Supplementary Information [file 41467_2020_15706_MOESM1_ESM.pdf]

Supplementary Information

Multi-ancestry GWAS of the electrocardiographic PR interval identifies 202 loci underlying cardiac conduction.

Ntalla et al.

Contents

1. **Supplementary Methods**

(A) Description of participating studies

(B) Definition of previously reported and previously not reported loci

2. **Supplementary Tables**

Supplementary Table 1 Description of studies contributing to PR interval meta analyses: ancestry group, study design, sample size, and references.

Supplementary Table 2 Association results between polygenic risk score for PR interval and 16 select cardiac phenotypes.

3. **Supplementary Figures**

Supplementary Figure 1 Quantile-Quantile plots of autosomal variant results from meta-analyses of PR interval.

Supplementary Figure 2 Manhattan plot from the European (a), African (b), and Hispanic (c) meta-analyses of absolute PR interval.

Supplementary Figure 3 Region plots of 149 loci: 141 previously not reported loci from the multi-ancestry meta-analysis and 8 genome-wide significant loci in the European ancestry meta-analysis.

Supplementary Figure 4 Correlation of association statistics for the lead variants at the 141 previously not reported loci identified by the multi-ancestry meta-analysis and the 8 genome-wide significant loci in the European ancestry meta-analysis of absolute PR interval.

Supplementary Figure 5 Quantile-Quantile plots of chromosome X variant results from meta-analyses of absolute PR interval.

Supplementary Figure 6 Correlation of P-values across the meta-analyses of absolute and rank-based inverse normal transformed residuals of PR interval.

Supplementary Figure 7 Genome-wide significant loci ($P < 5 \times 10^{-8}$) across meta-analyses of absolute and rank-based inverse normal transformed residuals PR interval.

Supplementary Figure 8 Volcano plot of transcriptome-wide analysis for PR interval duration.

Supplementary Figure 9 Enrichment of PR interval variants in DNAse I Hypersensitive sites.

Supplementary Figure 10 Association of previously not reported PR interval loci with other GWAS traits.

Supplementary Figure 11 Associations between lead PR interval variants (205 single nucleotide polymorphisms) with atrial fibrillation (AF) risk from recently published AF GWASs

Supplementary Figure 12 Bubble plot of phenome-wide association analysis of multi-ancestry PR interval polygenic risk score.

4. Supplementary Notes

Supplementary Note 1: Acknowledgments

Supplementary Note 2: Funding

5. Supplementary References

1. **Supplementary Methods –**

   (A) Description of participating studies

Supplementary Table 1 indicates websites and references for further information on all contributing studies.

AMISH

The Old Order Amish (OOA) subjects included in this study were participants of several studies of cardiovascular health in relatively healthy volunteers from the OOA community of Lancaster County, PA and their family members. The studies were carried out at the University of Maryland as part of the Amish Complex Disease Research Program (ACDRP). The OOA population of Lancaster County, PA immigrated to the Colonies from Western Europe in the early 1700s. All study protocols were approved by the institutional review board at the University of Maryland and participating institutions. Informed consent was obtained from each of the study participants.

ARIC

The Atherosclerosis Risk in Communities (ARIC) Study[1] is a prospective community-based study of cardiovascular disease and its risk factors. At baseline (1987-89), 15,792 men and women age 45-64 were recruited from 4 communities in the US (Washington County, Maryland; Forsyth County, North Carolina; Jackson, Mississippi; Minneapolis suburbs, Minnesota). Participants were mostly white in the Minnesota and Washington County field centers, white and African American in Forsyth County, and exclusively African American in

the Jackson field center. ECGs were recorded on MAC PC Personal Cardiographs (Marquette Electronics Inc., Milwaukee, WI) and were subsequently submitted to a central reading center at the EPICORE Center (University of Alberta, Edmonton, Alberta, Canada) and thereafter to the Epidemiological Cardiology Research Center (EPICARE), Wake Forest University, Winston-Salem, NC. All ECGs were visually inspected for quality and legibility at their acquisition and by the reading centers and then stored in a digital format. The PR interval was determined as the mean duration in milliseconds from the P wave onset until the initiation of the QRS segment in the 12 ECG leads.

BAMBUÍ

A cohort study designed to identify predictors of adverse health events in the elderly. The study population comprises all residents of Bambuí (Minas Gerais, Brazil), aged 60 or more years (N=1,742). From these, 92.2% were interviewed and 85.9% underwent clinical examination, consisting of haematological and biochemical tests, serology for Trypanosoma cruzi, anthropometric and blood pressure measures and electrocardiogram. Cohort members undergo annual follow-up visits, which consist of an interview and verification of death certificates. Other procedures were repeated in selected years (2000, 2002 and 2008). From 1997 to 2007, during a mean follow-up of 8.6 years, 641 participants died and 96 (6.0%) were lost to follow-up.

BioMe

The Mount Sinai BioMe Biobank, founded in September 2007, is an ongoing, broadly consented EHR-linked bio- and data repository that enrolls participants non-selectively from

the Mount Sinai Medical Center patient population. The BioMe Biobank draws from a population of over 70,000 inpatient and 800,000 outpatient visits annually from over 30 broadly selected clinical sites of the Mount Sinai Medical Center (MSMC). As of September 2017, BioMe has enrolled more than 42,000 patients that represent a broad racial, ethnic and socioeconomic diversity with a distinct and population-specific disease burden, characteristic of the communities served by Mount Sinai Hospital. BioMe participants are predominantly of African (AA, 24%), Hispanic/Latino (HL, 35%), European (EA, 32%), and other ancestry (OA, 10%). The BioMe Biobank Program operates under a Mount Sinai Institutional Review Board-approved research protocol. All study participants provided written informed consent.

BRIGHT

Participants of the BRIGHT Study were recruited from the Medical Research Council General Practice Framework and other primary care practices in the UK. Each case had a history of hypertension diagnosed prior to 60 years of age with confirmed blood pressure recordings corresponding to seated levels >150/100mmHg (1 reading) or mean of 3 readings >145/95 mmHg. The BRIGHT study focused on the recruitment of hypertensive individuals with BMI < 30. Sample selection for GWAS was based on DNA availability and quantity.

Broad-AF

The Broad AF Study is a collaborative project to investigate the genetic determinants of atrial fibrillation (AF), comprised of 17,517 AF cases and 10,987 referents from 26 studies. Details of study description, genotyping, and imputation were described previously. [2] Briefly, genetic variants were centrally genotyped on the Infinium PsychArray-24 v1.2 Bead Chip, and jointly

called and quality controlled at the Broad Institute. After pre-imputation quality control, variants were imputed using the 1000 Genomes reference panel. A total of 3,461 individuals free of AF met the inclusion criteria of the current study and were included in the PR analysis. Individuals from the following participating studies were included: Vanderbilt University Medical Center Biobank (BioVU), Australian Familial AF Study, Danish AF Study, Groningen Genetics of Atrial Fibrillation (GGAF), Genetic Risk Assessment of Defibrillator Events Study (GRADE), Malmö Preventive Project (MPP-AF, and MPP-Echo), and Intermountain INSPIRE Registry. Study details were previously reported. [2]

CAMP

The MGH Cardiology and Metabolic Patient Cohort is comprised of 3850 subjects recruited from the ambulatory MGH Cardiology Practice between 2009 and 2012.

CHRIS

The Cooperative Health Research In South Tyrol (CHRIS) study is a population-based study with a longitudinal follow-up to investigate the genetic and molecular basis of age-related common chronic conditions and their interaction with life style and environment in the general population. The study was approved by the Ethics Committee of the Autonomous Province of Bolzano.

CHS

The Cardiovascular Health Study (CHS) is a population-based cohort study of risk factors for coronary heart disease and stroke in adults ≥65 years conducted across four field centers. The original predominantly European ancestry cohort of 5,201 persons was recruited in 1989-1990 from random samples of the Medicare eligibility lists; subsequently, an additional predominantly African-American cohort of 687 persons was enrolled for a total sample of 5,888.

Blood samples were drawn from all participants at their baseline examination and DNA was subsequently extracted from available samples. Genotyping was performed at the General Clinical Research Center's Phenotyping/Genotyping Laboratory at Cedars-Sinai among CHS participants who consented to genetic testing and had DNA available using the Illumina 370CNV BeadChip system (for European ancestry participants, in 2007) or the Illumina HumanOmni1-Quad_v1 BeadChip system (for African-American participants, in 2010).

CHS was approved by institutional review committees at each field center and individuals in the present analysis had available DNA and gave informed consent including consent to use of genetic information for the study of cardiovascular disease.

CROATIA-Korcula

The CROATIA-Korcula study sampled Croatians from the Adriatic island of Korcula, between the ages of 18 and 88. The fieldwork was performed in 2007 in the eastern part of the island, targeting healthy volunteers from the town of Korčula and the villages of Lumbarda, Žrnovo and Račišće.

CROATIA-Split

The CROATIA-Split study sampled Croatians from the city of Split, between the ages 18 and 85. The data was collected in 2008.

deCODE

The deCODE electrocardiogram (ECG) study was approved by the Data Protection Commission of Iceland and the National Bioethics Committee of Iceland (VSNb2015030024/03.01 with amendments). Written informed consent was obtained from individuals donating samples. Personal identifiers associated with medical information and samples were encrypted with a third-party encryption system as provided by the Data Protection Commission of Iceland. ECGs obtained in Landspitali - The National University Hospital of Iceland, Reykjavik, the largest and only tertiary care hospital in Iceland, have been digitally stored since 1998. For this analysis we used information on PR interval duration in milliseconds from individuals' first sinus rhythm ECG, including 80,085 individuals. We excluded individuals with permanent pacemakers or history of Wolff–Parkinson–White syndrome or atrioventricular block. We removed extreme outliers (PR-interval < 80 ms and PR-interval > 320ms) and adjusted the ECG measurements for sex, the R-R interval, year of birth and age at measurement. The genotypes in the deCODE study were derived from whole-genome sequencing of 8,383 Icelanders using Illumina standard TruSeq methodology to a mean depth of 30X, with subsequent imputation into 150,000 chip-typed individuals and their close relatives. The variants were then matched with variants found in 1000g phase 3 or the Haplotype Consortium reference panel. We tested the variants for association with PR interval duration in our data using linear regression. We assume that the quantitative trait follows a normal distribution with a mean that depends linearly on the expected allele at the SNP and a

variance covariance matrix proportional to the kinship matrix. We used LD score regression to account for inflation in the test statistics.

ERF

The Erasmus Rucphen Family (ERF) study is comprised of a family-based cohort embedded in the Genetic Research in Isolated Populations (GRIP) program in the southwest of the Netherlands. The aim of this program is to identify genetic risk factors for the development of complex disorders. In ERF, twenty-two families that had a large number of children baptized in the community church between 1850 and 1900 were identified with the help of detailed genealogical records. All living descendants of these couples, and their spouses, were invited to take part in the study. Comprehensive interviews, questionnaires, and examinations were completed at a research center in the area; approximately 3,200 individuals participated. Examinations included 12 lead ECG measurements. Electrocardiograms were recorded on ACTA electrocardiographs (ESAOTE, Florence, Italy) and digital measurements of the PR interval were made using the Modular ECG Analysis System (MEANS). Data collection started in June 2002 and was completed in February 2005.

FHS

The objective of the Framingham Heart Study (FHS) was to identify the common factors or characteristics that contribute to CVD by following its development over a long period of time in a large group of participants. The researchers recruited 5,209 men and women between the ages of 30 and 62 years from the town of Framingham, Massachusetts, and began the first round of extensive physical examinations and lifestyle interviews. Since 1948, participants

have continued to return to the study every two years for a detailed medical history, physical examination, and laboratory tests, and in 1971, the Study enrolled a second generation - 5,124 of the original participants' adult children and their spouses - to participate in similar examinations every four to eight years. In 1994, the need to establish a new study reflecting a more diverse community of Framingham was recognized, and the first Omni cohort of the Framingham Heart Study was enrolled. In April 2002 the Study entered a new phase, the enrollment of a third generation of participants, the grandchildren of the Original Cohort, who have been examined every four to eight years. In 2003, a second group of Omni participants was enrolled. Participants routinely received electrocardiograms at all research center visits. Electrocardiograms obtained at Original cohort Exam 11, Offspring cohort Exam 1, and Third Generation cohort Exam 1 were used in the PR analysis.

GAPP

GAPP is a population-based prospective cohort study involving a representative population-based sample of 2,170 healthy adults aged 25-41 years residing in the Principality of Liechtenstein. Exclusion criteria were the presence of cardiovascular disease, diabetes, obstructive sleep apnea and a body mass index >35kg/m2. A standardized 12-lead ECG was obtained in all participants. Details about the study have been described previously[3].

FINCAVAS

The purpose of the Finnish Cardiovascular Study (FINCAVAS) is to construct a risk profile - using genetic, haemodynamic and electrocardiographic (ECG) markers - of individuals at high risk of cardiovascular diseases, events and deaths. All patients scheduled for an exercise stress

test at Tampere University Hospital and willing to participate have been recruited between October 2001 and December 2007. The final number of participants is 4,567. In addition to repeated measurement of heart rate and blood pressure, digital high-resolution ECG at 500 Hz was recorded continuously during the entire exercise test, including the resting and recovery phases. About 20% of the patients were examined with coronary angiography. Genetic variations known or suspected to alter cardiovascular function or pathophysiology were analyzed to elucidate the effects and interactions of these candidate genes, exercise and commonly used cardiovascular medications.

GRAPHIC

Genetic Regulation of Arterial Pressure of Humans in the Community (GRAPHIC) Study: The GRAPHIC Study comprises 2024 individuals from 520 nuclear families recruited from the general population in Leicestershire, UK between 2003-2005 for the purpose of investigating the genetic determinants of blood pressure and related cardiovascular traits. Families were included if both parents aged 40-60 years and two offspring ≥18 years wished to participate. A detailed medical history was obtained from study subjects by standardized questionnaires and clinical examination was performed by research nurses following standard procedures. Measurements obtained included height, weight, waist-hip ratio, clinic and ambulatory blood pressure and a 12-lead ECG.

GS-SFHS

The GS:SFHS study recruited 23,960 participants aged 18–100 years between 2006–11; full details are reported elsewhere[4]. Participants came from across Scotland, with some family

members from further afield. The sample was 59% female, with a wide range of ages and socio-demographic characteristics. Most (87%) participants were born in Scotland and 96% in the UK or Ireland. Mean family size (excluding 1400 singletons without any relations in the study) was 4.05 members; median was 3 (IQR 2–5). The largest family had 36 participating members, and participants were grouped in 5573 families. Genome-wide genotype data for nearly one million genetic variants has been measured on 10,000 selected participants. An important feature of GS:SFHS is the breadth and depth of phenotype information, including clinical and physical measures and detailed data on cognitive function, personality traits and mental health. Participants were asked a series of questions on smoking history, from which current smokers, former smokers and never smokers can be defined.

HCHS/SOL

The Hispanic Community Health Study/ Study of Latinos (HCHS/SOL) is a multicenter, community-based cohort study of U.S. Hispanics/Latinos. Goals of the study are to examine the prevalence of and risk factors for several disorders including heart, lung, blood, and kidney phenotypes. HCHS/SOL investigators sampled 16,415 males and females aged 18-74 years at baseline from four study communities: The Bronx, NY, Chicago, IL, Miami, FL, and San Diego, CA. HCHS/SOL recruitment centers were selected so that the study would include at least 2,000 participants in each of the following designations: Mexican, Puerto Rican, Dominican, Cuban, and Central and South American. 11,686 participants consented to genetic studies and are included in this analysis.

Health 2000

In Health 2000 (BRIF8901), a nationally representative sample of persons aged 30 or over was drawn from the nationwide population register in Finland. The survey focused on collecting information on health and functional capacity of the population, and included questionnaires, interviews and a comprehensive health examination.

INGI-CARL

INGI-CARL consisted of about 1000 subjects who were drawn from Carlantino, an isolated village of southern Italy. Ethics approval was obtained from the Ethics Committee of the "IRCCS Burlo Garofolo" in Trieste. Written informed consent was obtained from every participant of the study. The study population had undergone clinical and instrumental evaluations between 1998 and 2005. For all subjects, anthropometrics variables (such as height, weight, etc) were taken and a structured questionnaire about lifestyle and medical history was filled out. In addition, blood pressure, body-mass index, biochemical analyses, ECG and cardiovascular evaluation were collected.

INGI-FVG

The INGI-FVG cohort consisted of about 1700 subjects drawn from the project "Genetic Park of Friuli Venezia Giulia". This study examined 6 isolated villages in the North-east of Italy between 2008 and 2010. Ethics approval was obtained from the Ethics Committee of the "IRCCS Burlo Garofolo" in Trieste. Written informed consent was obtained from every participant of the study. The study population had undergone clinical and instrumental

evaluations. For all subjects, anthropometrics variables (such as height, weight, etc) were taken and a structured questionnaire about lifestyle and medical history was filled out. In addition, blood pressure, body-mass index, biochemical analyses, ECG and cardiovascular evaluation were collected.

JHS

The JHS is a single-site cohort study of 5,306 extensively phenotyped African American women and men. Three clinical examinations have been completed, including the baseline examination, Examination 1 (2000–2004), Examination 2 (2005–2008), and Examination 3 (2009–2013), allowing comprehensive assessment of cardiovascular health and disease of the cohort at approximately four-year intervals. Ongoing monitoring of hospitalizations for cardiovascular events (coronary heart disease, heart failure and stroke) and deaths among cohort participants are accomplished by annual telephone follow-up interviews, surveillance of hospital discharge records (since 2000 for coronary heart disease and stroke, and since 2005 for heart failure), and vital records.

KORA F3

The KORA study is a series of independent population-based epidemiological surveys of participants living in the city of Augsburg, Southern Germany, or the two adjacent counties. All survey participants are residents of German nationality identified through the registration office and aged between 25 and 74 years at recruitment. The baseline survey KORA S3 was conducted in the years 1994/95. 3,006 participants from KORA S3 were reexamined in a 10-year follow-up (KORA F3) in the years 2004/05.

KORA S4

The KORA study is a series of independent population-based epidemiological surveys of participants living in the city of Augsburg, Southern Germany, or the two adjacent counties. All survey participants are residents of German nationality identified through the registration office and aged between 25 and 74 years at recruitment. The baseline survey KORA S4 was conducted in the years 1999-2001.

LifeLines

The LifeLines Cohort Study, and generation and management of GWAS genotype data for the LifeLines Cohort Study is supported by the Netherlands Organization of Scientific Research NWO (grant 175.010.2007.006), the Economic Structure Enhancing Fund (FES) of the Dutch government, the Ministry of Economic Affairs, the Ministry of Education, Culture and Science, the Ministry for Health, Welfare and Sports, the Northern Netherlands Collaboration of Provinces (SNN), the Province of Groningen, University Medical Center Groningen, the University of Groningen, Dutch Kidney Foundation and Dutch Diabetes Research Foundation.

MESA

The Multi-Ethnic Study of Atherosclerosis (MESA) is a study of the characteristics of subclinical cardiovascular disease (disease detected non-invasively before it has produced clinical signs and symptoms) and the risk factors that predict progression to clinically overt cardiovascular disease or progression of the subclinical disease. The cohort is a diverse, population-based sample of 6,814 asymptomatic men and women aged 45-84. Approximately

38 percent of the recruited participants are white, 28 percent African-American, 22 percent Hispanic, and 12 percent Asian (predominantly of Chinese descent). Participants were recruited during 2000-2002 from 6 field centers across the U.S. (at Wake Forest University; Columbia University; Johns Hopkins University; the University of Minnesota; Northwestern University; and the University of California – Los Angeles). All underwent anthropomorphic measurement and extensive evaluation by questionnaires at baseline, followed by 4 subsequent examinations at intervals of approximately 2-4 years. Age and sex were self-reported.

MICROS

The MICROS study is a population-based survey on adult volunteer participants who reside in three isolated villages in South Tyrol, Italy. These villages were selected because they had a small number of founders with old settlement, high rates of endogamy as well as slow/null population expansion. Extensive data was collected in 2002-03 regarding genealogy, and clinical measurements as well as collection of blood and urine samples and DNA isolation. An extensive standardized questionnaire was administered by interviewers to collect data on family history of disease and lifestyle exposures such as smoking and alcohol consumption. A serum sample was collected, prepared and stored at -80°C for subsequent analysis. The study was approved by the Ethics Committee of the Autonomous Province of Bolzano.

NEO

The NEO was designed for extensive phenotyping to investigate pathways that lead to obesity-related diseases. The NEO study is a population-based, prospective cohort study that includes 6,671 individuals aged 45–65 years, with an oversampling of individuals with overweight or

obesity. At baseline, information on demography, lifestyle, and medical history have been collected by questionnaires. In addition, samples of 24-h urine, fasting and postprandial blood plasma and serum, and DNA were collected. Genotyping was performed using the Illumina HumanCoreExome chip, which was subsequently imputed to the 1000 genome reference panel. Participants underwent an extensive physical examination, including anthropometry, electrocardiography, spirometry, and measurement of the carotid artery intima-media thickness by ultrasonography. The collection of data started in September 2008 and completed at the end of September 2012. Participants are currently being followed for the incidence of obesity-related diseases and mortality.

ORCADES

The Orkney Complex Disease Study (ORCADES) is a family-based, cross-sectional study that seeks to identify genetic factors influencing cardiovascular and other disease risk in the isolated archipelago of the Orkney Isles in northern Scotland[5]. Genetic diversity in this population is decreased compared to Mainland Scotland, consistent with the high levels of endogamy historically. 2078 participants aged 16-100 years were recruited between 2005 and 2011, most having three or four grandparents from Orkney, the remainder with two Orcadian grandparents. Fasting blood samples were collected and many health-related phenotypes and environmental exposures were measured in each individual. All participants gave written informed consent and the study was approved by Research Ethics Committees in Orkney and Aberdeen (North of Scotland REC).

PIVUS

The Prospective Investigation of Vasculature in Uppsala Seniors study was initiated in 2001 to investigate the predictive power of different measurements of vascular characteristics for future cardiovascular events, and secondary aims included measurements of cardiac and metabolic function, as well as serum biomarkers and levels of environmental pollutants. All individuals aged 70 living in the community of Uppsala in Sweden were deemed eligible for the study. The subjects were selected from the community register and invited in randomized order between April 2001 and June 2004. They received an invitation letter for participation within 2 months of their 70th birthday. Of the 2,025 subjects invited, 1,016 (507 male, 509 female) subjects agreed to participate. The participants were asked to answer a questionnaire about their medical history, smoking habits and regular medication.

PREVEND

PREVEND (Prevention of Renal and Vascular End-stage Disease) study is an ongoing prospective study investigating the natural course of increased levels of urinary albumin excretion and its relation to renal and cardiovascular disease. Details of the protocol have been described elsewhere.

PROSPER

All data come from the PROspective Study of Pravastatin in the Elderly at Risk (PROSPER). A detailed description of the study has been published elsewhere. PROSPER was a prospective multicenter randomized placebo-controlled trial to assess whether treatment with pravastatin

diminishes the risk of major vascular events in elderly. Between December 1997 and May 1999, we screened and enrolled subjects in Scotland (Glasgow), Ireland (Cork), and the Netherlands (Leiden). Men and women aged 70-82 years were recruited if they had pre-existing vascular disease or increased risk of such disease because of smoking, hypertension, or diabetes. A total number of 5,804 subjects were randomly assigned to pravastatin or placebo. A large number of prospective tests were performed including Biobank tests and cognitive function measurements. A whole genome wide screening has been performed in the sequential PHASE project. Of 5,763 subjects DNA was available for genotyping. Genotyping was performed with the Illumina 660K beadchip, after QC (call rate <95%) 5,244 subjects and 557,192 SNPs were left for analysis. These SNPs were imputed with IMPUTE software based on the 1000 Genomes Phase 3 panel. The study was approved by the institutional ethics review boards of centers of Cork University (Ireland), Glasgow University (Scotland) and Leiden University Medical Center (the Netherlands) and all participants gave written informed consent.

RS

Rotterdam Study (RS) is a prospective population-based cohort study. Details regarding design, objectives, and methods of the Rotterdam Study have been described in detail[6]. In short, the Rotterdam study started in 1989 with an initial cohort of 7,983 persons (out of 10,215 invitees; response rate 78%) 55 years of age or older living in the Ommoord district in the city of Rotterdam in the Netherlands. In 2000, 3,011 participants (out of 4,472 invitees, response rate 67%) who had become 55 years of age or moved into the study district were added to the cohort. Approximately every 4-5 years follow-up examinations are conducted. Examinations consist of a home interview and an extensive set of test at a research facility in the study district.

By linking the general practitioners' and municipality records to the study database, participants are continuously monitored for major morbidity and mortality.

SardiNIA

To identify genetic bases for prominent age-associated changes, including cardiovascular risk factors and determinants of personality traits, in a founder population. The results of the study will extend the studies of aging-associated conditions of outbred populations.

SHIP/SHIP-Trend

The Study of Health In Pomerania (SHIP) and SHIP-TREND both represent population-based studies. The Study of Health in Pomerania is a prospective longitudinal population-based cohort study in Western Pomerania assessing the prevalence and incidence of common diseases and their risk factors. Participants aged 20 to 79 with German citizenship and principal residency in the study area were recruited from a random sample of residents living in the three local cities, 12 towns as well as 17 randomly selected smaller towns. Individuals were randomly selected stratified by age and sex in proportion to population size of the city, town or small towns, respectively. A total of 4,308 participants were recruited between 1997 and 2001 in the SHIP cohort. Individuals were invited to the SHIP study centre for a computer-assisted personal interviews and extensive physical examinations.

TwinsUK

There are currently >13,500 twins registered participants in the TwinsUK study, of which over 9,000 are actively participating. The twins are aged 16 to 100 with approximately equal numbers of identical (MZ) and non-identical (DZ) twins and are predominantly female (80%) for historical reasons. Clinical, physiological, behavioural and lifestyle data is collected at either twin visits or via self-administered questionnaires, which volunteers complete either once or twice a year via the post or email. All studies have ethical approval from the Guy's and St Thomas' (GSTT) Ethics Committee.

UK Biobank

UK Biobank (UKB, www.ukbiobank.ac.uk) is a large longitudinal biobank study in the United Kingdom which was established to improve understanding of the genetic and environmental causes of common diseases including CVDs. In addition to self-reported disease outcomes and extensive health and life-style questionnaire data, UKB participants are being tracked through their NHS records and national registries (including cause of death and Hospital Episode Statistics). In 2017, UKB released the genotypes of 488,377 participants profiled with a custom SNP array. Genotyping QC was performed centrally by UKB, and genotypes imputed to Haplotype Reference Consortium (HRC) panel were released for 488,377 participants[7].

The PR interval was obtained from 4-lead ECGs (CAM-USB 6.5, Cardiosoft v6.51) recorded during a 15 second rest period prior to an exercise test while subjects were sitting on a stationary bike (eBike, Firmware v1.7). Electrodes were placed on the right and left antecubital fossae, and left and right wrist and the ECG was sampled at 500 Hz; lead I was used to derive the PR interval. To reduce the influence of noise, the PR interval was measured from a signal

averaged ECG waveform computed from the heartbeats available in the 15s trace. For this, we first identified the QRS-complexes using fully automatic in-house algorithms [8,9]. Before averaging, we removed ectopic beats and artefacts, as well as beats with an RR interval longer or shorter than 10ms compared to the mean RR interval were not included for averaging. A signal-averaged heartbeat was then computed from the remaining beats provided that the number of available beats was not less than 5. Finally, the PR interval was automatically measured as the interval between the onset of the P-wave and the onset of the QRS complex from the signal-averaged heartbeat. Cases with arbitrary P- or QRS-waves were reviewed manually and corrected or removed if necessary.

ULSAM

The Uppsala Longitudinal Study of Adult Men (ULSAM) cohort is a study of healthy elderly men in the Uppsala region of Sweden. It was initiated as a health screen focused at identifying metabolic risk factors for cardiovascular disease. In 1970, all 50 year old men living in Uppsala were invited to participate. Of these, 82% of them participated initially. The cohort was subsequently invited back at ages 60, 70 and 77. This study included the collection of a wide range of phenotypes, including blood pressure, insulin metabolism, weight and height, lipid markers, diet, cognitive function and socio-economic factors.

WHI

The Women's Health Initiative (WHI) is a long-term national health study focused on strategies for preventing heart disease, breast and colorectal cancer, and osteoporotic fractures in postmenopausal women. Launched in 1993, the WHI enrolled 68,132 women aged 50-79 into

one or more randomized Clinical Trials (CT), testing the health effects of hormone therapy (HT), dietary modification (DM), and/or calcium and Vitamin D supplementation (CaD). The genetic data used in this paper was generated by six ancillary studies: GWAS of Hormone Treatment and CVD and Metabolic Outcomes in WHI: Genomics and Randomized Trials Network (GARNET), Genome-wide Association Study of Nonsynonymous SNPs in Colon Cancer: Genetics and Epidemiology of Colorectal Cancer Consortium (GECCO), Genome-wide Association Study to Identify Genetic Components of Hip Fracture (HIPFX), Long Life Study (LLS), Modification of Particulate Matter-Mediated Arrhythmogenesis in Populations (MOPMAP), and Women's Health Initiative Memory Study (WHIMS). GARNET[10] is a case-control study of coronary heart disease, stroke, venous thromboembolism, and incident diabetes. GECCO[11] and HIPFX[12] are case-control studies of colon cancer and hip fracture. LLS[13] is a longitudinal study of the WHI Extension II Medical Records Cohort. MOPMAP[14] is a case-control study of ventricular ectopy. WHIMS[15] is a longitudinal study of cognitive decline, mild cognitive impairment, and dementia of the HT cohort.

YFS

The Young Finns study (YFS) is a population-based follow up-study started in 1980. The main aim of the YFS is to determine the contribution made by childhood lifestyle, biological and psychological measures to the risk of cardiovascular diseases in adulthood. In 1980, over 3,500 children and adolescents all around Finland participated in the baseline study. The follow-up studies have been conducted mainly with 3-year intervals. The 27-year follow-up study was conducted in 2007 (ages 30-45 years) with 2,204 participants. The study was approved by the local ethics committees (University Hospitals of Helsinki, Turku, Tampere, Kuopio and Oulu)

and was conducted following the guidelines of the Declaration of Helsinki. All participants gave their written informed consent.

(B) Definition of previously reported and previously not reported loci

A total of 98 uncorrelated ($r^2 < 0.1$) variants were reported in the literature for their association with PR interval (variants identified by studies in Europeans, Africans, Asians or combined ancestries)[10,11,16-21] at the start of this study. We calculated pairwise linkage disequilibrium (LD) in PLINK using the 1000 genomes (1000G) phase 3 all ancestry samples (N = 2,504)[22] for all variants within a 4Mb region centered on each previously reported variant using PLINK v1.9[23]. Multiple variants in LD ($r^2 > 0.1$) with each previously reported variant were ordered according to their positions on the chromosome, and a window was defined with the start position of the first variant in the ordered list and the end position of the last variant in the ordered list. The start and end of the window was then extended by 50kb on either side. This LD defined window or a window of ±500kb, which ever was the larger, was considered as a previously reported locus. Overlapping loci were merged and previously reported variants within the merged loci were ordered by their chromosomal position to define the start and end of the merged locus. Following this approach the 98 variants were grouped into 64 loci which we refer to as previously reported.

We followed a similar approach to define previously not reported loci. For each lead variant within a 1Mb region outside previously reported loci we calculated pairwise LD using the 1000G phase 3 all ancestry samples (N = 2,504) or European only (N = 503)[22] within a 4Mb region. Variants in LD ($r^2 > 0.1$) with each previously not reported variant were ordered according to their positions on the chromosome, and a window was defined with the start position of the first variant in the ordered list and the end position of the last variant in the ordered list. The start and end of the window was then extended by 50kb on either side. This LD defined window or a window of 1Mb, whichever was the larger, was considered as a previously not reported locus. We merged overlapping loci and then ordered variants within the merged loci by their position to define the start and end of the merged locus.

2. Supplementary Tables

Supplementary Table 1 Description of studies contributing to PR interval meta analyses: ancestry group, study design, sample size, and references.

Study short name	Study full name	Study reference (PMID or URL)	Ancestry	Study design	Total sample size
AGES*	Age, Gene/Environment Susceptibility Study	17351290	EA	population based	5664
AMISH*	The Amish Complex Disease Research Program	26374108; 18440328; 17261661; 15621217	EA	family based	1505
ARIC*	Atherosclerosis Risk in Communities	2646917	EA, AA	population based	12360
BAMBUÍ	Brazilian Bambuí Cohort Study of Ageing	26124090	BR	population based	485
BioMe	BioMe™ BioBank Program	25673413	EA, AA, HA	population based	2230
BRIGHT*	British Genetics of Hypertension	12826435	EA	hypertensive cases	2001
Broad AF	Broad AF study	29892015	EA	studies, case-control and case only	3461
CAMP (MGH CAMP)	Cardiology and Metabolic Patient Cohort	25812009	EA	cross sectional observational study	2140
CHRIS	Cooperative Health Research in South Tyrol	26541195	EA	population based	4205
CHS*	Cardiovascular Health Study	1669507	EA, AA	population based	5888
CROATIA-Korcula*	CROATIA-Korcula	19260139;19260141	EA	isolate population	898
CROATIA-Split*	CROATIA-Split	19260138	EA	population based	966
DeCODE*	DeCODE Electrocardiograph study	20062063	EA	population based	80085

ERF*	Erasmus Rucphen Family Study	15845033	EA	family based	3200
FHS*	Framingham Heart Study	14819398; 474565; 17372189	EA	population based or case-control	7834
FINCAVAS-MC	The Finnish Cardiovascular Study (Metabochip)	16515696	EA	consecutive patients undergoing an exercise stress test	4242
GAPP	Genetic and phenotypic determinants of blood pressure and other cardiovascular risk factors	23299990	EA	population based	1666
GRAPHIC	Genetic Regulation of Arterial Pressure of Humans in the Community	18443236	EA	population based	1017
GS-SFHS	Generation Scotland: Scottish Family Health Study	17014726	EA	population based with some families	897
HCHSSOL	Hispanic Community Health Survey/Study of Latinos	20609343	HA	multicenter, community-based cohort study	16415
H2000	Health 2000	http://urn.fi/URN:NBN:fi-fe201204193452	EA	population-based	7871
INGI-CARL*	INGI-Carlantino	23249956	EA	population based	326
INGI-FVG*	INGI - Friuli Venezia Giulia	23249956	EA	population based	981
JHS	Jackson Heart Study	16320381	AA	population based	5301
KORAF3*	Kooperative Gesundheitsforschung in der Region Augsburg	16032513; 16032514	EA	population based	3184
KORAS4*	Kooperative Gesundheitsforschung in der Region Augsburg	16032513; 16032514	EA	population based	4261
Lifelines*	LifeLines Cohort Study & Biobank	25502107	EA	population based	167729
MESA*	Multi-Ethnic Study of Atherosclerosis	12397006	EA, AA, AS	population based	6814
MICROS*	Microisolates in South Tyrol Study	17550581	EA	population based	602
NEO	Netherlands Epidemiology of Obesity	23576214	EA	population based (oversampling of participants with high BMI)	6673
ORCADES*	The Orkney Complex Disease Study	18760389	EA	population based	2215

PIVUS	Prospective Investigation of the Vasculature of Uppsala Seniors	16141402;http://www.medsci.uu.se/pivus/	EA	population based	834
Prevend*	Prevention of REnal and Vascular ENd-stage Disease	12356629	EA	population based	8592
PROSPER*	The PROspective Study of Pravastatin in the Elderly at Risk for vascular disease	12457784; 10569329; 21977987	EA	population based	5804
RS1*	Rotterdam Study cohort 1	22002080;26386597	EA	population based	6291
RS2*	Rotterdam Study cohort 2	22002080;26386597	EA	population based	2157
RS3*	Rotterdam Study cohort 3	22002080;26386597	EA	population based	3048
SardiNIA*	SardiNIA	16934002;https://sardinia.nia.nih.gov/	EA	population based	5933
SHIP-0*	Study of Health in Pomerania	20167617	EA	population based	4308
SHIP-T*	Study of Health in Pomerania - Trend	20167617	EA	population based	4420
TwinsUK*	TwinsUK	17254428;23088889	EA	population based	3086
UK Biobank	UK Biobank	30305743;25826379	EA	population based	502544
ULSAM	Uppsala Longitudinal Study of Adult Men	http://www.pubcare.uu.se/ulsam/	EA	population based	599
WHI	Women's Health Initiative	14575940	EA	population based	13134
YFS*	The Cardiovascular Risk in Young Finns Study	18263651;23069987	EA	population based	2063

EA, European ancestry; AA, African ancestry; HA, Hispanic ancestry; BR, Brazilians; AS, Asian ancestry. The studies indicated with * were included in a prior GWAS for PR interval (PMID:30046033). Note: The DeCODE sample in the prior study included 9,000 samples.

Supplementary Table 2 Association results between polygenic risk score for PR interval and 16 select cardiac phenotypes.

Trait	N (cases)	N (total samples)	Beta	SE	P	OR
Score derived from European ancestry only meta-analysis						
Atrial fibrillation	14812	309269	-0.047	0.009	4.30E-08	0.95
Atrial septal defect / patent foramen ovale	468	309234	0.017	0.046	0.7211	1.02
Distal conduction disease	2789	290252	0.103	0.019	7.02E-08	1.11
Atrioventricular preexcitation	307	309041	-0.168	0.057	0.0032	0.85
Congenital heart disease	902	309230	0.011	0.033	0.7420	1.01
Coronary artery disease	27072	309246	-0.012	0.007	0.0771	0.99
Heart failure	6076	309056	-0.002	0.013	0.8907	1.00
Hypertrophic cardiomyopathy	265	309248	0.018	0.061	0.7666	1.02
Implantable cardioverter defibrillator	633	309241	0.086	0.040	0.0312	1.09
Mitral valve prolapse	529	309246	0.093	0.044	0.0317	1.10
Non-ischemic cardiomyopathy	1703	305471	-0.051	0.024	0.0375	0.95
Pacemaker	3975	309270	0.062	0.016	0.0001	1.06
Sinus node dysfunction	287	290380	0.054	0.059	0.3574	1.06
Valve Disease	6244	309255	0.030	0.013	0.0192	1.03
Ventricular arrhythmia	2143	309263	-0.0054	0.022	0.8043	0.99
Ventricular premature depolarizations	412	309238	-0.060	0.049	0.2224	0.94
Score derived from multi-ancestry meta-analysis						
Atrial fibrillation	14812	309269	-0.058	0.009	1.30E-11	0.94
Atrial septal defect / patent foramen ovale	468	309234	0.007	0.046	0.8845	1.01
Distal conduction disease	2789	290252	0.105	0.019	3.18E-08	1.11
Atrioventricular preexcitation	307	309041	-0.191	0.057	8.36E-04	0.83
Congenital heart disease	902	309230	0.015	0.033	0.6462	1.02
Coronary artery disease	27072	309246	-0.014	0.007	0.0345	0.99
Heart failure	6076	309056	-0.002	0.013	0.9052	1
Hypertrophic cardiomyopathy	265	309248	0.031	0.061	0.6172	1.03
Implantable cardioverter defibrillator	633	309241	0.063	0.040	0.1108	1.07
Mitral valve prolapse	529	309246	0.078	0.043	0.0721	1.08
Non-ischemic cardiomyopathy	1703	305471	-0.049	0.024	0.0455	0.95
Pacemaker	3975	309270	0.056	0.016	0.0005	1.06
Sinus node dysfunction	287	290380	0.046	0.059	0.4381	1.05
Valve Disease	6244	309255	0.021	0.013	0.1001	1.02
Ventricular arrhythmia	2143	309263	-0.020	0.022	0.3573	0.98
Ventricular premature depolarizations	412	309238	-0.063	0.049	0.2035	0.94

Associations were tested by logistic regression model, adjusting for baseline age, sex, genotyping array, and trait-related principal components.
Beta, SE, and OR changes are based on per standard deviation increment of score.

3. Supplementary Figures

Supplementary Figure 1 Quantile-Quantile plots of autosomal variant results from meta-analyses of PR interval.

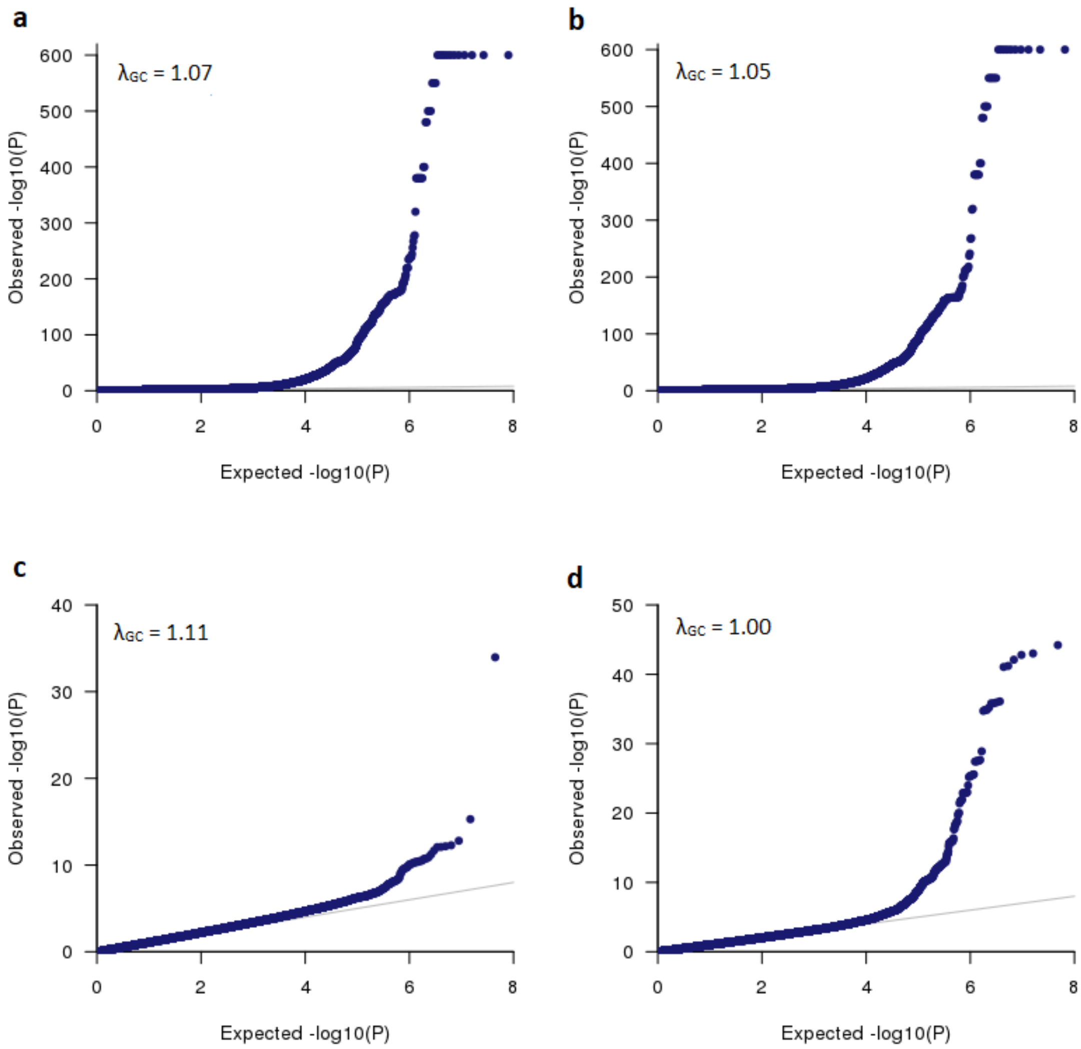

P values from multi-ancestry (a), European (b), African (c), and Hispanic/Latino ancestry (d) meta-analyses of autosomal variants for absolute PR interval (ms) are plotted on the $-\log_{10}$ scale for all variants. Sample sizes of each analysis are 293,051, 271,570, 8,173, and 11,686 for multi-ancestry, European, African, and Hispanic, respectively. The genomic inflation factors (λ_{GC}) are provided at the top left of each plot.

Supplementary Figure 2 Manhattan plot from the European (a), African (b), and Hispanic (c) meta-analyses of absolute PR interval.

(a)

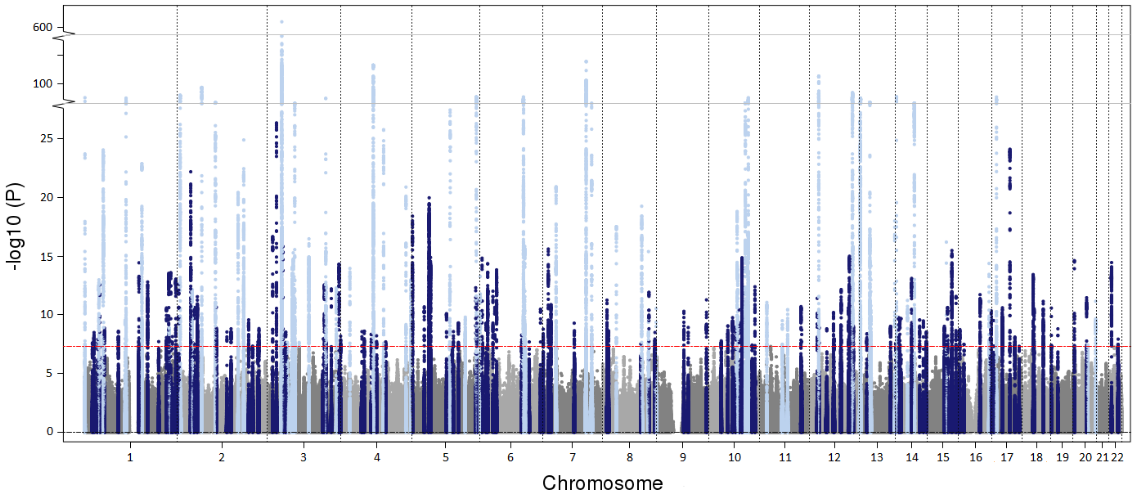

(b)

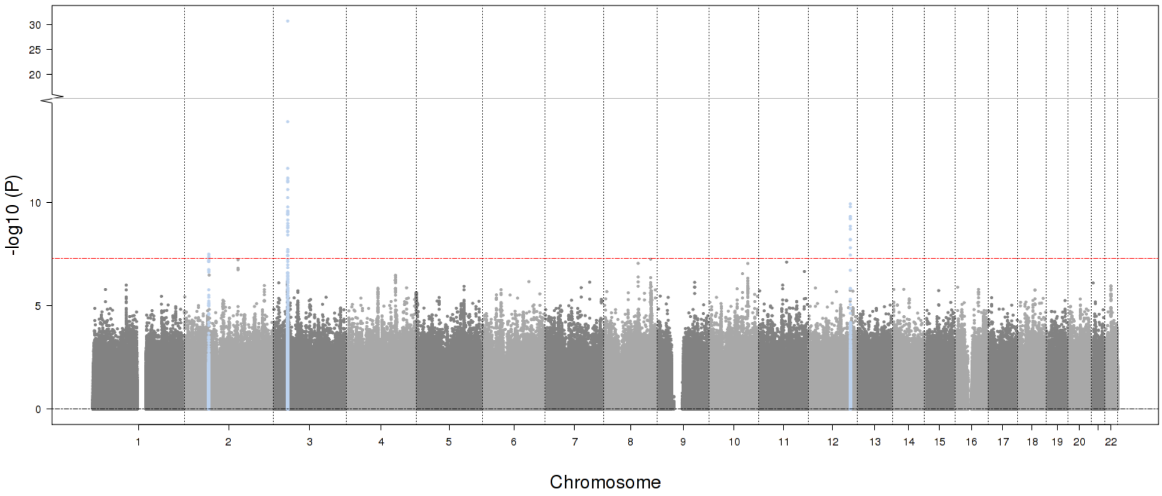

(c)

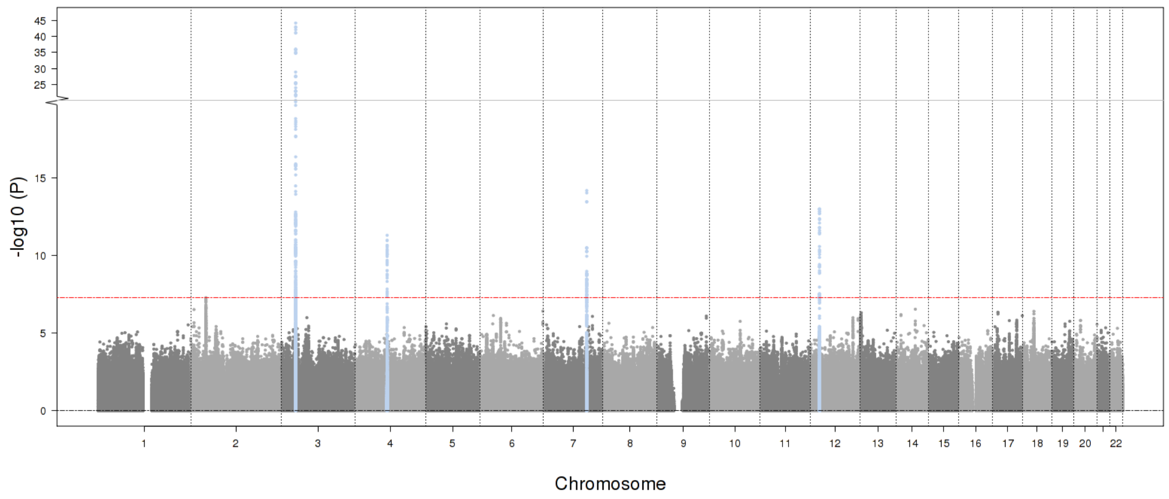

P values are plotted on the $-\log_{10}$ scale for all variants present in at least 60% of the maximum sample size from fixed-effect meta-analyses (N=271,570 for European-ancestry, N=8,173 for African-ancestry, and N=11,686 for Hispanic-ancestry). Associations of genome-wide significant ($P < 5\times10^{-8}$) variants at previously not reported and previously reported loci for PR interval are plotted in dark and light blue colors, respectively. In European meta-analysis 188 loci, of which 127 are newly identified and 61 were previously reported, reached genome-wide significance (Supplementary Data 6 and 8). Of the 127 previously not reported loci, 119 were also genome-wide significant in the multi-ancestry meta-analysis of PR interval. The remaining 8 loci were borderline genome-wide significant in the multi-ancestry meta-analysis (Supplementary Data 6 and 8). Four loci exceeded the genome-wide significance thresholds in both the African and Hispanic meta-analysis, all were previously reported (Supplementary Data 7).

Supplementary Figure 3 Region plots of 149 loci: 141 previously not reported loci from the multi-ancestry meta-analysis and 8 genome-wide significant loci in the European ancestry meta-analysis.

Previously not reported loci from Multi-ancestry meta-analysis

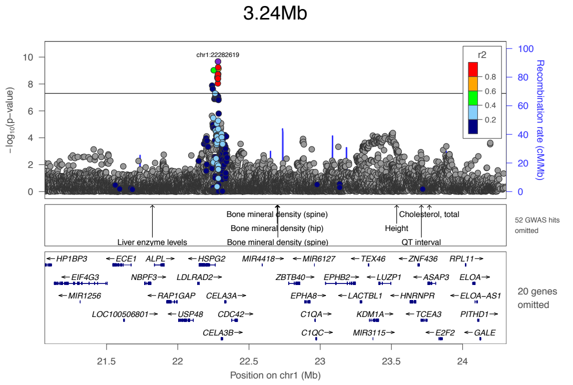

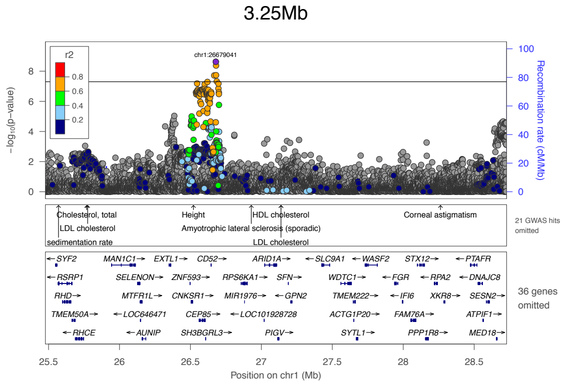

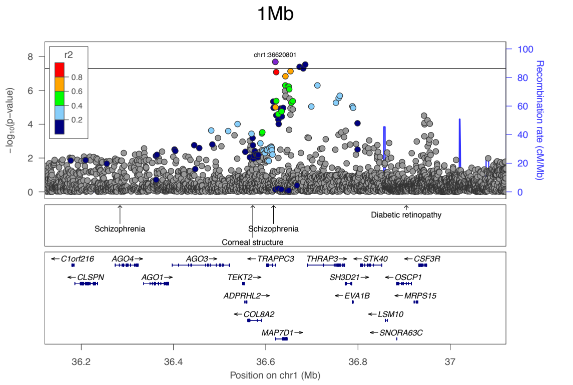

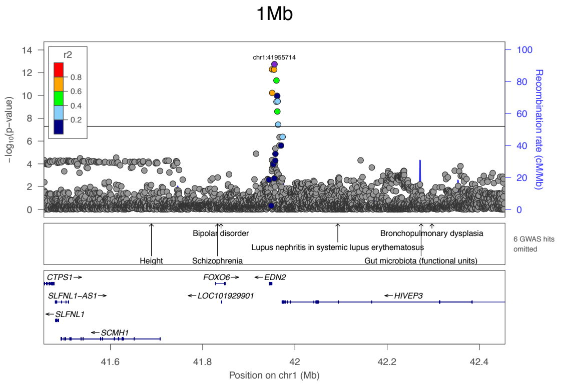

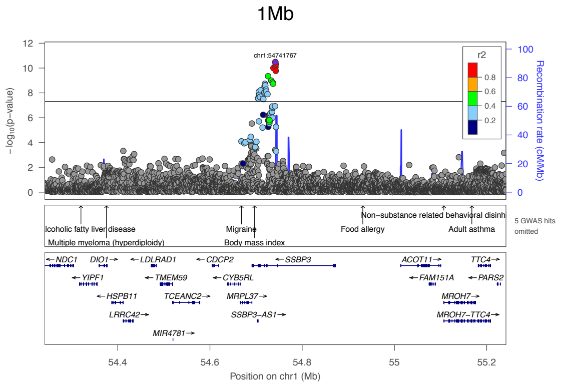

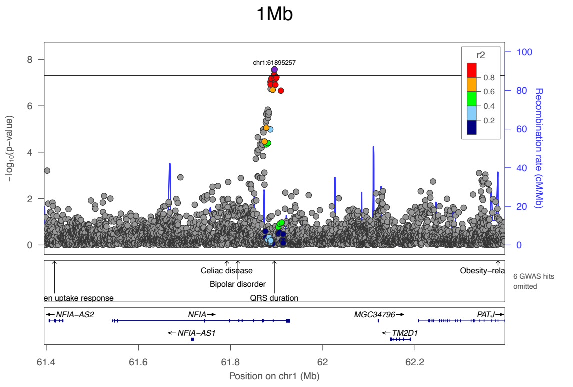

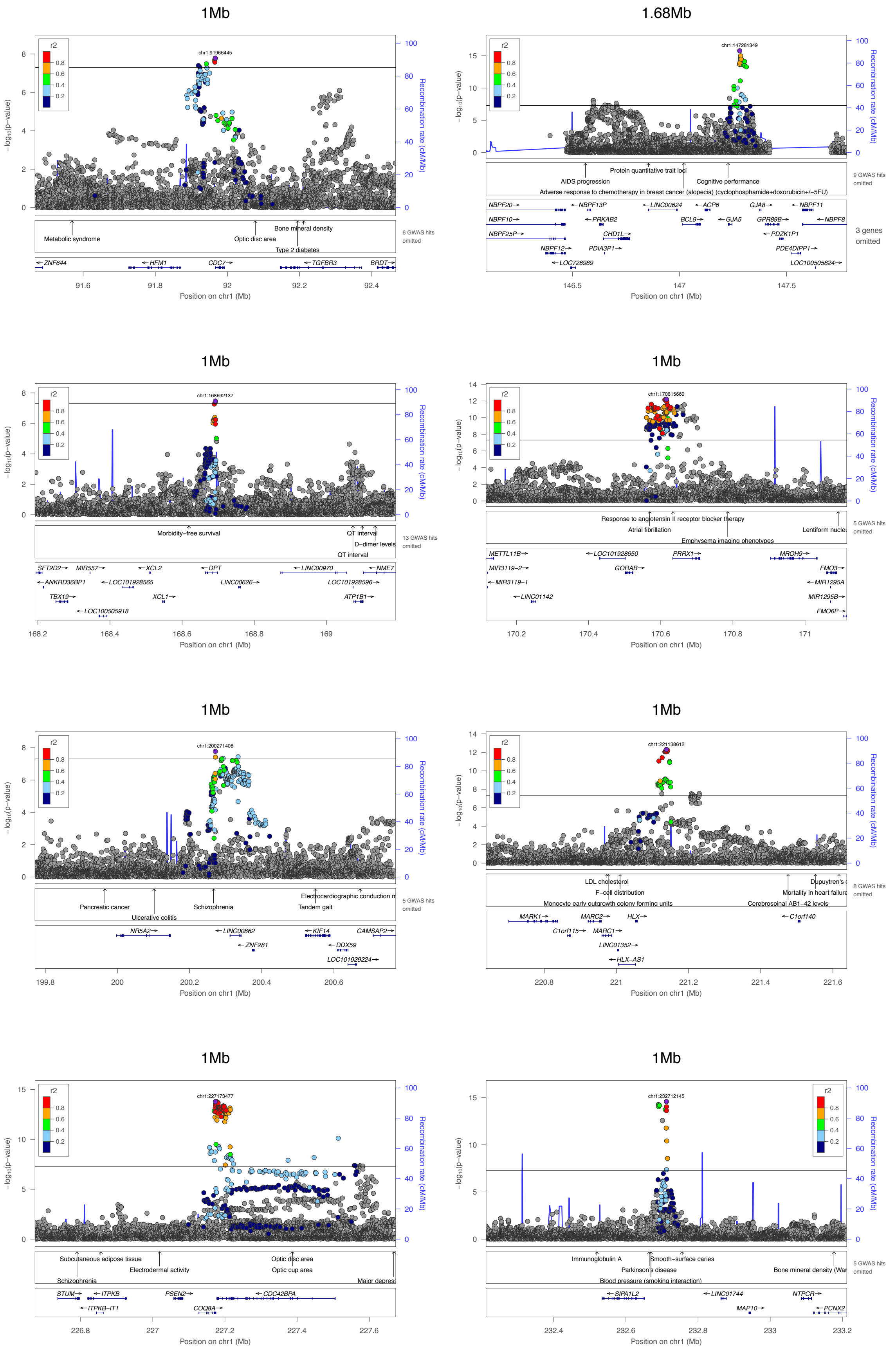
1Mb
1.68Mb
1Mb
1Mb
1Mb
1Mb
1Mb
1Mb
chr1:91966445
chr1:147281349
chr1:168692137
chr1:170615660
chr1:200271408
chr1:221138612
chr1:227173477
chr1:232712145
r2
−log10(p−value)
Recombination rate (cM/Mb)
Position on chr1 (Mb)
Metabolic syndrome
Optic disc area
Bone mineral density
Type 2 diabetes
6 GWAS hits omitted
Protein quantitative trait loci
AIDS progression
Cognitive performance
Adverse response to chemotherapy in breast cancer (alopecia) (cyclophosphamide+doxorubicin+/−5FU)
9 GWAS hits omitted
3 genes omitted
Morbidity−free survival
QT interval
D−dimer levels
13 GWAS hits omitted
Response to angiotensin II receptor blocker therapy
Atrial fibrillation
Emphysema imaging phenotypes
5 GWAS hits omitted
Pancreatic cancer
Ulcerative colitis
Schizophrenia
Electrocardiographic conduction
Tandem gait
5 GWAS hits omitted
LDL cholesterol
F−cell distribution
Monocyte early outgrowth colony forming units
Mortality in heart failure
Cerebrospinal AB1−42 levels
8 GWAS hits omitted
Subcutaneous adipose tissue
Electrodermal activity
Schizophrenia
Optic disc area
Optic cup area
Immunoglobulin A
Smooth−surface caries
Parkinson's disease
Blood pressure (smoking interaction)
Bone mineral density
5 GWAS hits omitted

2.18Mb

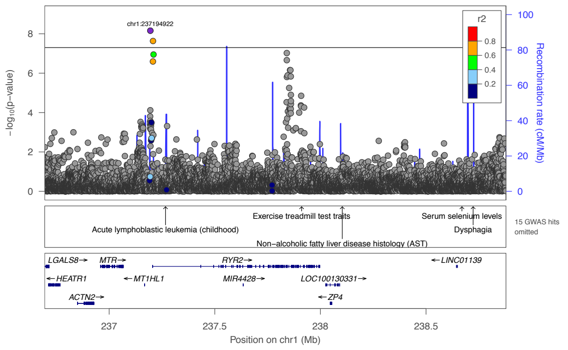

3.99Mb

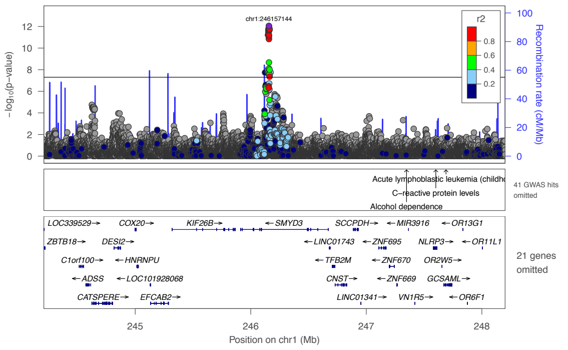

1Mb

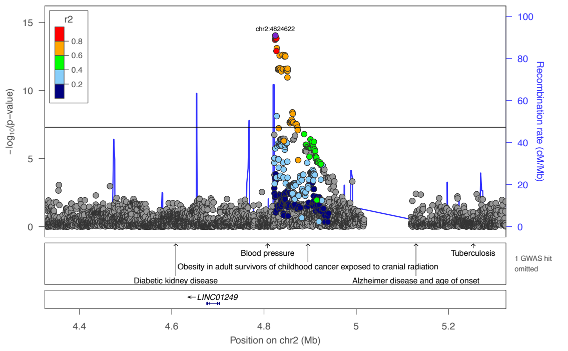

1Mb

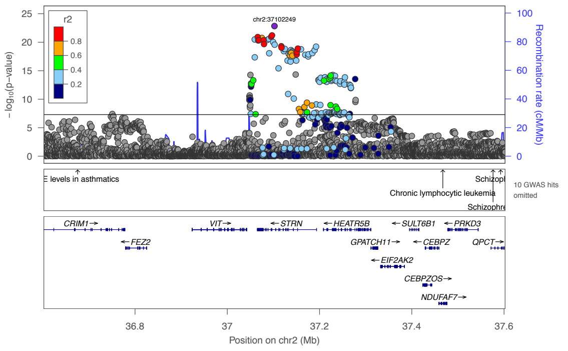

1Mb

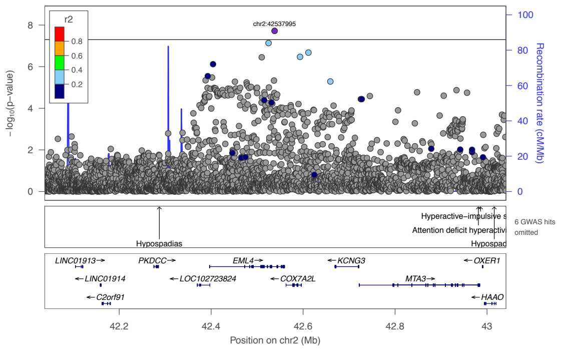

1Mb

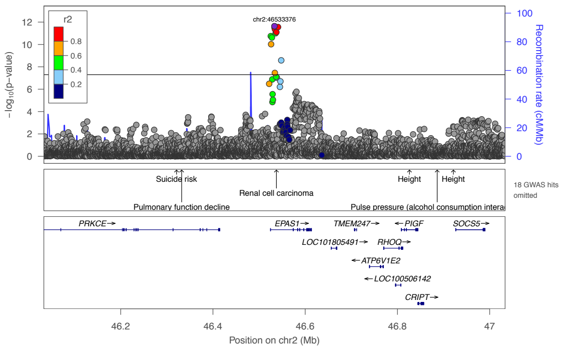

1.15Mb

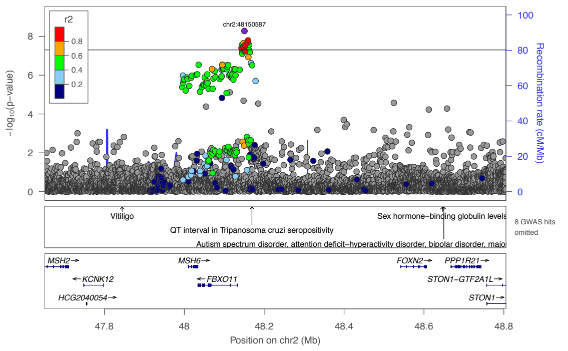

4.08Mb

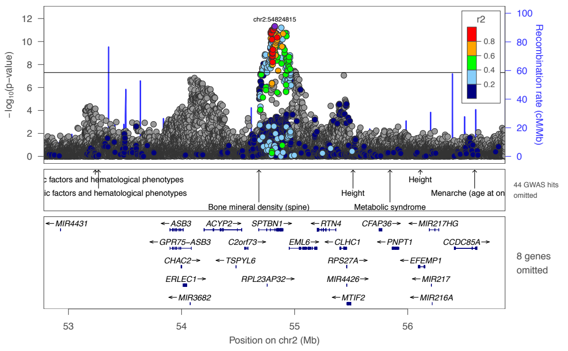

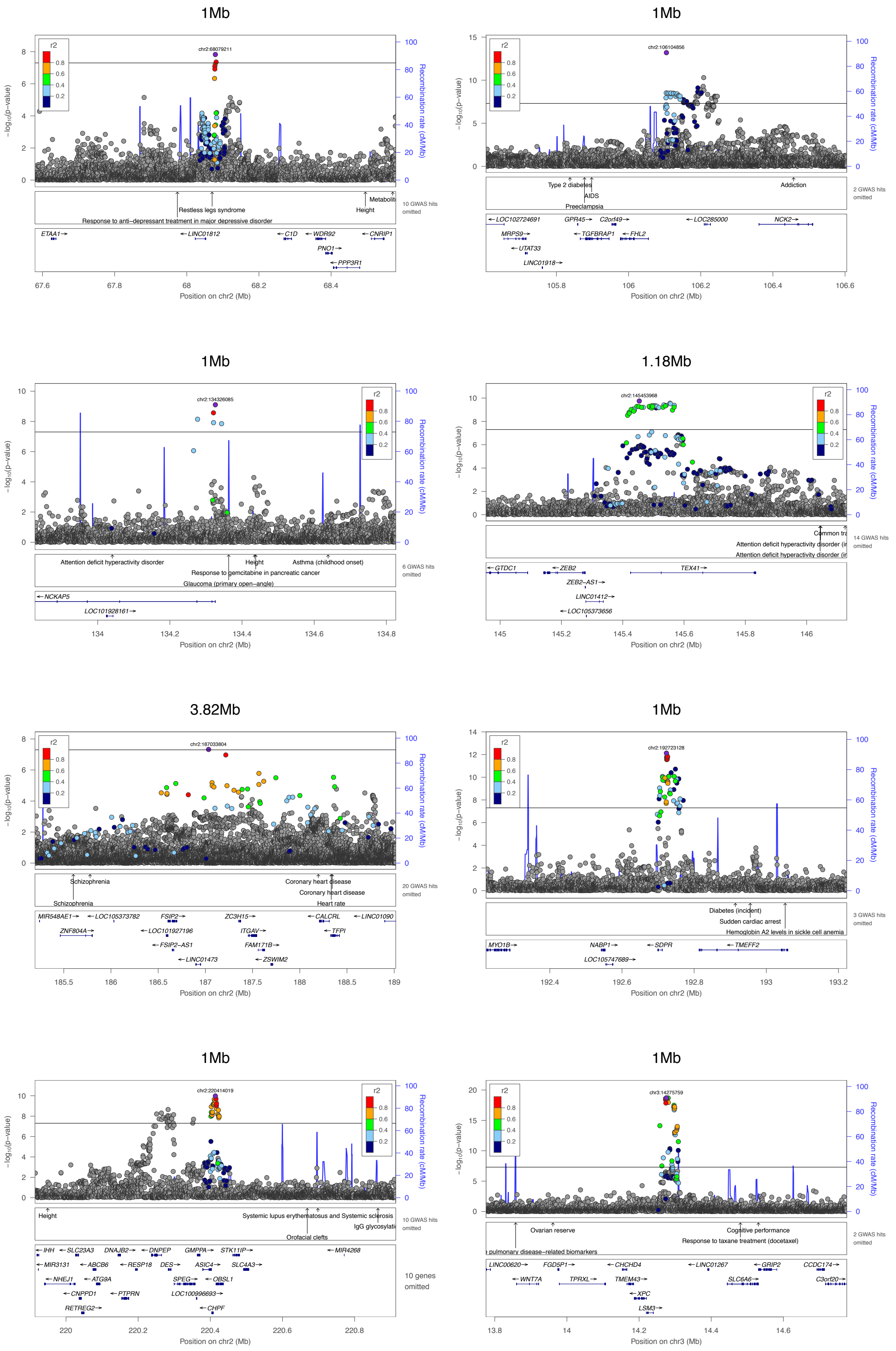
1Mb
chr2:68079211
r2
0.8
0.6
0.4
0.2
−log10(p−value)
Recombination rate (cM/Mb)
Metabolit
Restless legs syndrome
Height
Response to anti−depressant treatment in major depressive disorder
10 GWAS hits omitted
ETAA1
LINC01812
C1D
WDR92
CNRIP1
PNO1
PPP3R1
Position on chr2 (Mb)
1Mb
chr2:106104856
Type 2 diabetes
AIDS
Preeclampsia
Addiction
2 GWAS hits omitted
LOC102724691
GPR45
C2orf49
LOC285000
NCK2
MRPS9
TGFBRAP1
FHL2
UTAT33
LINC01918
Position on chr2 (Mb)
1Mb
chr2:134326085
Attention deficit hyperactivity disorder
Height
Asthma (childhood onset)
Response to gemcitabine in pancreatic cancer
Glaucoma (primary open−angle)
6 GWAS hits omitted
NCKAP5
LOC101928161
Position on chr2 (Mb)
1.18Mb
chr2:145453968
Common tra
Attention deficit hyperactivity disorder (ir
Attention deficit hyperactivity disorder (ir
14 GWAS hits omitted
GTDC1
ZEB2
TEX41
ZEB2−AS1
LINC01412
LOC105373656
Position on chr2 (Mb)
3.82Mb
chr2:187033804
Schizophrenia
Coronary heart disease
Heart rate
20 GWAS hits omitted
MIR548AE1
LOC105373782
FSIP2
ZC3H15
CALCRL
LINC01090
ZNF804A
LOC101927196
ITGAV
TFPI
FSIP2−AS1
FAM171B
LINC01473
ZSWIM2
Position on chr2 (Mb)
1Mb
chr2:192723128
Diabetes (incident)
Sudden cardiac arrest
Hemoglobin A2 levels in sickle cell anemia
3 GWAS hits omitted
MYO1B
NABP1
SDPR
TMEFF2
LOC105747689
Position on chr2 (Mb)
1Mb
chr2:220414019
Height
Systemic lupus erythematosus and Systemic sclerosis
IgG glycosylati
Orofacial clefts
10 GWAS hits omitted
IHH
SLC23A3
DNAJB2
DNPEP
GMPPA
STK11IP
MIR4268
MIR3131
ABCB6
RESP18
DES
ASIC4
SLC4A3
NHEJ1
ATG9A
SPEG
OBSL1
CNPPD1
PTPRN
LOC100996693
RETREG2
CHPF
10 genes omitted
Position on chr2 (Mb)
1Mb
chr3:14275759
Ovarian reserve
Cognitive performance
Response to taxane treatment (docetaxel)
pulmonary disease−related biomarkers
2 GWAS hits omitted
LINC00620
FGD5P1
CHCHD4
LINC01267
GRIP2
CCDC174
WNT7A
TPRXL
TMEM43
SLC6A6
C3orf20
XPC
LSM3
Position on chr3 (Mb)

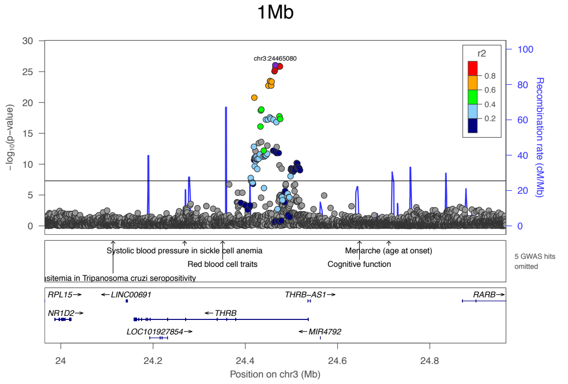
1Mb
chr3:24465080
-log10(p-value)
Recombination rate (cM/Mb)
r2
Systolic blood pressure in sickle cell anemia
Red blood cell traits
Menarche (age at onset)
Cognitive function
5 GWAS hits omitted
RPL15
LINC00691
THRB-AS1
RARB
NR1D2
THRB
LOC101927854
MIR4792
Position on chr3 (Mb)

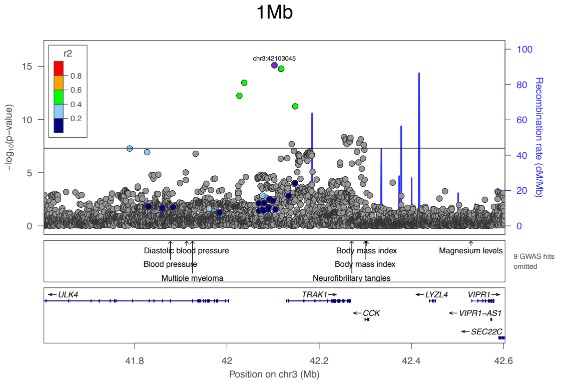
1Mb
chr3:42103045
-log10(p-value)
Recombination rate (cM/Mb)
r2
Diastolic blood pressure
Blood pressure
Multiple myeloma
Body mass index
Neurofibrillary tangles
Magnesium levels
9 GWAS hits omitted
ULK4
TRAK1
CCK
LYZL4
VIPR1
VIPR1-AS1
SEC22C
Position on chr3 (Mb)

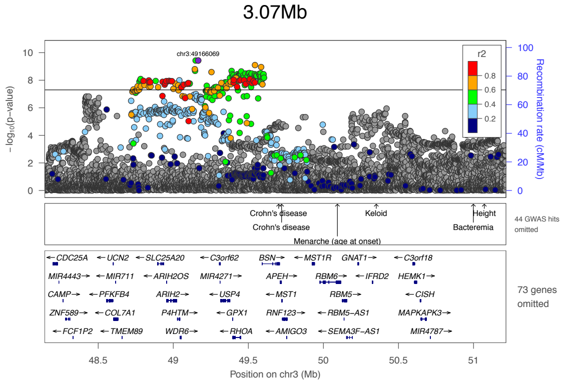
3.07Mb
chr3:49166069
-log10(p-value)
Recombination rate (cM/Mb)
r2
Crohn's disease
Keloid
Height
Bacteremia
Menarche (age at onset)
44 GWAS hits omitted
73 genes omitted
Position on chr3 (Mb)

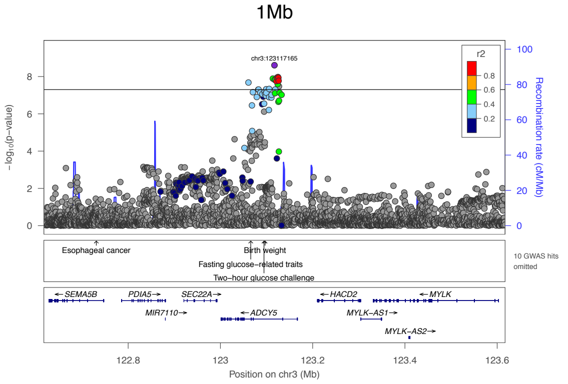
1Mb
chr3:123117165
-log10(p-value)
Recombination rate (cM/Mb)
r2
Esophageal cancer
Birth weight
Fasting glucose-related traits
Two-hour glucose challenge
10 GWAS hits omitted
SEMA5B
PDIA5
SEC22A
MIR7110
ADCY5
HACD2
MYLK
MYLK-AS1
MYLK-AS2
Position on chr3 (Mb)

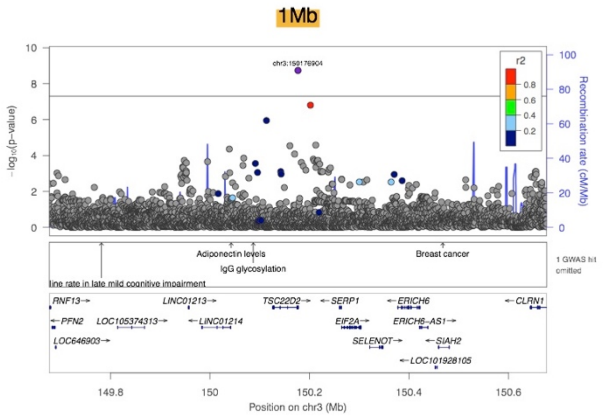
1Mb
chr3:150176904
-log10(p-value)
Recombination rate (cM/Mb)
r2
Adiponectin levels
IgG glycosylation
Breast cancer
1 GWAS hit omitted
RNF13
LINC01213
TSC22D2
SERP1
ERICH6
CLRN1
PFN2
LOC105374313
LINC01214
EIF2A
ERICH6-AS1
LOC646903
SELENOT
SIAH2
LOC101928105
Position on chr3 (Mb)

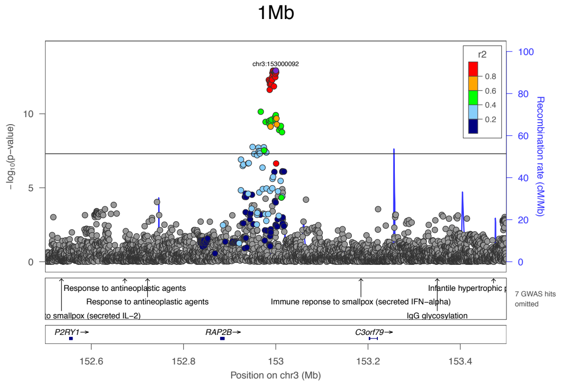
1Mb
chr3:153000092
-log10(p-value)
Recombination rate (cM/Mb)
r2
Response to antineoplastic agents
Immune reponse to smallpox (secreted IFN-alpha)
IgG glycosylation
7 GWAS hits omitted
P2RY1
RAP2B
C3orf79
Position on chr3 (Mb)

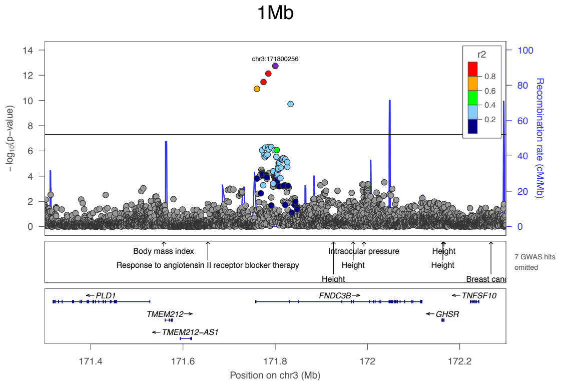
1Mb
chr3:171800256
-log10(p-value)
Recombination rate (cM/Mb)
r2
Body mass index
Response to angiotensin II receptor blocker therapy
Intraocular pressure
Height
7 GWAS hits omitted
PLD1
FNDC3B
TNFSF10
TMEM212
GHSR
TMEM212-AS1
Position on chr3 (Mb)

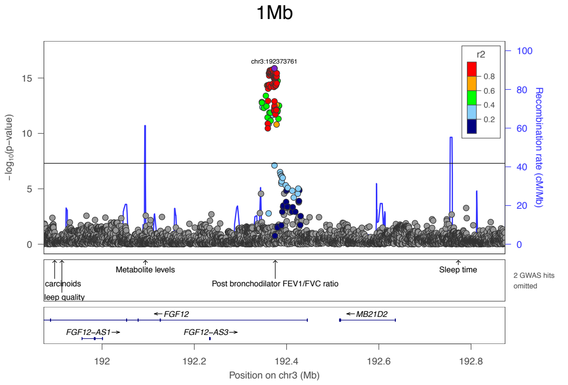
1Mb
chr3:192373761
-log10(p-value)
Recombination rate (cM/Mb)
r2
Metabolite levels
Post bronchodilator FEV1/FVC ratio
Sleep time
2 GWAS hits omitted
FGF12
MB21D2
FGF12-AS1
FGF12-AS3
Position on chr3 (Mb)

3.03Mb

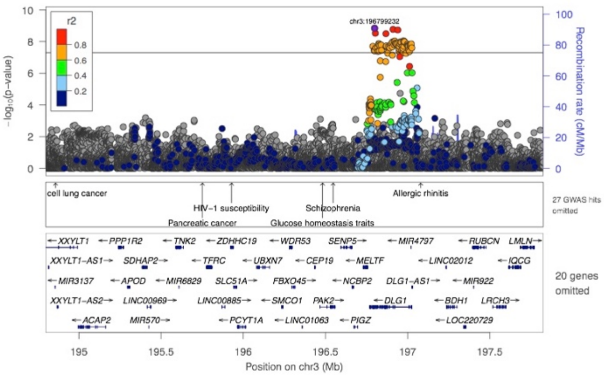

2.14Mb

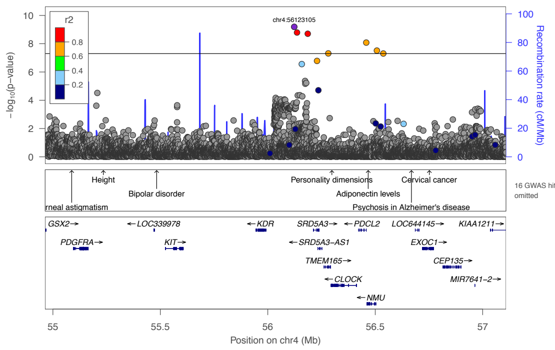

1Mb

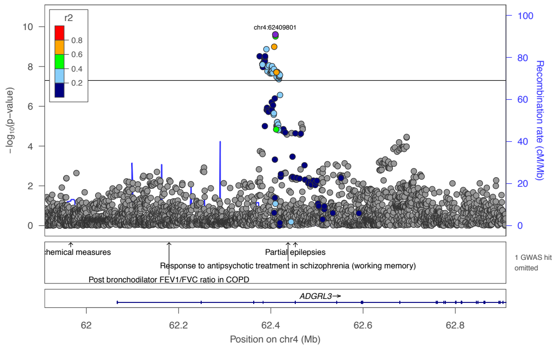

1Mb

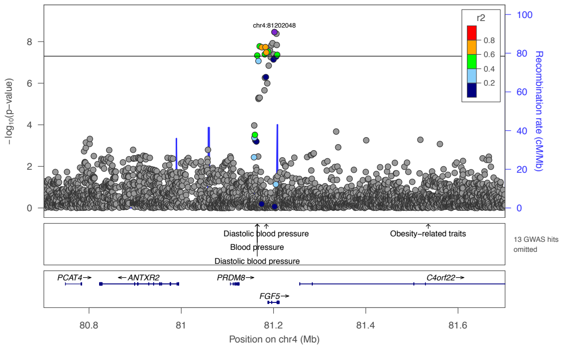

1Mb

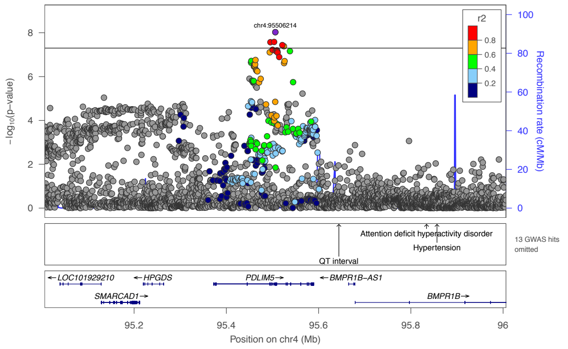

1.82Mb

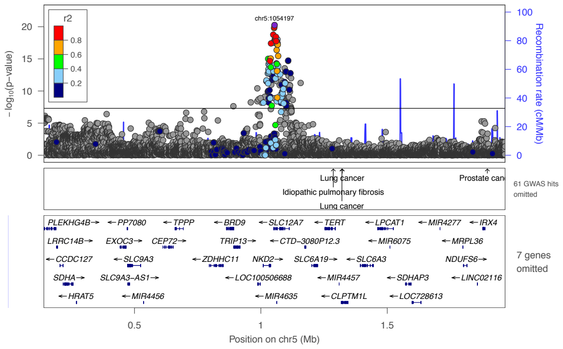

1.87Mb

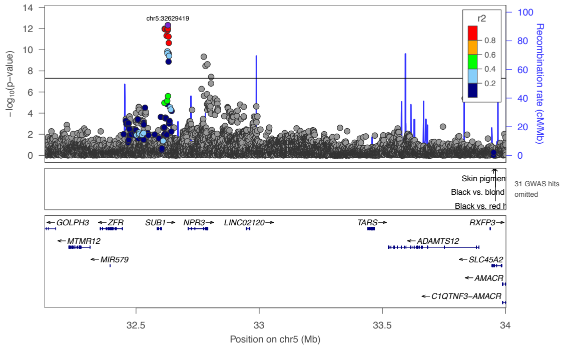

2.29Mb

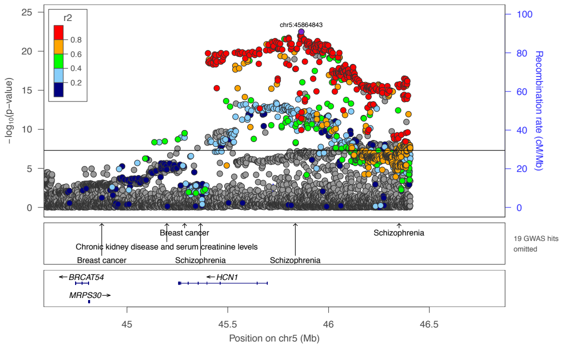

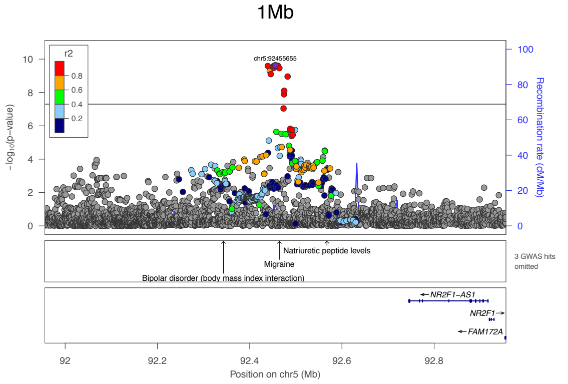
1Mb
chr5:92455655
r2
−log10(p−value)
Recombination rate (cM/Mb)
Natriuretic peptide levels
Migraine
Bipolar disorder (body mass index interaction)
3 GWAS hits omitted
NR2F1−AS1
NR2F1
FAM172A
Position on chr5 (Mb)

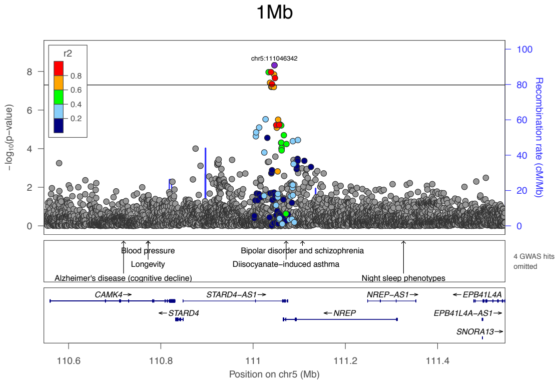
1Mb
chr5:111046342
r2
−log10(p−value)
Recombination rate (cM/Mb)
Blood pressure
Longevity
Alzheimer's disease (cognitive decline)
Bipolar disorder and schizophrenia
Diisocyanate−induced asthma
Night sleep phenotypes
4 GWAS hits omitted
CAMK4
STARD4
STARD4−AS1
NREP−AS1
NREP
EPB41L4A
EPB41L4A−AS1
SNORA13
Position on chr5 (Mb)

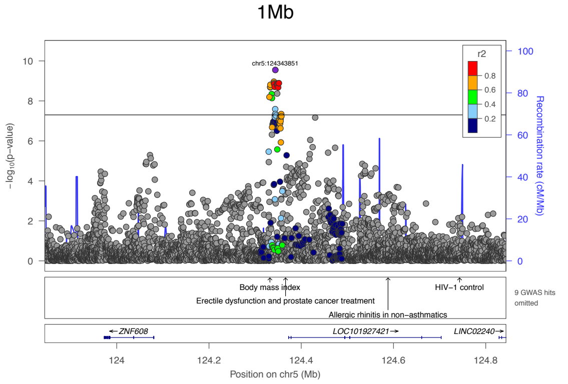
1Mb
chr5:124343851
r2
−log10(p−value)
Recombination rate (cM/Mb)
Body mass index
Erectile dysfunction and prostate cancer treatment
Allergic rhinitis in non−asthmatics
HIV−1 control
9 GWAS hits omitted
ZNF608
LOC101927421
LINC02240
Position on chr5 (Mb)

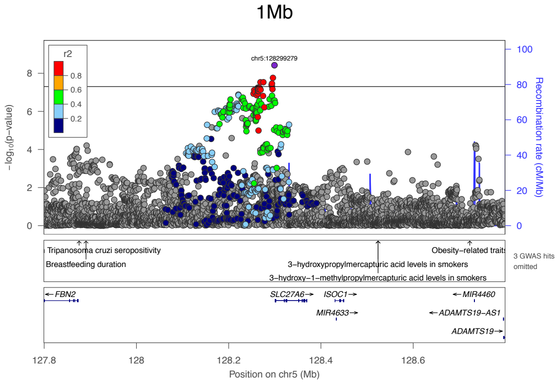
1Mb
chr5:128299279
r2
−log10(p−value)
Recombination rate (cM/Mb)
Tripanosoma cruzi seropositivity
Breastfeeding duration
Obesity−related traits
3−hydroxypropylmercapturic acid levels in smokers
3−hydroxy−1−methylpropylmercapturic acid levels in smokers
3 GWAS hits omitted
FBN2
SLC27A6
ISOC1
MIR4633
MIR4460
ADAMTS19−AS1
ADAMTS19
Position on chr5 (Mb)

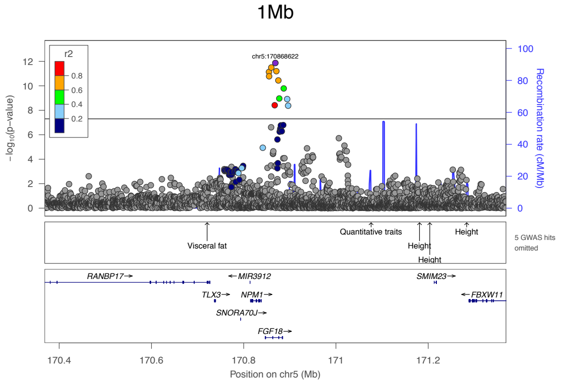
1Mb
chr5:170868622
r2
−log10(p−value)
Recombination rate (cM/Mb)
Visceral fat
Quantitative traits
Height
Height
Height
5 GWAS hits omitted
RANBP17
MIR3912
TLX3
NPM1
SNORA70J
FGF18
SMIM23
FBXW11
Position on chr5 (Mb)

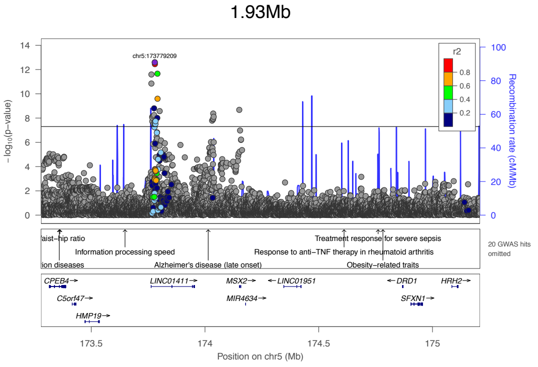
1.93Mb
chr5:173779209
r2
−log10(p−value)
Recombination rate (cM/Mb)
aist−hip ratio
ion diseases
Information processing speed
Alzheimer's disease (late onset)
Treatment response for severe sepsis
Response to anti−TNF therapy in rheumatoid arthritis
Obesity−related traits
20 GWAS hits omitted
CPEB4
C5orf47
HMP19
LINC01411
MSX2
MIR4634
LINC01951
DRD1
SFXN1
HRH2
Position on chr5 (Mb)

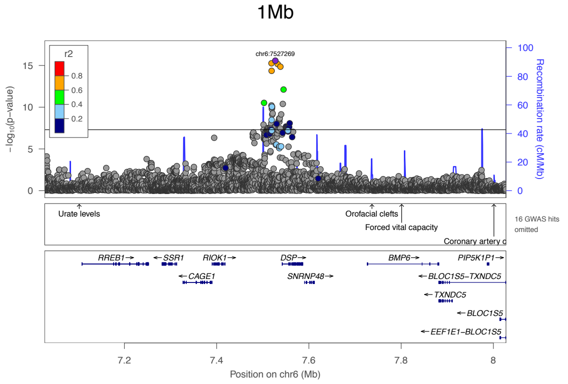
1Mb
chr6:7527269
r2
−log10(p−value)
Recombination rate (cM/Mb)
Urate levels
Orofacial clefts
Forced vital capacity
Coronary artery d
16 GWAS hits omitted
RREB1
SSR1
CAGE1
RIOK1
DSP
SNRNP48
BMP6
TXNDC5
PIP5K1P1
BLOC1S5−TXNDC5
BLOC1S5
EEF1E1−BLOC1S5
Position on chr6 (Mb)

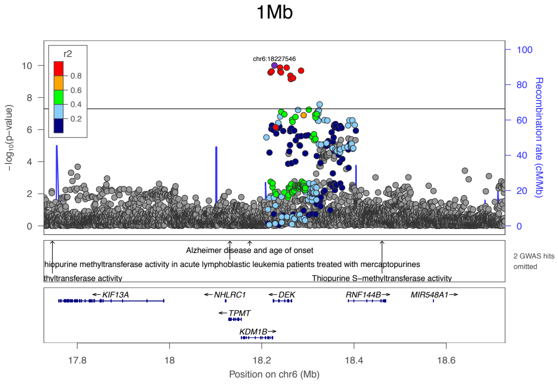
1Mb
chr6:18227546
r2
−log10(p−value)
Recombination rate (cM/Mb)
Alzheimer disease and age of onset
hiopurine methyltransferase activity in acute lymphoblastic leukemia patients treated with mercaptopurines
thyltransferase activity
Thiopurine S−methyltransferase activity
2 GWAS hits omitted
KIF13A
NHLRC1
TPMT
KDM1B
DEK
RNF144B
MIR548A1
Position on chr6 (Mb)

1Mb

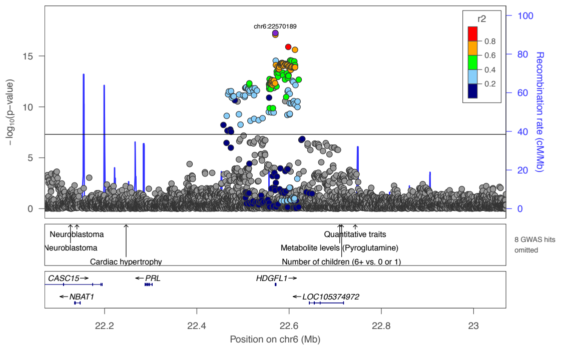

1Mb

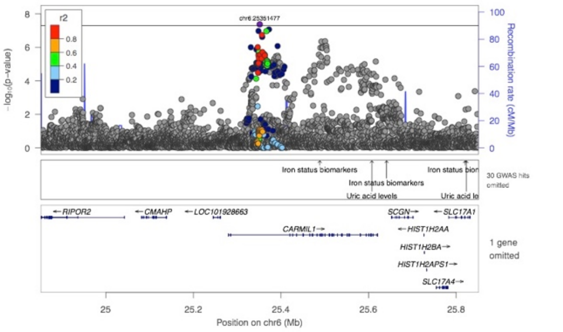

1Mb

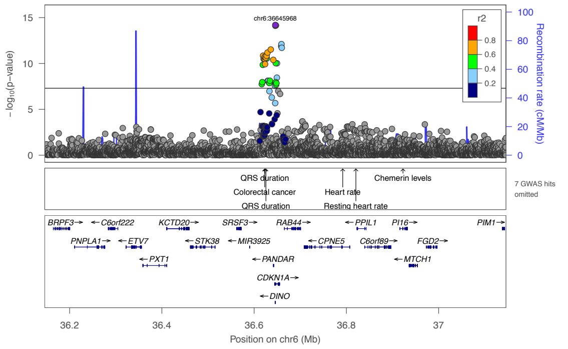

1Mb

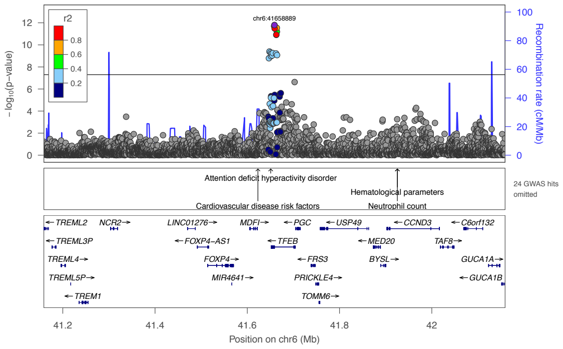

3.04Mb

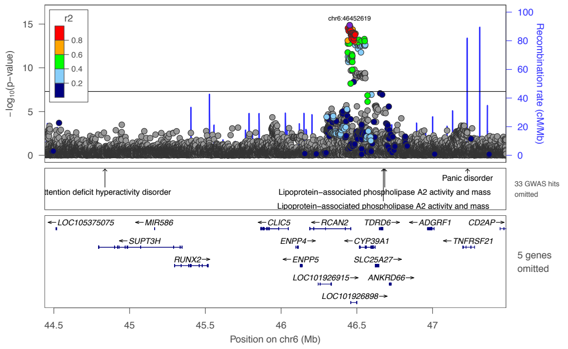

1Mb

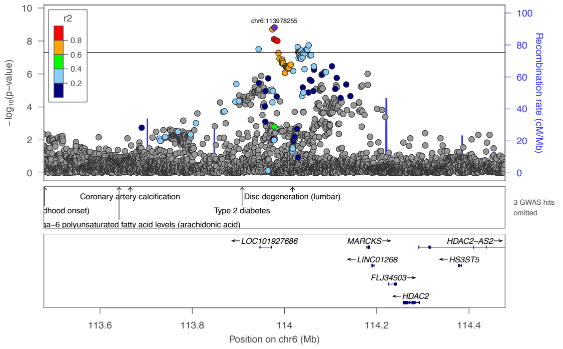

4.09Mb

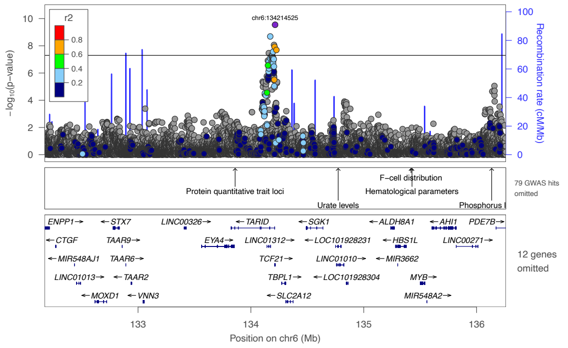

1Mb

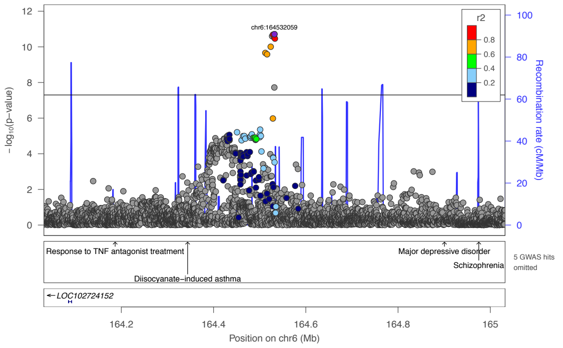

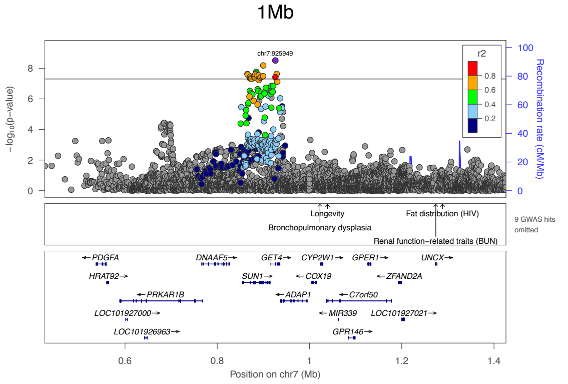

1Mb
chr7:925949
−log10(p−value)
Recombination rate (cM/Mb)
r2
Longevity
Bronchopulmonary dysplasia
Fat distribution (HIV)
Renal function−related traits (BUN)
9 GWAS hits omitted
PDGFA
HRAT92
PRKAR1B
LOC101927000
LOC101926963
DNAAF5
SUN1
GET4
COX19
ADAP1
CYP2W1
C7orf50
MIR339
GPR146
GPER1
ZFAND2A
LOC101927021
UNCX
Position on chr7 (Mb)

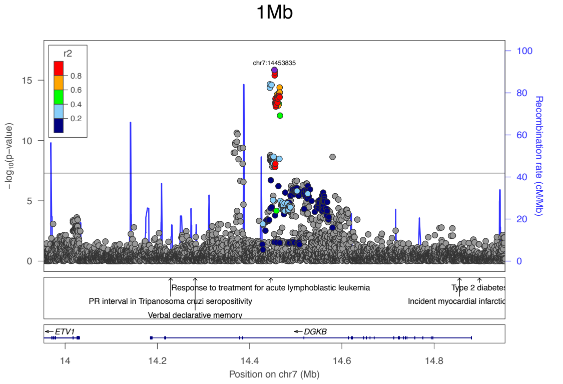

1Mb
chr7:14453835
−log10(p−value)
Recombination rate (cM/Mb)
r2
Response to treatment for acute lymphoblastic leukemia
PR interval in Tripanosoma cruzi seropositivity
Verbal declarative memory
Type 2 diabetes
Incident myocardial infarctio
ETV1
DGKB
Position on chr7 (Mb)

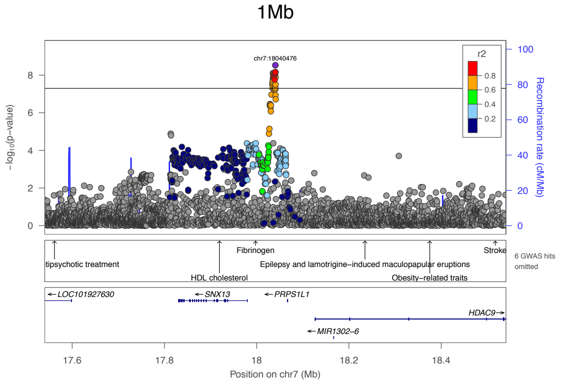

1Mb
chr7:18040476
−log10(p−value)
Recombination rate (cM/Mb)
r2
tipsychotic treatment
HDL cholesterol
Fibrinogen
Epilepsy and lamotrigine−induced maculopapular eruptions
Obesity−related traits
Stroke
6 GWAS hits omitted
LOC101927630
SNX13
PRPS1L1
HDAC9
MIR1302−6
Position on chr7 (Mb)

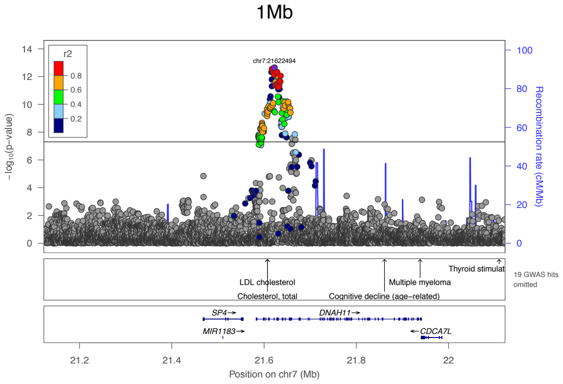

1Mb
chr7:21622494
−log10(p−value)
Recombination rate (cM/Mb)
r2
LDL cholesterol
Cholesterol, total
Cognitive decline (age−related)
Multiple myeloma
Thyroid stimulat
19 GWAS hits omitted
SP4
MIR1183
DNAH11
CDCA7L
Position on chr7 (Mb)

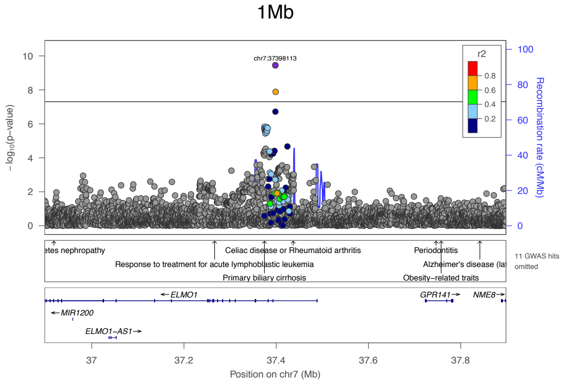

1Mb
chr7:37398113
−log10(p−value)
Recombination rate (cM/Mb)
r2
etes nephropathy
Response to treatment for acute lymphoblastic leukemia
Celiac disease or Rheumatoid arthritis
Primary biliary cirrhosis
Periodontitis
Alzheimer's disease (la
Obesity−related traits
11 GWAS hits omitted
ELMO1
MIR1200
ELMO1−AS1
GPR141
NME8
Position on chr7 (Mb)

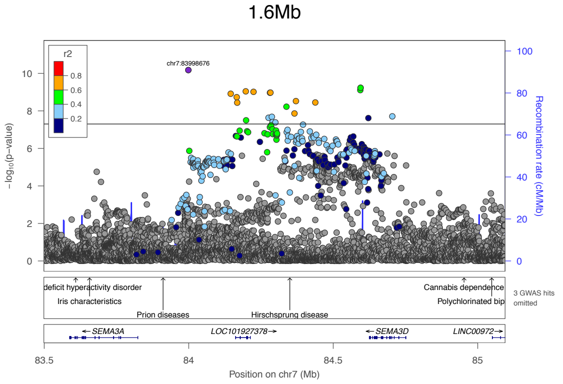

1.6Mb
chr7:83998676
−log10(p−value)
Recombination rate (cM/Mb)
r2
deficit hyperactivity disorder
Iris characteristics
Prion diseases
Hirschsprung disease
Cannabis dependence
Polychlorinated bip
3 GWAS hits omitted
SEMA3A
LOC101927378
SEMA3D
LINC00972
Position on chr7 (Mb)

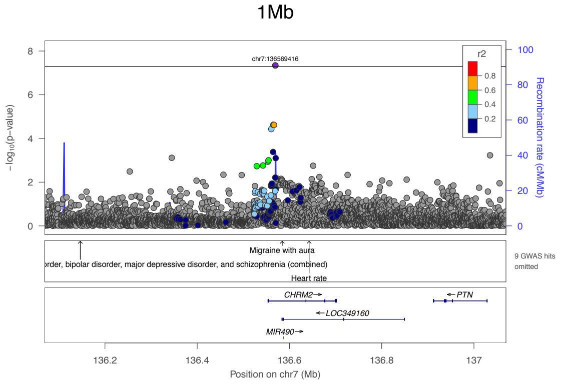

1Mb
chr7:136569416
−log10(p−value)
Recombination rate (cM/Mb)
r2
Migraine with aura
order, bipolar disorder, major depressive disorder, and schizophrenia (combined)
Heart rate
9 GWAS hits omitted
CHRM2
LOC349160
MIR490
PTN
Position on chr7 (Mb)

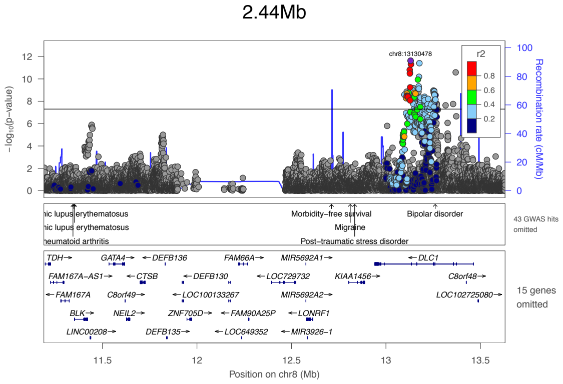

2.44Mb
chr8:13130478
−log10(p−value)
Recombination rate (cM/Mb)
r2
ic lupus erythematosus
nic lupus erythematosus
heumatoid arthritis
Morbidity−free survival
Migraine
Post−traumatic stress disorder
Bipolar disorder
43 GWAS hits omitted
TDH
FAM167A−AS1
FAM167A
BLK
LINC00208
GATA4
C8orf49
NEIL2
DEFB136
CTSB
DEFB135
DEFB130
LOC100133267
ZNF705D
FAM66A
LOC729732
FAM90A25P
LOC649352
MIR5692A1
MIR5692A2
LONRF1
MIR3926−1
KIAA1456
DLC1
C8orf48
LOC102725080
15 genes omitted
Position on chr8 (Mb)

1.65Mb

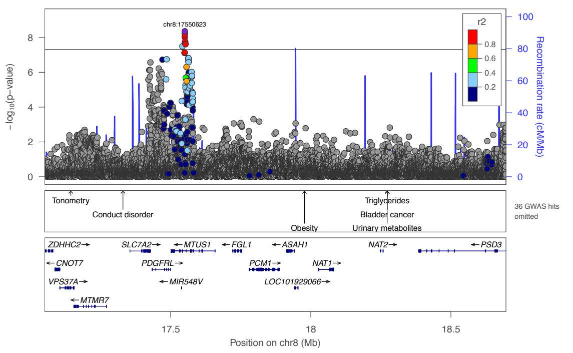

1Mb

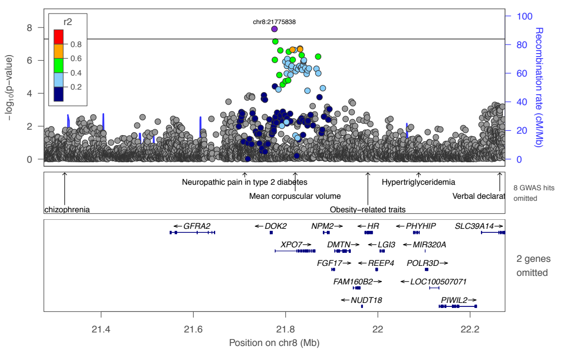

1Mb

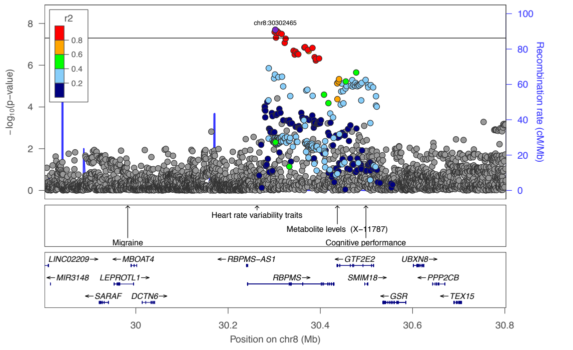

1.76Mb

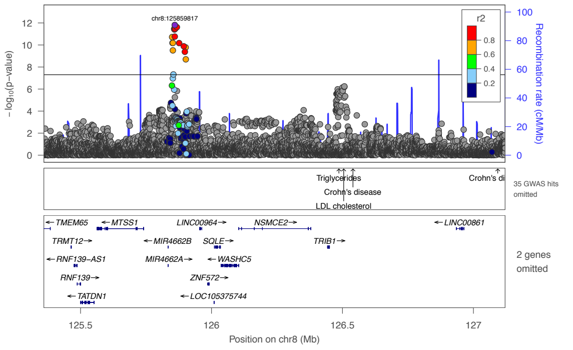

1.01Mb

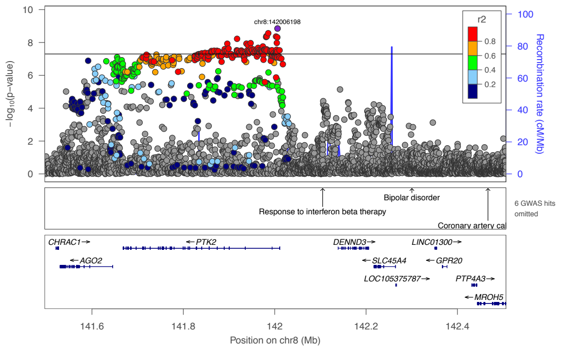

1Mb

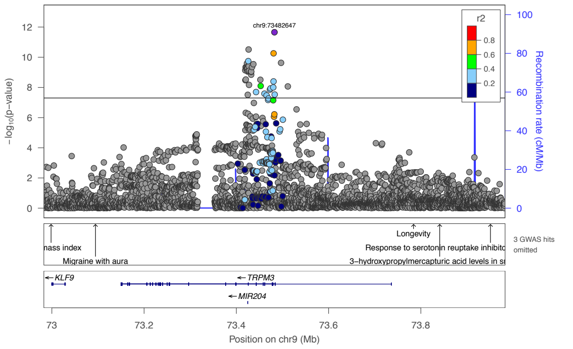

3.84Mb

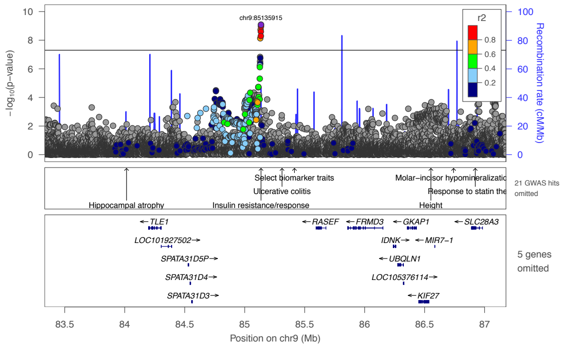

1Mb

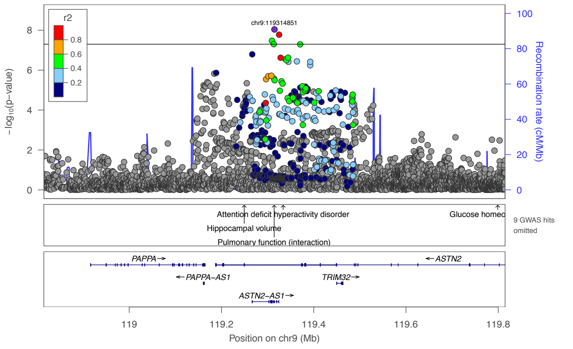

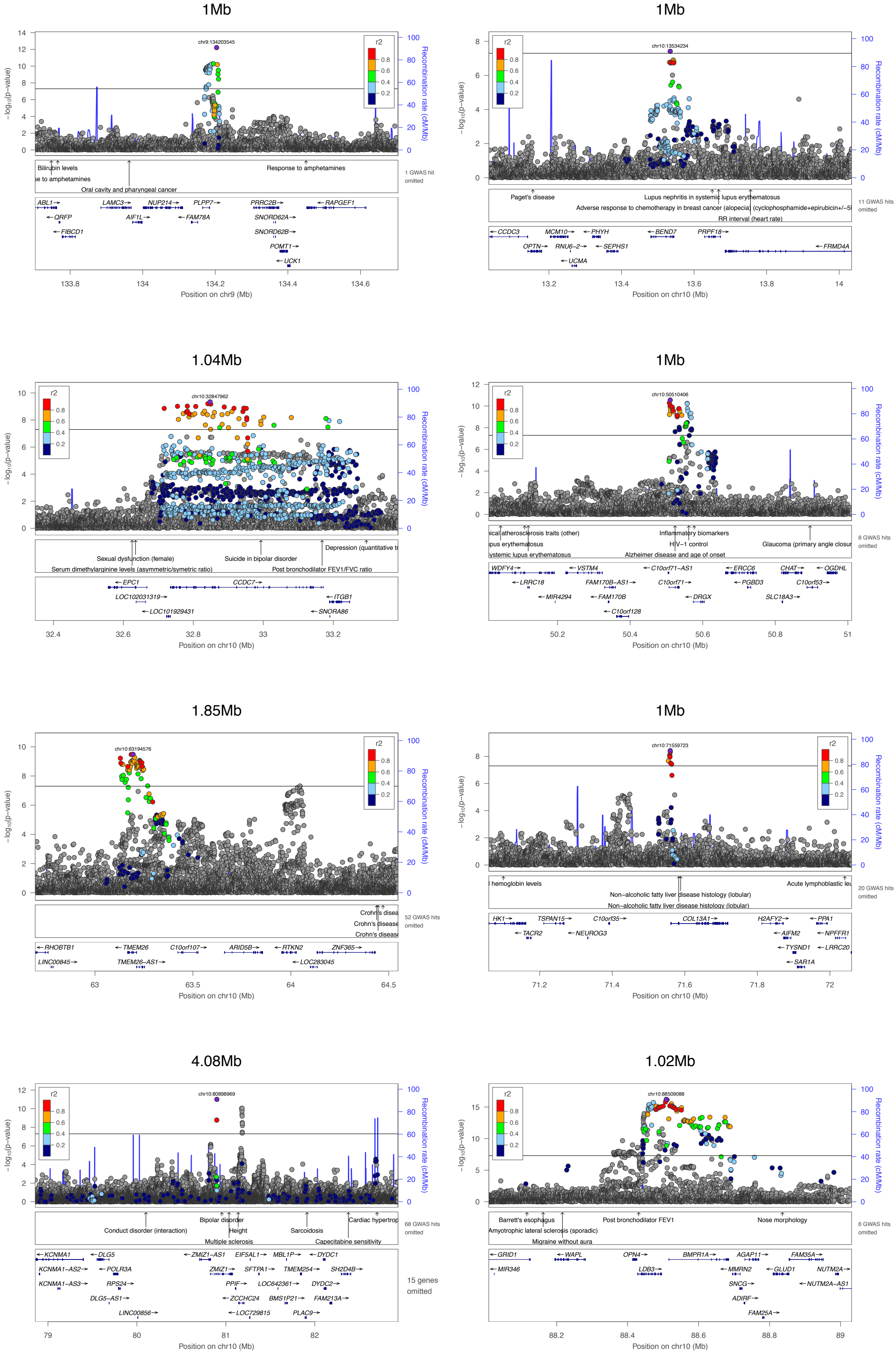
1Mb
chr9:134203545
−log10(p−value)
Recombination rate (cM/Mb)
r2
Bilirubin levels
Response to amphetamines
Oral cavity and pharyngeal cancer
1 GWAS hit omitted
ABL1
LAMC3
NUP214
PLPP7
PRRC2B
RAPGEF1
QRFP
AIF1L
FAM78A
SNORD62A
FIBCD1
SNORD62B
POMT1
UCK1
Position on chr9 (Mb)
1Mb
chr10:13534234
Paget's disease
Lupus nephritis in systemic lupus erythematosus
RR interval (heart rate)
11 GWAS hits omitted
CCDC3
MCM10
PHYH
BEND7
PRPF18
OPTN
RNU6−2
SEPHS1
FRMD4A
UCMA
Position on chr10 (Mb)
1.04Mb
chr10:32847962
Sexual dysfunction (female)
Suicide in bipolar disorder
Serum dimethylarginine levels (asymmetric/symetric ratio)
Post bronchodilator FEV1/FVC ratio
EPC1
CCDC7
LOC102031319
ITGB1
LOC101929431
SNORA86
Position on chr10 (Mb)
1Mb
chr10:50510406
Inflammatory biomarkers
HIV−1 control
Alzheimer disease and age of onset
8 GWAS hits omitted
WDFY4
VSTM4
C10orf71−AS1
ERCC6
CHAT
OGDHL
LRRC18
FAM170B−AS1
C10orf71
PGBD3
C10orf53
MIR4294
FAM170B
DRGX
SLC18A3
C10orf128
Position on chr10 (Mb)
1.85Mb
chr10:63194576
52 GWAS hits omitted
RHOBTB1
TMEM26
C10orf107
ARID5B
RTKN2
ZNF365
LINC00845
TMEM26−AS1
LOC283045
Position on chr10 (Mb)
1Mb
chr10:71559723
Non−alcoholic fatty liver disease histology (lobular)
20 GWAS hits omitted
HK1
TSPAN15
C10orf35
COL13A1
H2AFY2
PPA1
TACR2
NEUROG3
AIFM2
NPFFR1
TYSND1
LRRC20
SAR1A
Position on chr10 (Mb)
4.08Mb
chr10:80898969
Conduct disorder (interaction)
Bipolar disorder
Height
Multiple sclerosis
Sarcoidosis
Capecitabine sensitivity
68 GWAS hits omitted
KCNMA1
DLG5
ZMIZ1−AS1
EIF5AL1
MBL1P
DYDC1
KCNMA1−AS2
POLR3A
ZMIZ1
SFTPA1
TMEM254
SH2D4B
KCNMA1−AS3
RPS24
PPIF
LOC642361
DYDC2
DLG5−AS1
ZCCHC24
BMS1P21
FAM213A
LINC00856
LOC729815
PLAC9
15 genes omitted
Position on chr10 (Mb)
1.02Mb
chr10:88509088
Barrett's esophagus
Amyotrophic lateral sclerosis (sporadic)
Migraine without aura
Post bronchodilator FEV1
Nose morphology
6 GWAS hits omitted
GRID1
WAPL
OPN4
BMPR1A
AGAP11
FAM35A
MIR346
LDB3
MMRN2
GLUD1
NUTM2A
SNCG
NUTM2A−AS1
ADIRF
FAM25A
Position on chr10 (Mb)

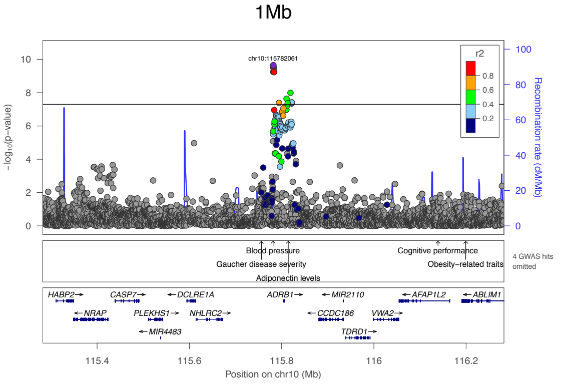
1Mb
chr10:115782061
−log10(p−value)
Recombination rate (cM/Mb)
r2
Blood pressure
Gaucher disease severity
Adiponectin levels
Cognitive performance
Obesity−related traits
4 GWAS hits omitted
HABP2 CASP7 DCLRE1A ADRB1 MIR2110 AFAP1L2 ABLIM1
NRAP PLEKHS1 NHLRC2 CCDC186 VWA2
MIR4483 TDRD1
Position on chr10 (Mb)

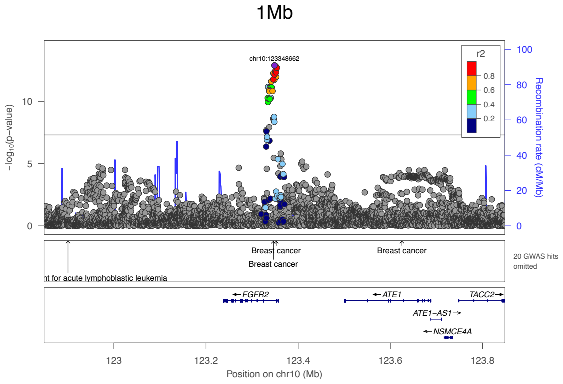
1Mb
chr10:123348662
−log10(p−value)
Recombination rate (cM/Mb)
r2
Breast cancer
Breast cancer
Breast cancer
nt for acute lymphoblastic leukemia
20 GWAS hits omitted
FGFR2 ATE1 TACC2
ATE1−AS1
NSMCE4A
Position on chr10 (Mb)

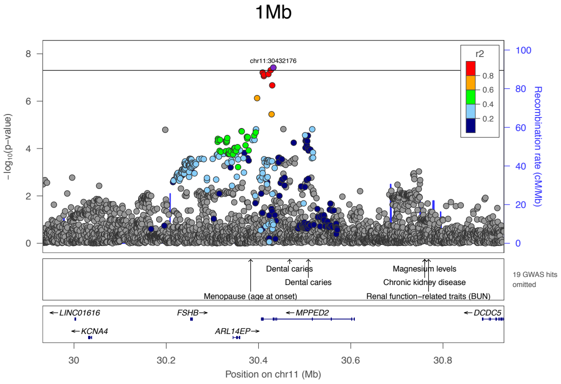
1Mb
chr11:30432176
−log10(p−value)
Recombination rate (cM/Mb)
r2
Dental caries
Dental caries
Menopause (age at onset)
Magnesium levels
Chronic kidney disease
Renal function−related traits (BUN)
19 GWAS hits omitted
LINC01616 FSHB MPPED2 DCDC5
KCNA4 ARL14EP
Position on chr11 (Mb)

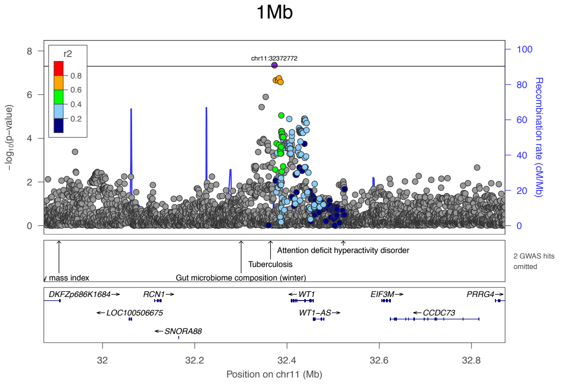
1Mb
chr11:32372772
−log10(p−value)
Recombination rate (cM/Mb)
r2
Attention deficit hyperactivity disorder
Tuberculosis
y mass index
Gut microbiome composition (winter)
2 GWAS hits omitted
DKFZp686K1684 RCN1 WT1 EIF3M PRRG4
LOC100506675 WT1−AS CCDC73
SNORA88
Position on chr11 (Mb)

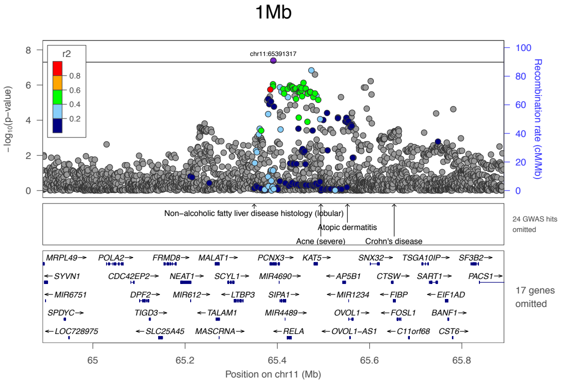
1Mb
chr11:65391317
−log10(p−value)
Recombination rate (cM/Mb)
r2
Non−alcoholic fatty liver disease histology (lobular)
Atopic dermatitis
Acne (severe)
Crohn's disease
24 GWAS hits omitted
MRPL49 POLA2 FRMD8 MALAT1 PCNX3 KAT5 SNX32 TSGA10IP SF3B2
SYVN1 CDC42EP2 NEAT1 SCYL1 MIR4690 AP5B1 CTSW SART1 PACS1
MIR6751 DPF2 MIR612 LTBP3 SIPA1 MIR1234 FIBP EIF1AD
SPDYC TIGD3 TALAM1 MIR4489 OVOL1 FOSL1 BANF1
LOC728975 SLC25A45 MASCRNA RELA OVOL1−AS1 C11orf68 CST6
17 genes omitted
Position on chr11 (Mb)

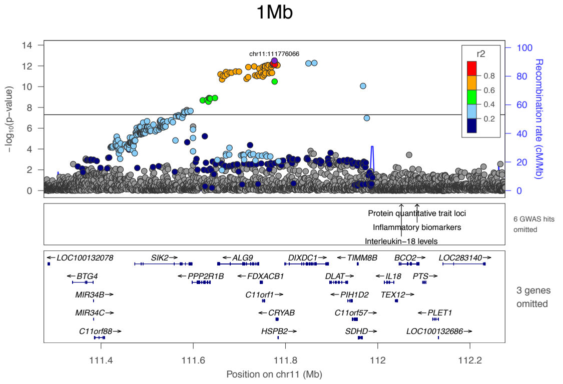
1Mb
chr11:111776066
−log10(p−value)
Recombination rate (cM/Mb)
r2
Protein quantitative trait loci
Inflammatory biomarkers
Interleukin−18 levels
6 GWAS hits omitted
LOC100132078 SIK2 ALG9 DIXDC1 TIMM8B BCO2 LOC283140
BTG4 PPP2R1B FDXACB1 DLAT IL18 PTS
MIR34B C11orf1 PIH1D2 TEX12
MIR34C CRYAB C11orf57 PLET1
C11orf88 HSPB2 SDHD LOC100132686
3 genes omitted
Position on chr11 (Mb)

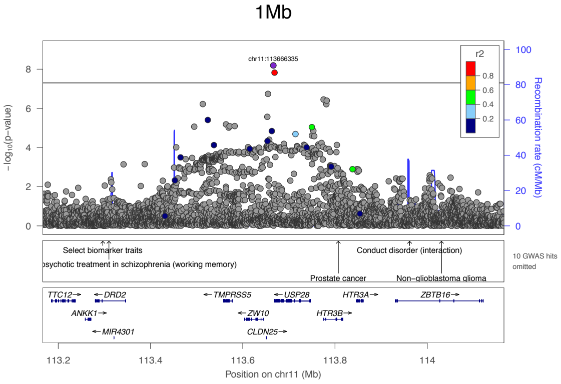
1Mb
chr11:113666335
−log10(p−value)
Recombination rate (cM/Mb)
r2
Select biomarker traits
psychotic treatment in schizophrenia (working memory)
Conduct disorder (interaction)
Prostate cancer
Non−glioblastoma glioma
10 GWAS hits omitted
TTC12 DRD2 TMPRSS5 USP28 HTR3A ZBTB16
ANKK1 ZW10 HTR3B
MIR4301 CLDN25
Position on chr11 (Mb)

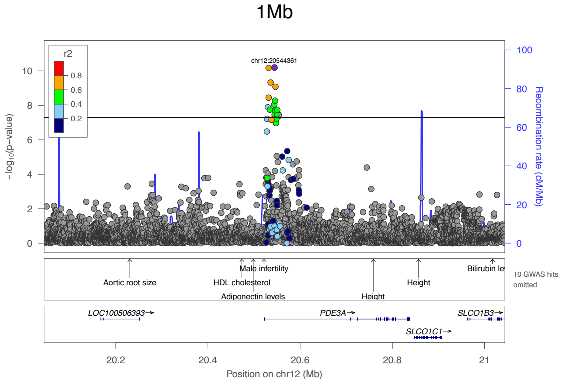
1Mb
chr12:20544361
−log10(p−value)
Recombination rate (cM/Mb)
r2
Male infertility
Bilirubin le
Aortic root size
HDL cholesterol
Height
Adiponectin levels
Height
10 GWAS hits omitted
LOC100506393 PDE3A SLCO1B3
SLCO1C1
Position on chr12 (Mb)

1Mb

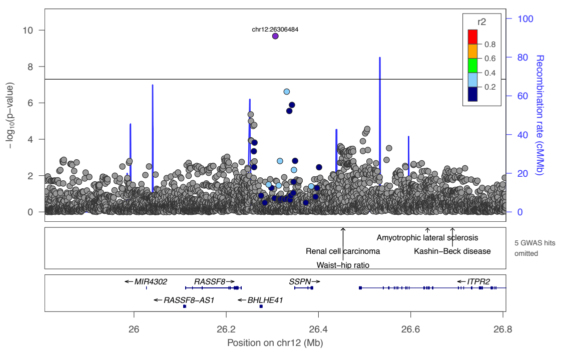

2.16Mb

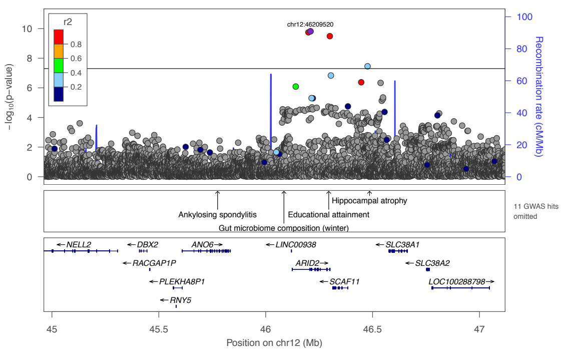

1Mb

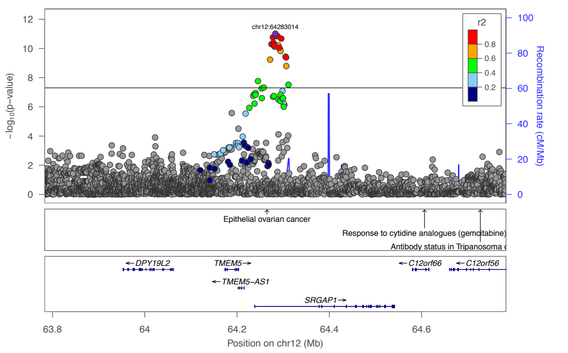

1Mb

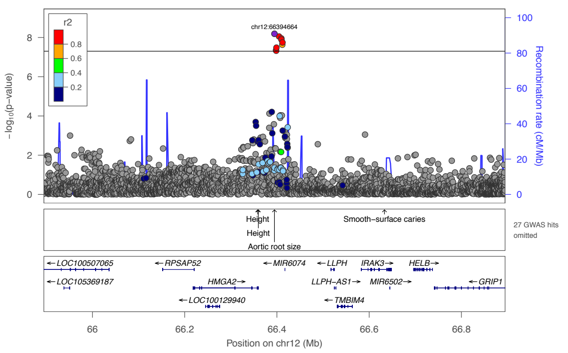

1.65Mb

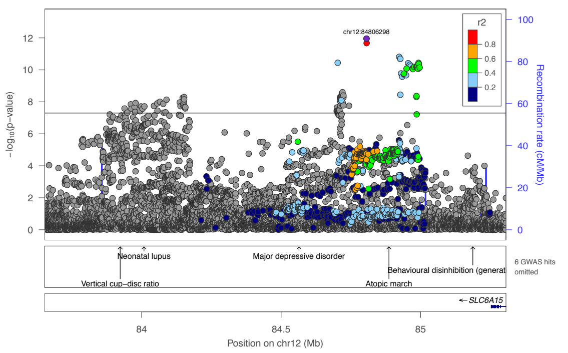

3.44Mb

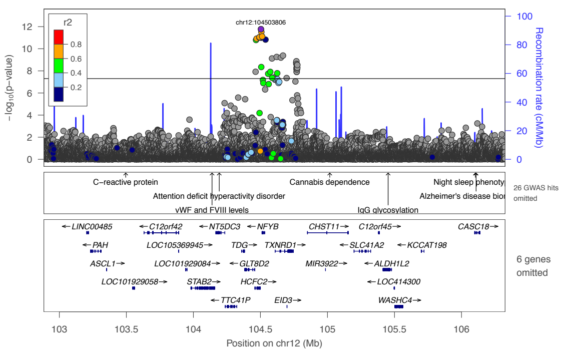

1Mb

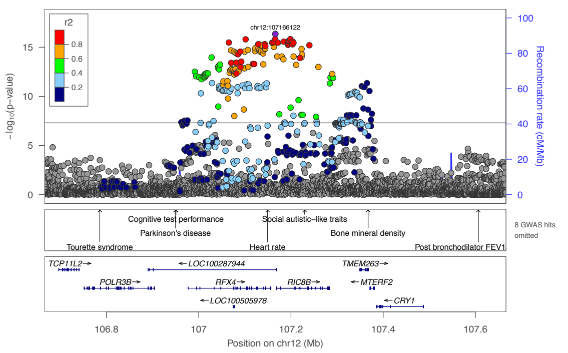

3.95Mb

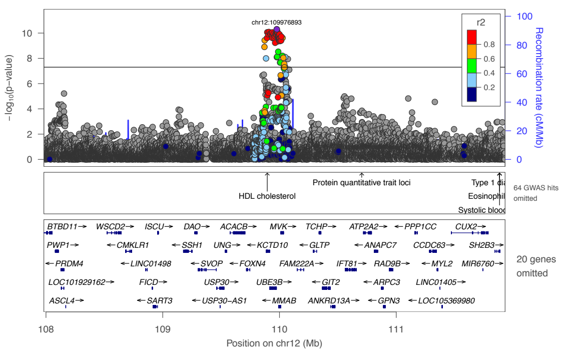

2.1Mb

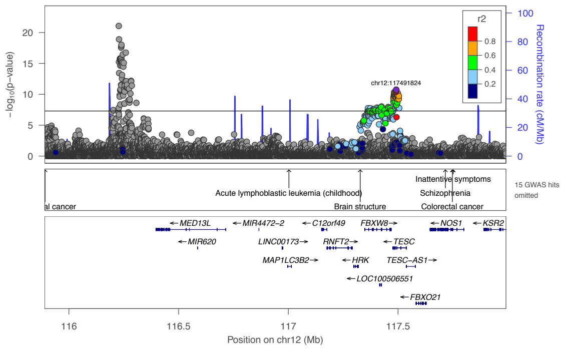

3.88Mb

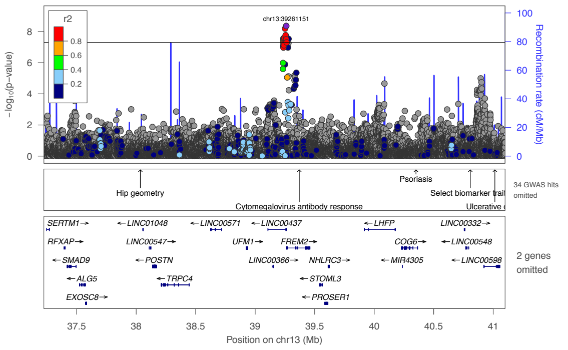

1Mb

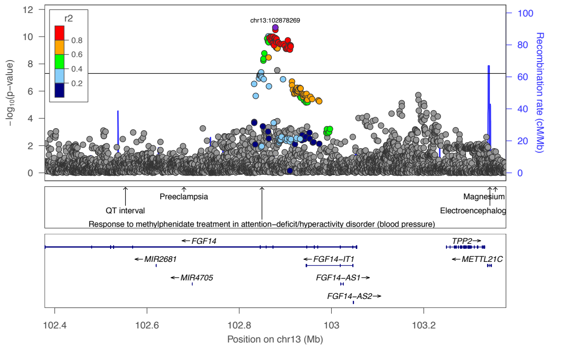

1Mb

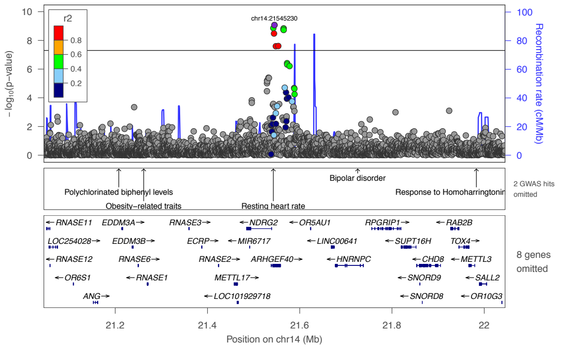

1Mb

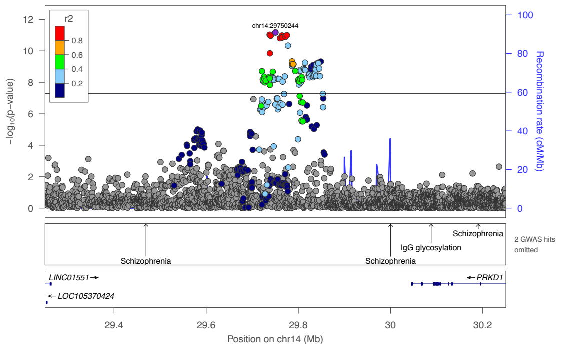

4.09Mb

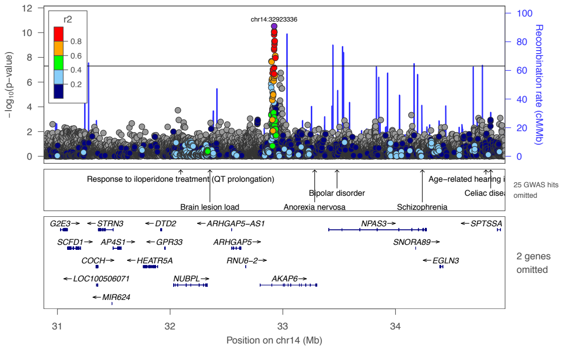

1Mb

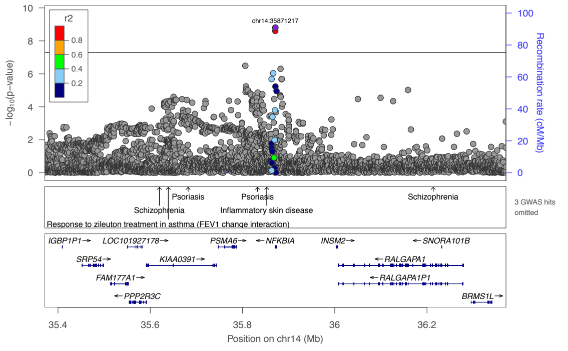

1Mb

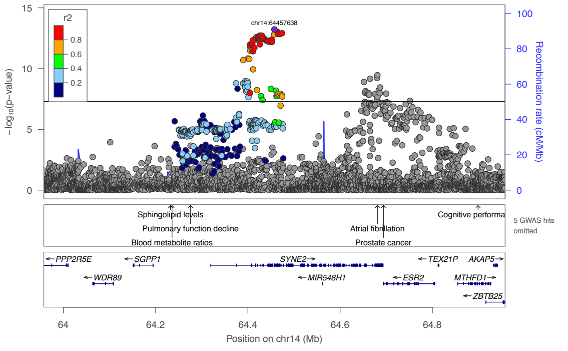

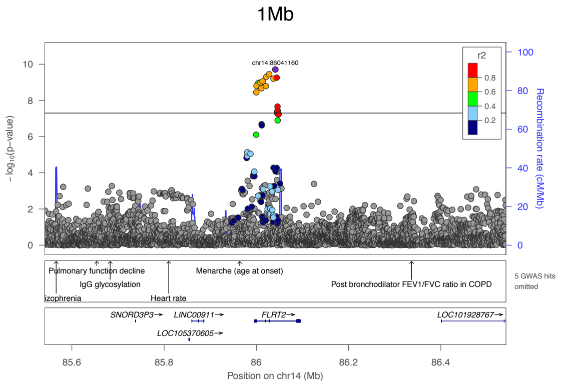
1Mb
chr14:86041160
Recombination rate (cM/Mb)
Pulmonary function decline
IgG glycosylation
Menarche (age at onset)
Post bronchodilator FEV1/FVC ratio in COPD
Heart rate
5 GWAS hits omitted
SNORD3P3
LINC00911
FLRT2
LOC101928767
LOC105370605
Position on chr14 (Mb)

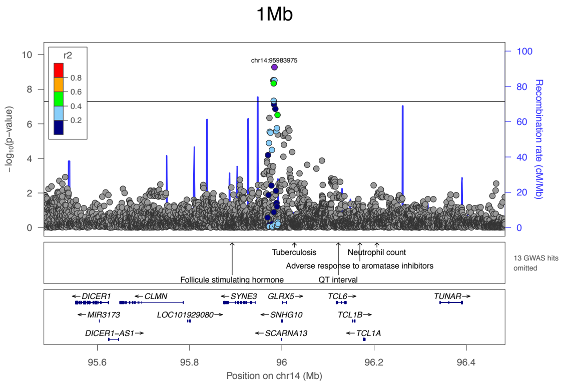
1Mb
chr14:95983975
Recombination rate (cM/Mb)
Tuberculosis
Neutrophil count
Adverse response to aromatase inhibitors
Follicle stimulating hormone
QT interval
13 GWAS hits omitted
DICER1
CLMN
SYNE3
GLRX5
TCL6
TUNAR
MIR3173
LOC101929080
SNHG10
TCL1B
DICER1-AS1
SCARNA13
TCL1A
Position on chr14 (Mb)

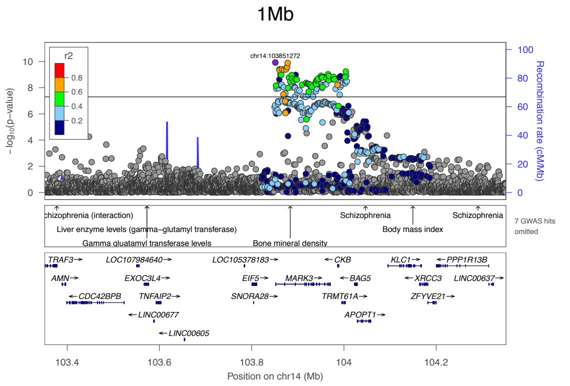
1Mb
chr14:103851272
Recombination rate (cM/Mb)
Schizophrenia
Body mass index
Liver enzyme levels (gamma-glutamyl transferase)
Gamma glutamyl transferase levels
Bone mineral density
7 GWAS hits omitted
TRAF3
LOC107984640
LOC105378183
CKB
KLC1
PPP1R13B
AMN
EXOC3L4
EIF5
MARK3
BAG5
XRCC3
LINC00637
CDC42BPB
TNFAIP2
SNORA28
TRMT61A
ZFYVE21
LINC00677
APOPT1
LINC00605
Position on chr14 (Mb)

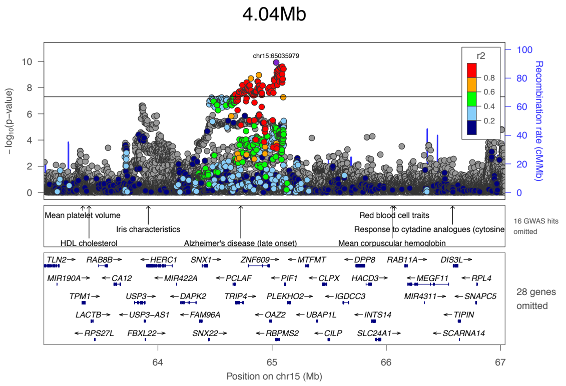
4.04Mb
chr15:65035979
Recombination rate (cM/Mb)
Mean platelet volume
Iris characteristics
HDL cholesterol
Alzheimer's disease (late onset)
Red blood cell traits
Response to cytadine analogues (cytosine
Mean corpuscular hemoglobin
16 GWAS hits omitted
TLN2
RAB8B
HERC1
SNX1
ZNF609
MTFMT
DPP8
RAB11A
DIS3L
MIR190A
CA12
MIR422A
PCLAF
PIF1
CLPX
HACD3
MEGF11
RPL4
TPM1
USP3
DAPK2
TRIP4
PLEKHO2
IGDCC3
MIR4311
SNAPC5
LACTB
USP3-AS1
FAM96A
OAZ2
UBAP1L
INTS14
TIPIN
RPS27L
FBXL22
SNX22
RBPMS2
CILP
SLC24A1
SCARNA14
28 genes omitted
Position on chr15 (Mb)

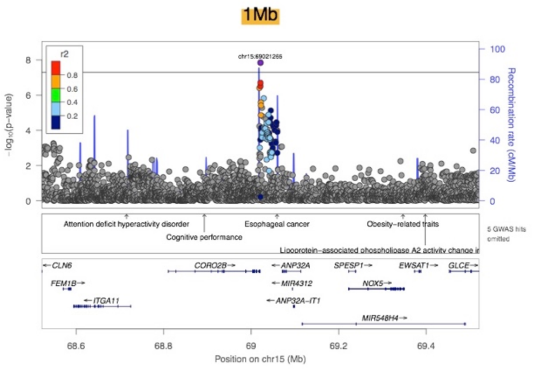
1Mb
chr15:69021269
Recombination rate (cM/Mb)
Attention deficit hyperactivity disorder
Esophageal cancer
Obesity-related traits
Cognitive performance
Lipoprotein-associated phospholipase A2 activity change in
5 GWAS hits omitted
CLN6
CORO2B
ANP32A
SPESP1
EWSAT1
GLCE
FEM1B
MIR4312
NOX5
ITGA11
ANP32A-IT1
MIR548H4
Position on chr15 (Mb)

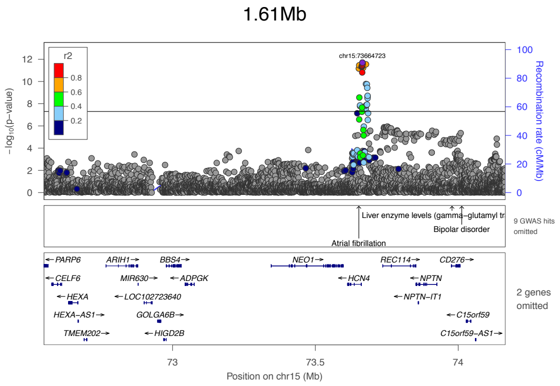
1.61Mb
chr15:73664723
Recombination rate (cM/Mb)
Liver enzyme levels (gamma-glutamyl tr
Bipolar disorder
Atrial fibrillation
9 GWAS hits omitted
PARP6
ARIH1
BBS4
NEO1
REC114
CD276
CELF6
MIR630
ADPGK
HCN4
NPTN
HEXA
LOC102723640
NPTN-IT1
HEXA-AS1
GOLGA6B
C15orf59
TMEM202
HIGD2B
C15orf59-AS1
2 genes omitted
Position on chr15 (Mb)

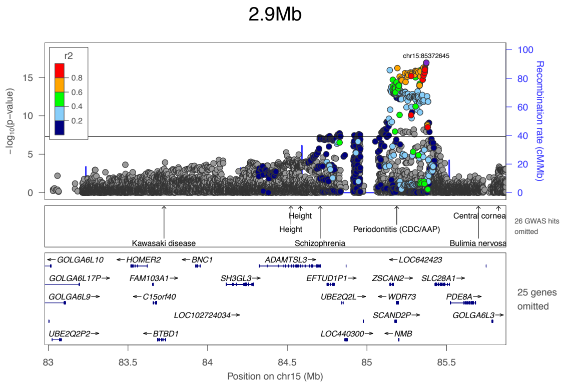
2.9Mb
chr15:85372645
Recombination rate (cM/Mb)
Height
Central cornea
Periodontitis (CDC/AAP)
Kawasaki disease
Schizophrenia
Bulimia nervosa
26 GWAS hits omitted
GOLGA6L10
HOMER2
BNC1
ADAMTSL3
LOC642423
GOLGA6L17P
FAM103A1
SH3GL3
EFTUD1P1
ZSCAN2
SLC28A1
GOLGA6L9
C15orf40
UBE2Q2L
WDR73
PDE8A
LOC102724034
SCAND2P
GOLGA6L3
UBE2Q2P2
BTBD1
LOC440300
NMB
25 genes omitted
Position on chr15 (Mb)

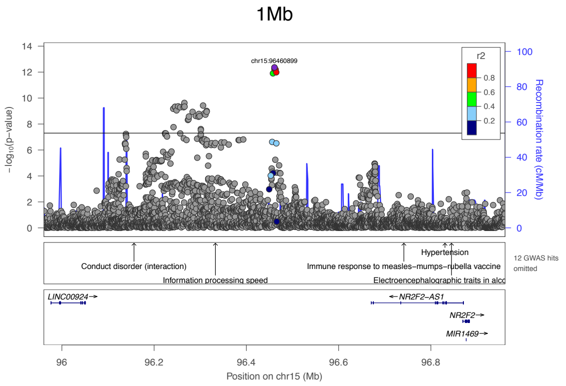
1Mb
chr15:96460899
Recombination rate (cM/Mb)
Conduct disorder (interaction)
Information processing speed
Hypertension
Immune response to measles-mumps-rubella vaccine
Electroencephalographic traits in alco
12 GWAS hits omitted
LINC00924
NR2F2-AS1
NR2F2
MIR1469
Position on chr15 (Mb)

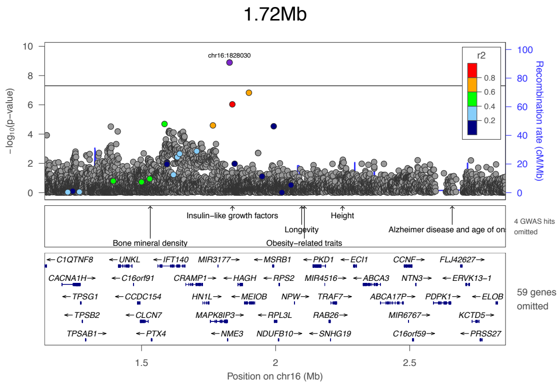
1.72Mb
chr16:1828030
Insulin−like growth factors
Height
Longevity
Alzheimer disease and age of on
Bone mineral density
Obesity−related traits
4 GWAS hits omitted
59 genes omitted
Position on chr16 (Mb)

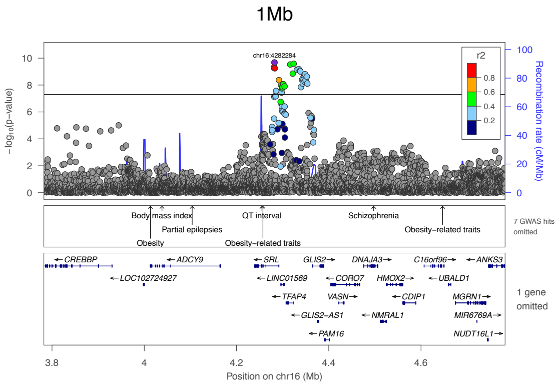
1Mb
chr16:4262284
Body mass index
QT interval
Schizophrenia
Partial epilepsies
Obesity−related traits
Obesity
7 GWAS hits omitted
1 gene omitted
Position on chr16 (Mb)

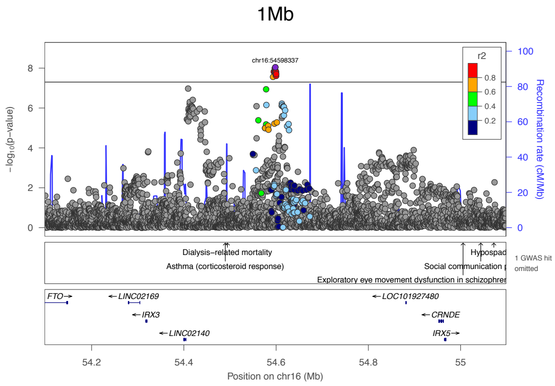
1Mb
chr16:54598337
Dialysis−related mortality
Asthma (corticosteroid response)
Social communication
Exploratory eye movement dysfunction in schizophrenia
1 GWAS hit omitted
Position on chr16 (Mb)

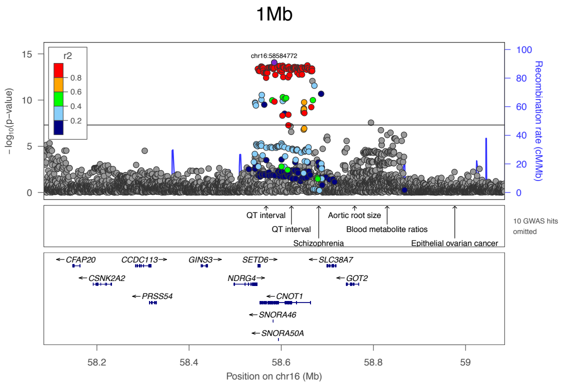
1Mb
chr16:58584772
QT interval
Aortic root size
Blood metabolite ratios
Schizophrenia
Epithelial ovarian cancer
10 GWAS hits omitted
Position on chr16 (Mb)

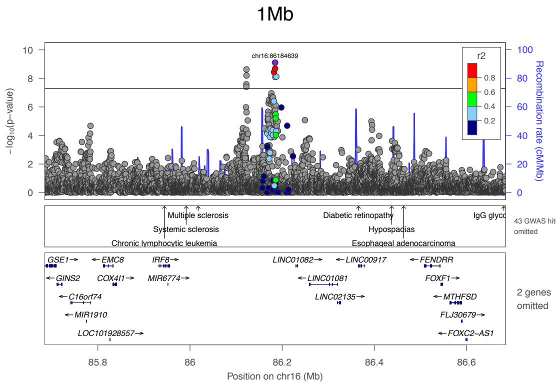
1Mb
chr16:86184639
Multiple sclerosis
Systemic sclerosis
Chronic lymphocytic leukemia
Diabetic retinopathy
Hypospadias
Esophageal adenocarcinoma
43 GWAS hits omitted
2 genes omitted
Position on chr16 (Mb)

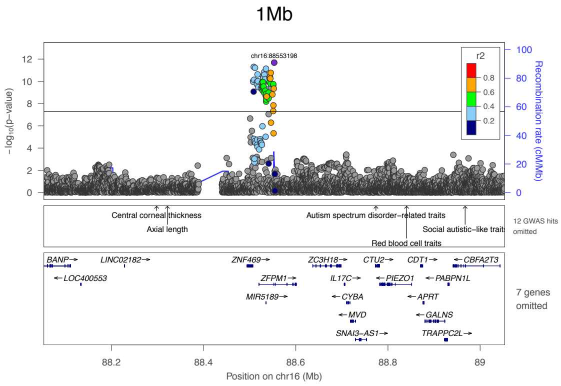
1Mb
chr16:88553198
Central corneal thickness
Axial length
Autism spectrum disorder−related traits
Social autistic−like traits
Red blood cell traits
12 GWAS hits omitted
7 genes omitted
Position on chr16 (Mb)

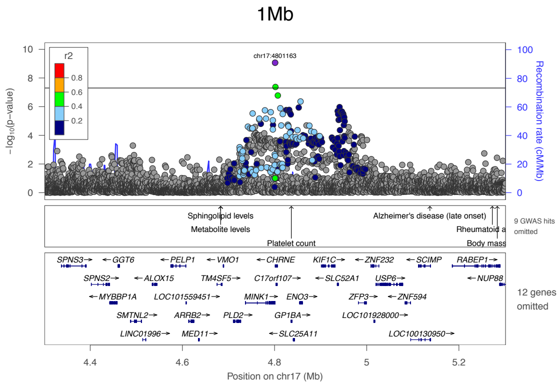
1Mb
chr17:4801163
Sphingolipid levels
Metabolite levels
Platelet count
Alzheimer's disease (late onset)
Body mass
9 GWAS hits omitted
12 genes omitted
Position on chr17 (Mb)

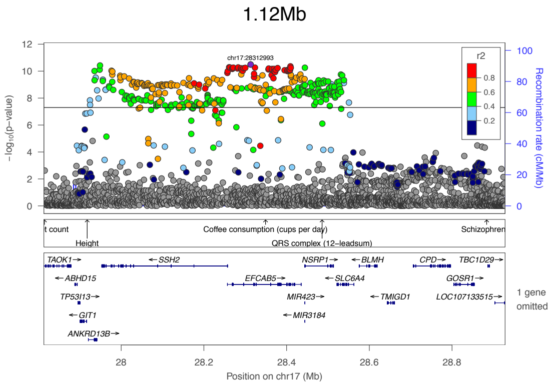
1.12Mb
chr17:28312993
Height
Coffee consumption (cups per day)
QRS complex (12−leadsum)
1 gene omitted
Position on chr17 (Mb)

2.14Mb

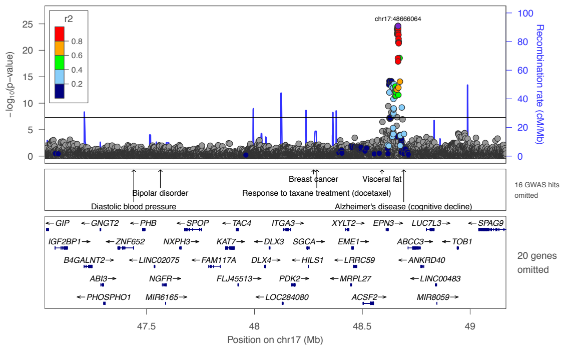

1Mb

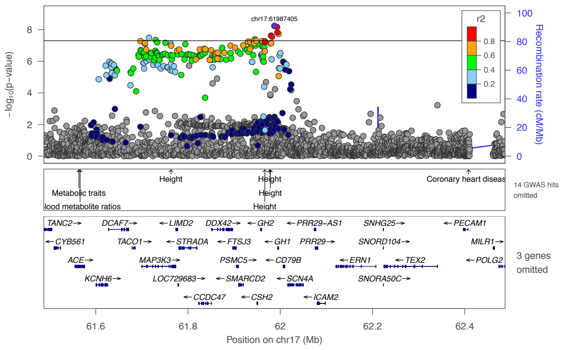

1Mb

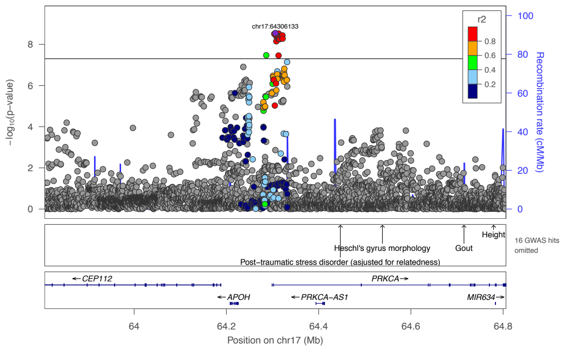

1.75Mb

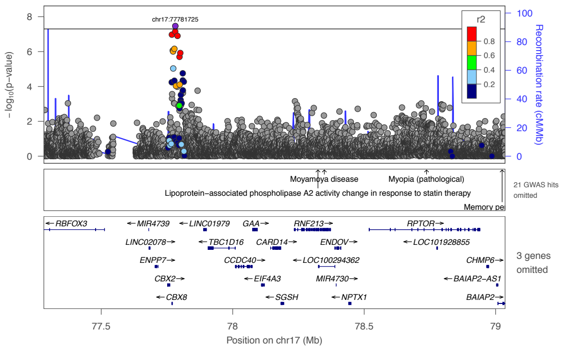

1.48Mb

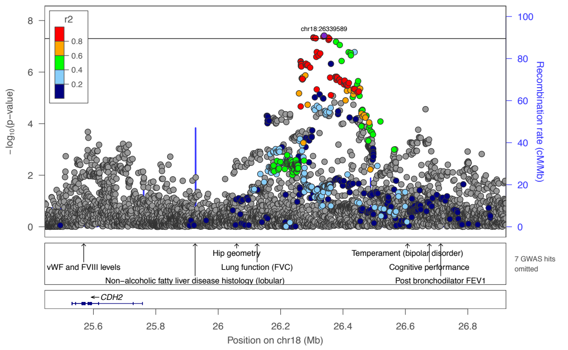

1.08Mb

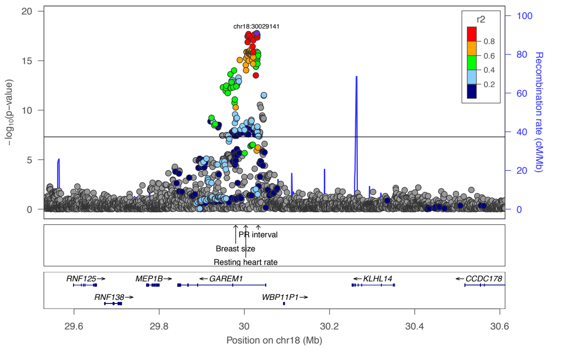

1Mb

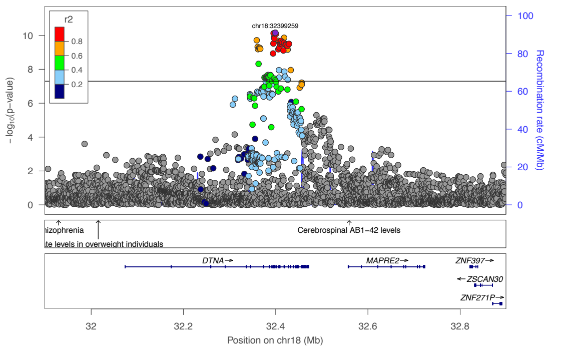

1Mb

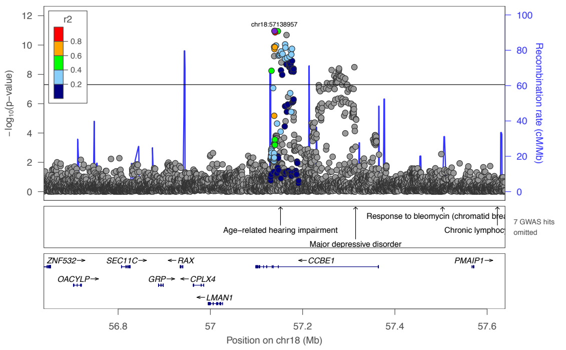

2.86Mb

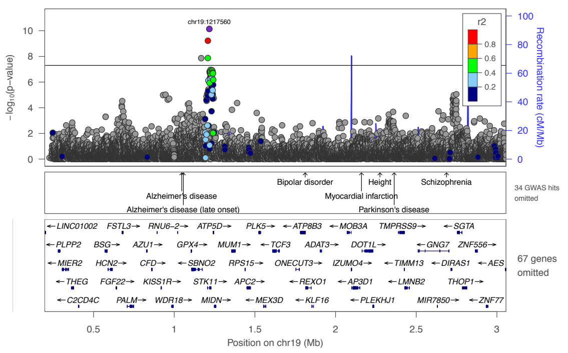

1Mb

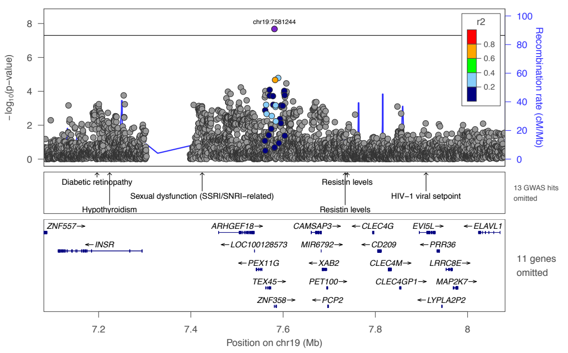

1Mb

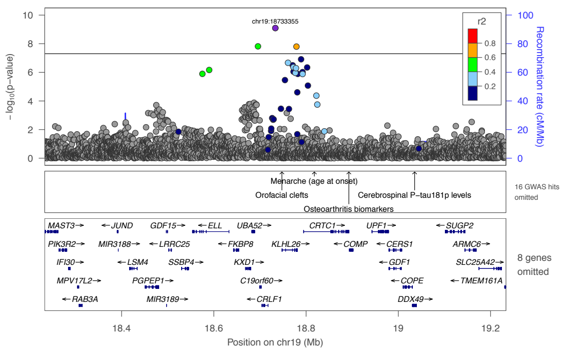

1Mb

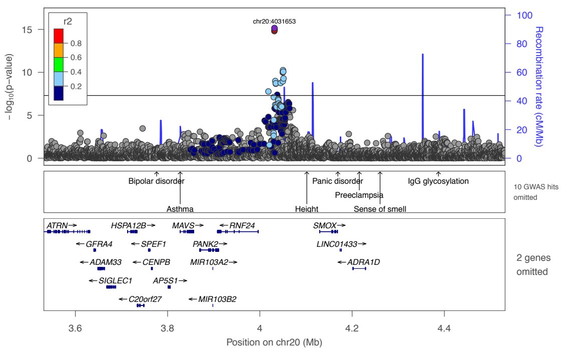

1Mb

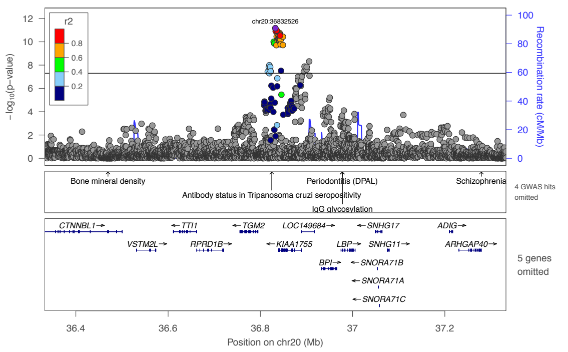

1.41Mb

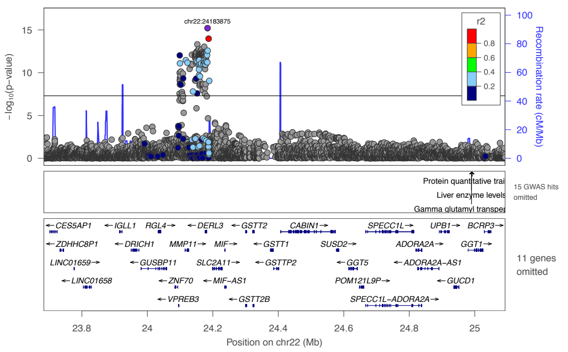

1.25Mb

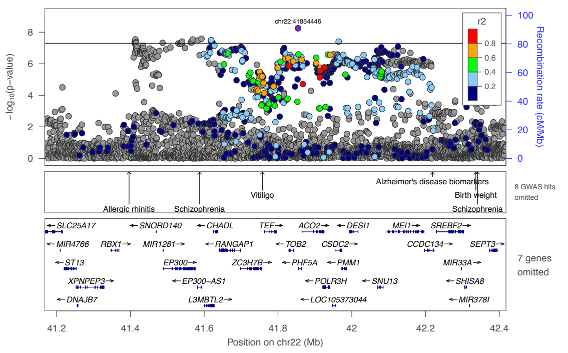

Previously not reported loci from European-only meta-analysis

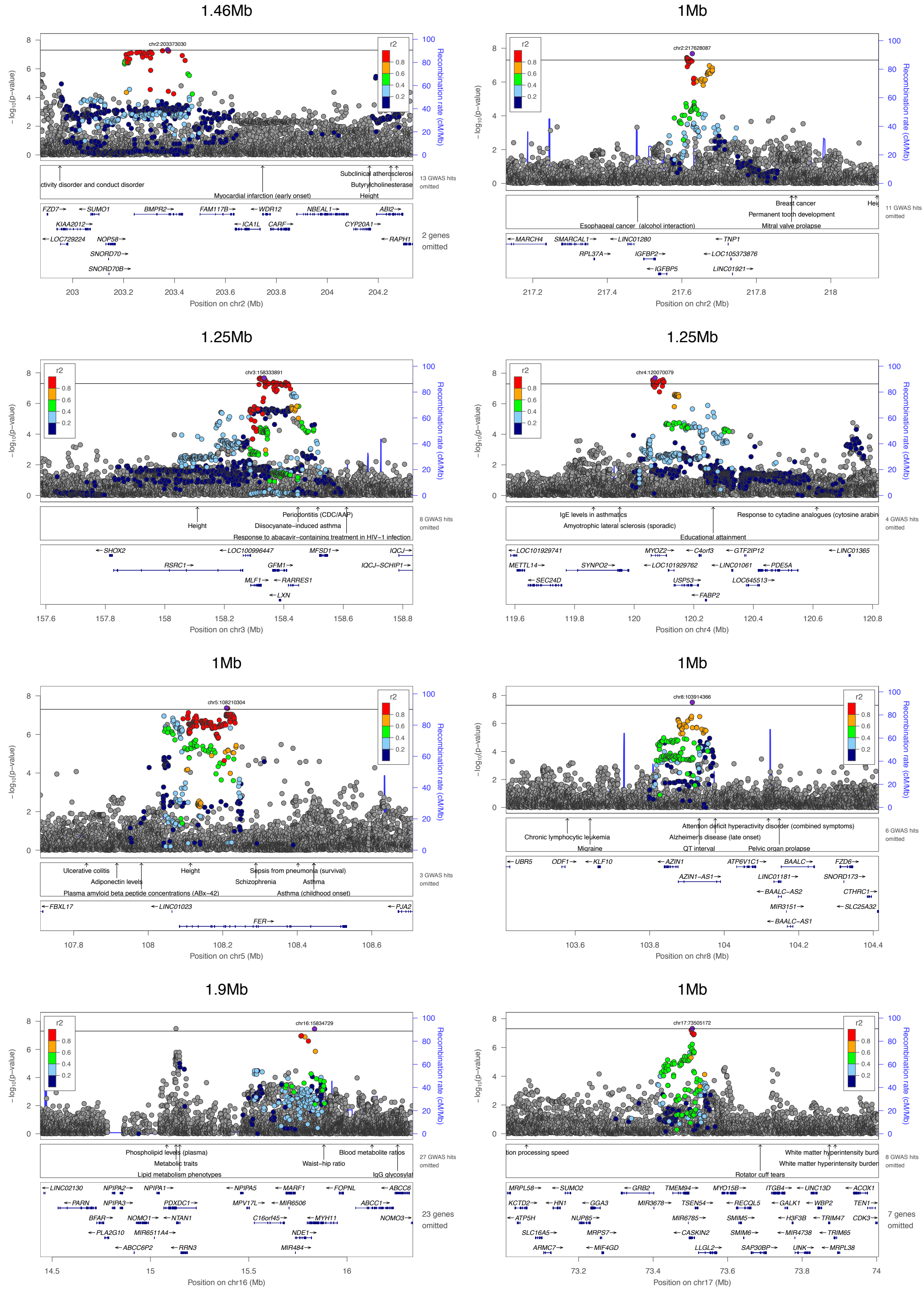

The title represents the length of the region in each region association plot, and the title is highlighted in yellow when the top variant at that locus is an indel. P values are plotted on the $-\log_{10}$ scale for all variants present in at least 60% of the maximum sample size from fixed-effects meta-analysis (N=293,051 for multi-ancestry analysis and N=271,570 for European-ancestry analysis). Pairwise LD were estimated using the 1000G phase 3 all ancestry samples (N = 2,504) or European only (N = 503), except rs4868384. LD for rs4868384 was estimated using the 1000G phase 1 all ancestry due to unavailability in phase 3. Details of LD and window calculation are described in **Supplementary Method.**

Supplementary Figure 4 Correlation of association statistics for the lead variants at the 141 previously not reported loci identified by the multi-ancestry meta-analysis and for the 8 genome-wide significant loci in the European ancestry meta-analysis of absolute PR interval.

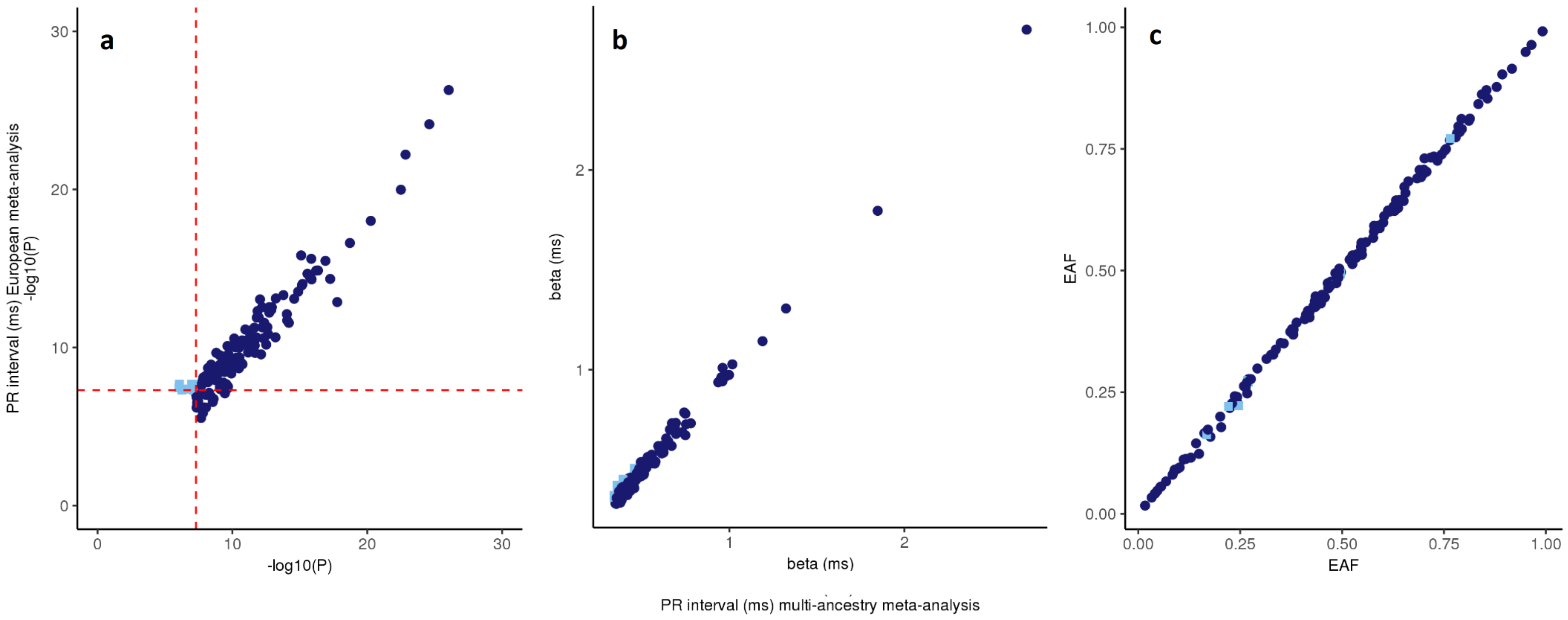

Correlations are presented for P values (a), betas (b), and effect allele frequencies (EAF, c). Lead variants discovered from the multi-ancestry (N = 141) meta-analysis are shown in dark blue circles. The 8 loci that reached genome-wide significance in the European meta-analysis and were borderline genome-wide significant in the multi-ancestry meta-analysis are shown in light blue squares. P values are plotted on the $-\log_{10}$ scale. Red dashed lines indicate the genome-wide significance level ($P = 5 \times 10^{-8}$). Sample sizes are 293,051 for multi-ancestry analysis and 271,570 for European-ancestry analysis.

Supplementary Figure 5 Quantile-Quantile plots of chromosome X variant results from meta-analyses of absolute PR interval.

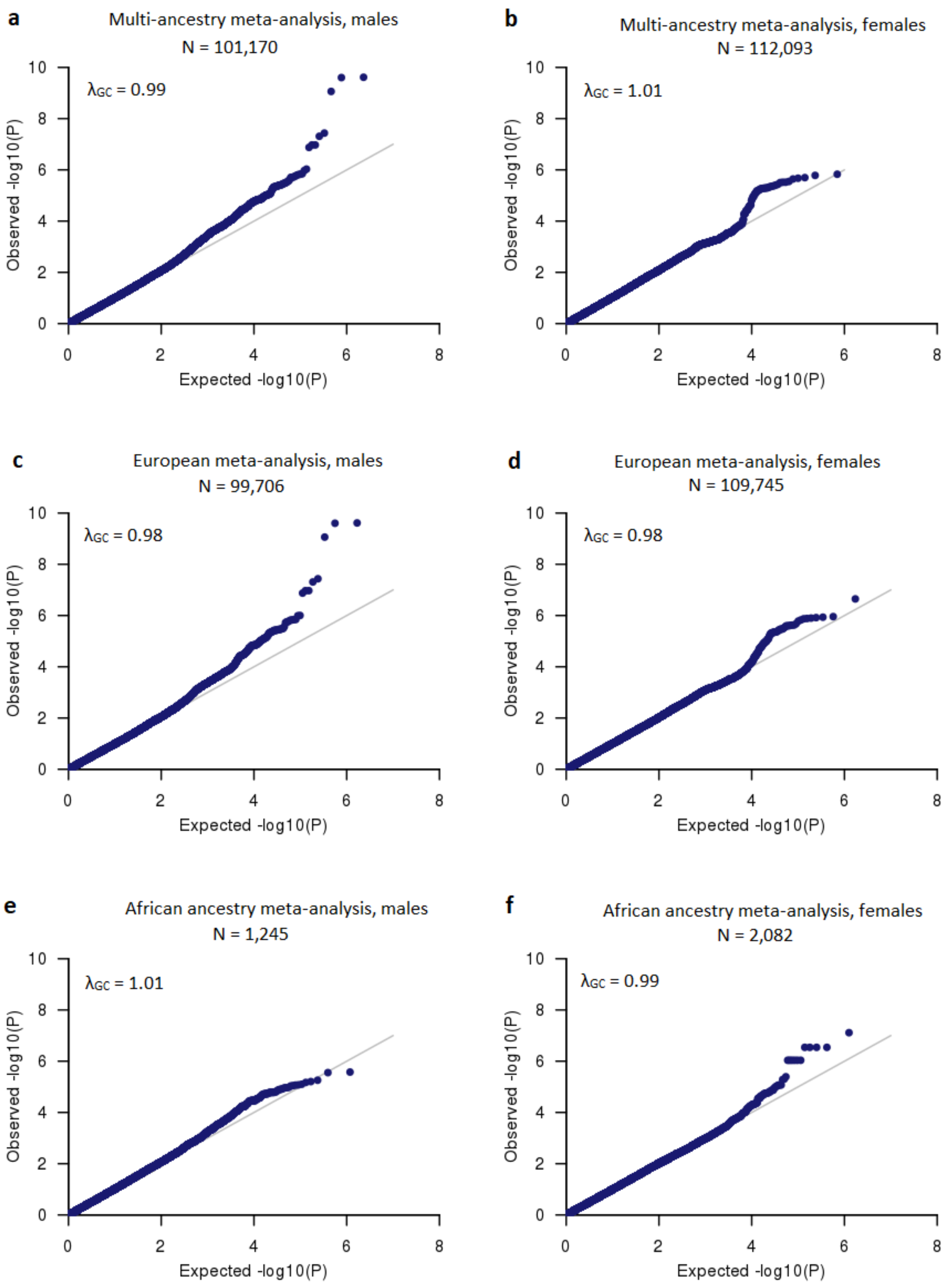

P values from multi-ancestry (a, b), European ancestry (c, d), and African (e, f) ancestry meta-analyses of chromosome X variants for absolute PR interval (ms) are plotted on the -log_{10} scale for all variants. Sample size of each analysis is provided at the top of each panel. Chromosome X meta-analyses were performed separately for males (a, c, e) and females (b, d, f). The genomic inflation factors (λ_{GC}) are provided at the top left of each plot.

Supplementary Figure 6 Correlation of P values across the meta-analyses of absolute and rank-based inverse normal transformed residuals of PR interval.

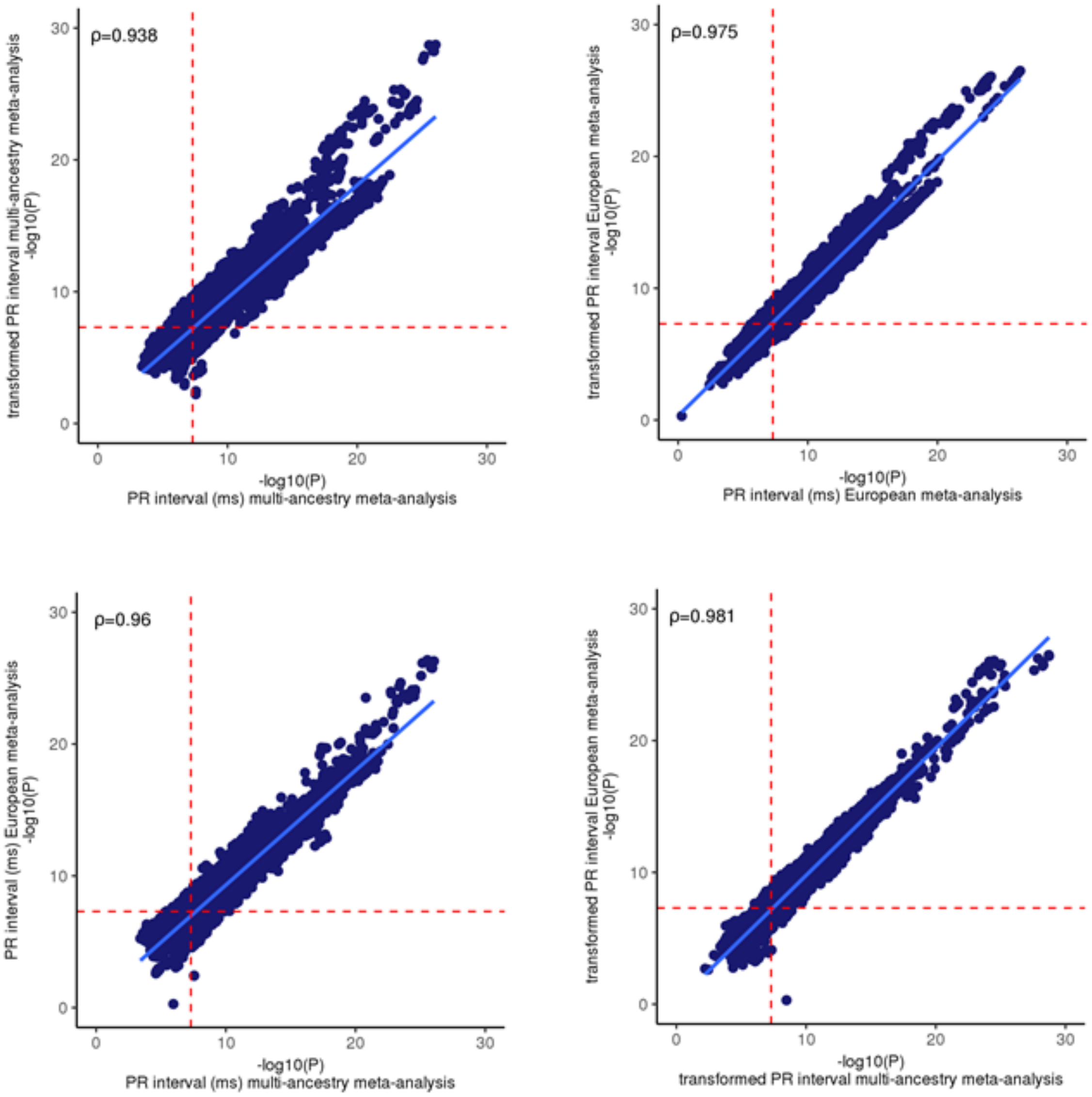

Correlations across of P values across multi-ancestry and European ancestry meta-analyses of absolute PR interval and rank-based inverse normal transformed PR interval are presented. Sample sizes are 293,051 for multi-ancestry analysis and 271,570 for European-ancestry analysis. P values are plotted for variants with $P < 10^{-6}$ in any of the four meta-analyses outside previous reported loci (N = 16,267) that were available in at least 60% of the maximum sample size of each meta-analysis on the $-\log_{10}$ scale. The blue lines show the corresponding regression

lines. Red dashed lines indicate the genome-wide significance level ($P = 5 \times 10^{-8}$). Correlation coefficients (ρ) between the two P values are provided on the top left of each plot.

Supplementary Figure 7 Genome-wide significant loci ($P < 5 \times 10^{-8}$) across meta-analyses of absolute and rank-based inverse normal transformed PR interval.

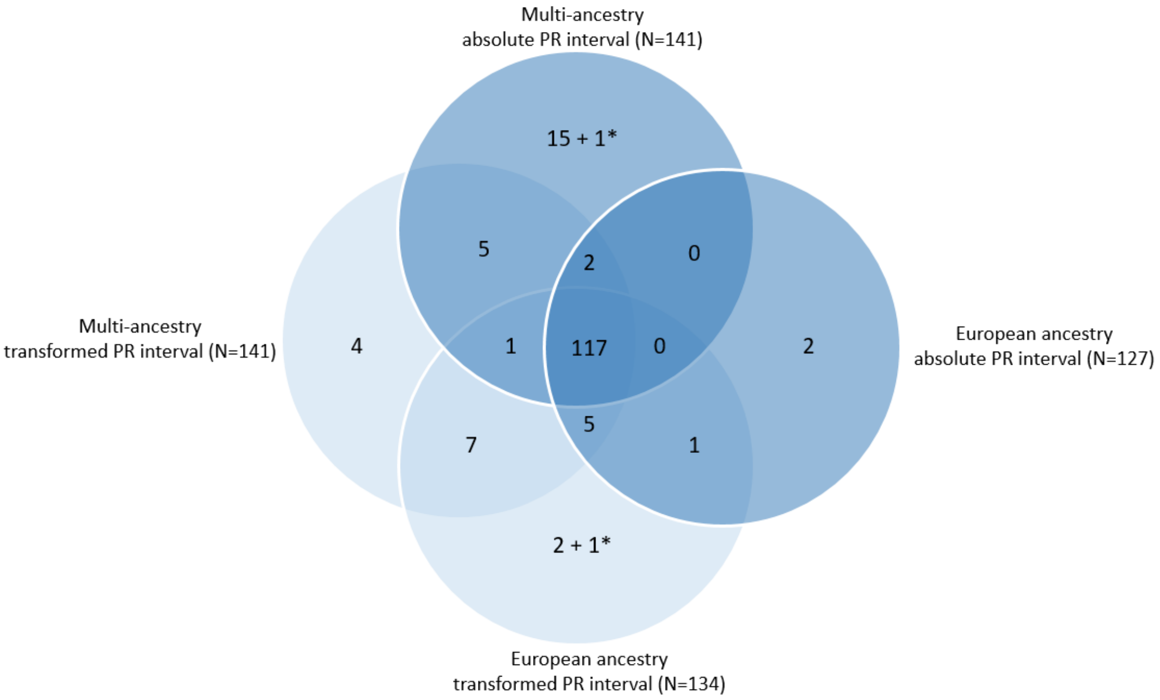

The Venn diagram shows overlap of genome-wide significant loci ($P = 5 \times 10^{-8}$) across multi-ancestry and European ancestry meta-analyses of absolute (darker blue circles; N = 293,051 and N = 271,570 respectively) and rank-based inverse normal transformed PR interval (light blue circles; N = 282,128 and N = 271,570 respectively). A total of 130 out of the 149 newly reported loci discovered by the multi-ancestry and the European ancestry meta-analyses of absolute PR interval reached also genome-wide significance in the meta-analyses of rank-based inverse normal transformed PR interval.

* One locus reached genome-wide significance in multi-ancestry meta-analysis of absolute PR interval and in European meta-analysis of rank-based inverse normal transformed PR interval.

Supplementary Figure 8 Volcano plot of transcriptome-wide analysis for PR interval duration.

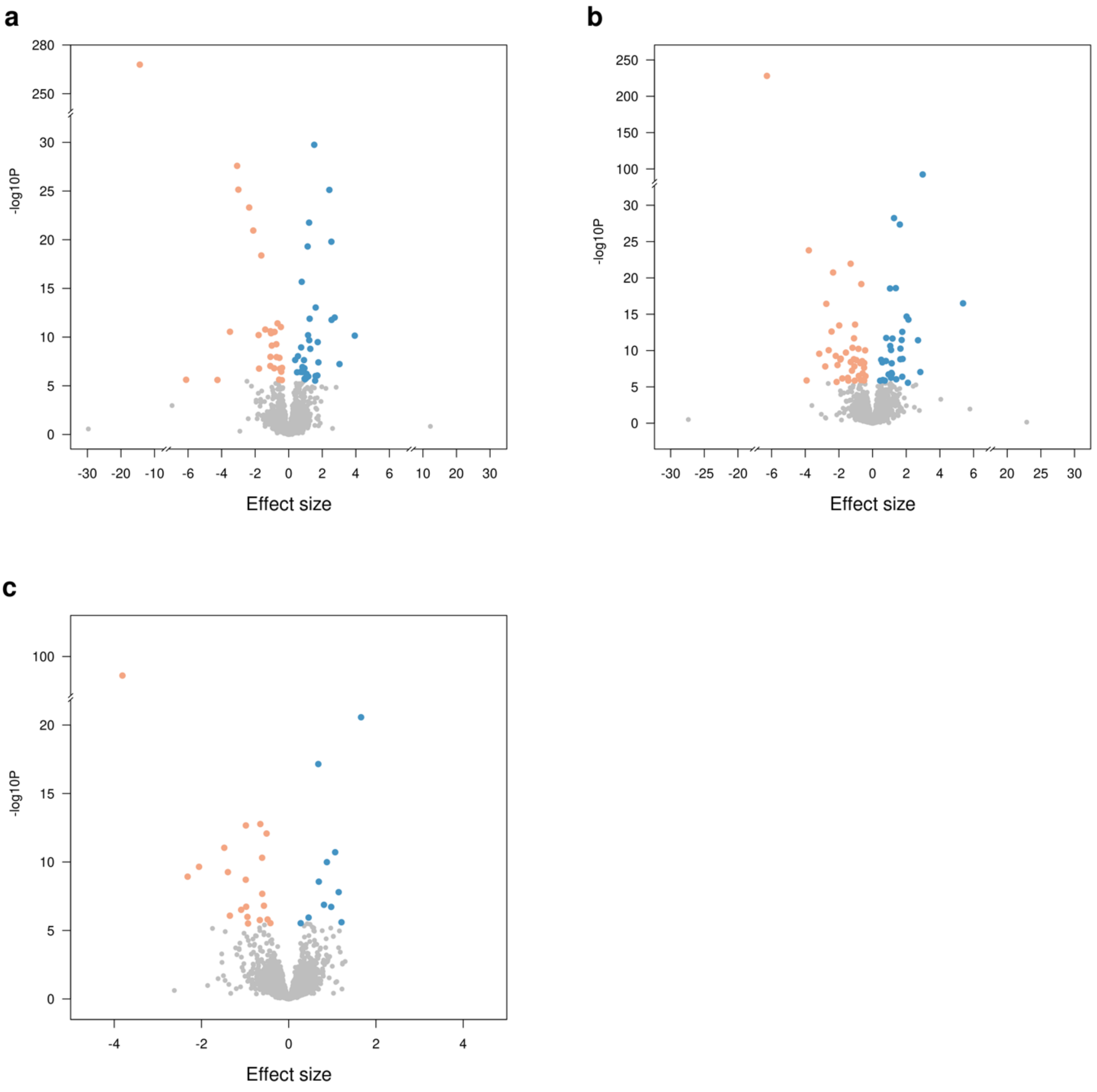

The plots show the results from predicted gene expression analysis in left ventricle (a), right atrial appendage (b), and spleen (c) tissues from GTEx version 7. Analysis was performed with S-PrediXcan using the European meta-analysis summary level results. Sample sizes are 233, 231 and 119 for left ventricle, right atrium appendage, and spleen tissues from GTEx, respectively. For European meta-analysis, sample size is 271,570. The x-axis shows the effect size for associations of predicted gene expression and PR interval duration for each gene. The

y-axis shows the $-\log_{10}(P)$ for the associations per gene. Each plotted point represents the association results of a single gene. The highlighted genes are significant after Bonferroni correction for all tested genes at the three tissues with a $P < 3.1 \times 10^{-6}$ (=0.05/(5,977+5,366+4,598)). Genes with positive effect (blue) showed an association of increased predicted gene expression with PR interval duration. Genes with negative effect (orange) showed an association of decreased predicted gene expression with PR interval duration.

Supplementary Figure 9 Enrichment of PR interval variants in DNAse I Hypersensitive sites.

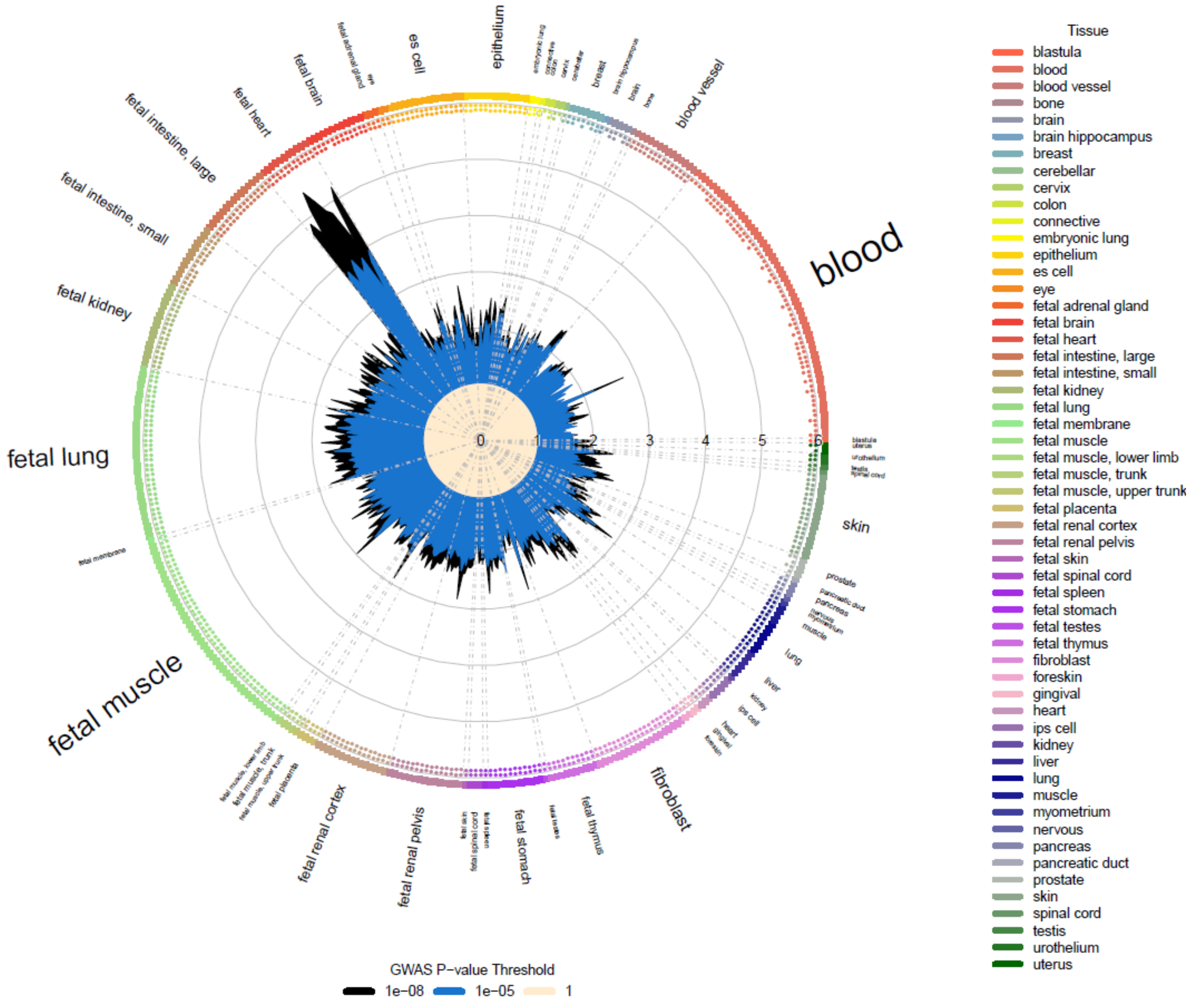

Radial plot shows odds ratio (OR) values at two GWAS P-value thresholds (T), ($T<10^{-5}$ and $T<10^{-8}$, shown by inner colors and bottom legend) for all ENCODE[24] and Roadmap Epigenomics[25] DHS cell lines, sorted by tissue on the outer circle. Small dots on the outer side of the plot show significant enrichment (if present) at $T<10^{-5}$ (outermost) to $T<10^{-8}$ (innermost) after multiple-testing correction for the number of effective annotations and are colored with respect to the tissue cell type tested (font size of tissue labels reflects the number of cell types from that tissue).

Supplementary Figure 10 Association of previously not reported PR interval loci with other GWAS traits.

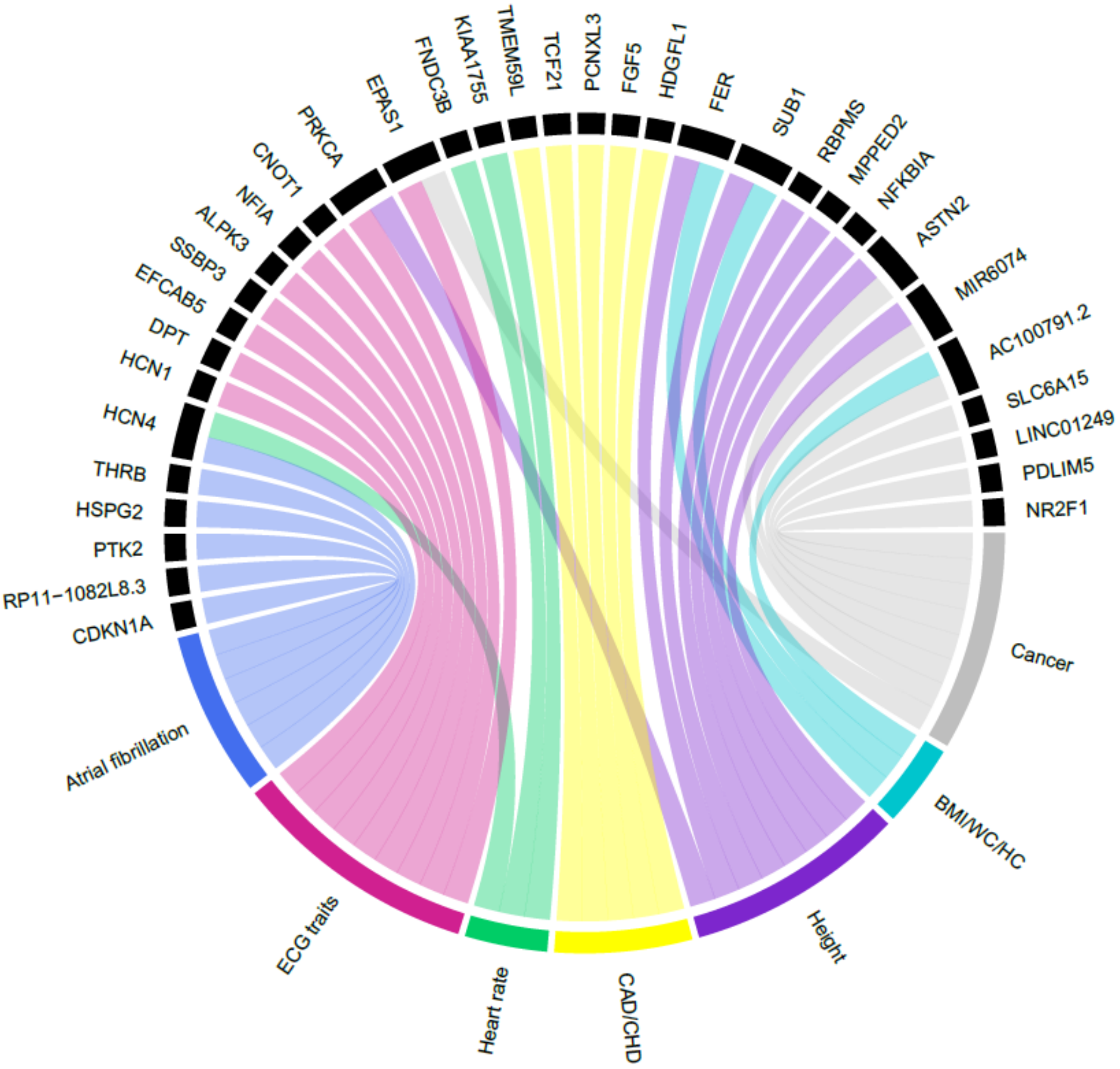

The chord plot shows results for genome-wide significant ($P < 5\times10^{-8}$) associations with other GWAS traits which were extracted from PhenoScanner v2[26] database for the lead and conditionally independent variants at the 141 previously not reported loci in the multi-ancestry meta-analysis (Supplementary Data 3) and for the 8 genome-wide significant loci in the European ancestry meta-analysis that were borderline genome-wide significant in the multi-ancestry meta-analysis (Supplementary Data 6) as well as their proxies ($r^2>0.8$). Traits were

grouped into broader categories, and results are presented only for traits associated with three or more PR interval loci. Detailed PhenoScanner GWAS results are presented in Supplementary Data 17. For atrial fibrillation, we curated a list of all overlapping loci (r^2>0.7) with PR interval including the recent findings from two GWASs[2,27] not included in PhenoScanner v2 database.

ECG, electrocardiogram; CAD: coronary artery disease; CHD: coronary heart disease; BMI, body mass index; WC, waist circumference; HC: hip circumference.

Supplementary Figure 11 Associations between lead PR interval variants (205 single nucleotide polymorphisms) with atrial fibrillation (AF) risk from recently published AF GWASs

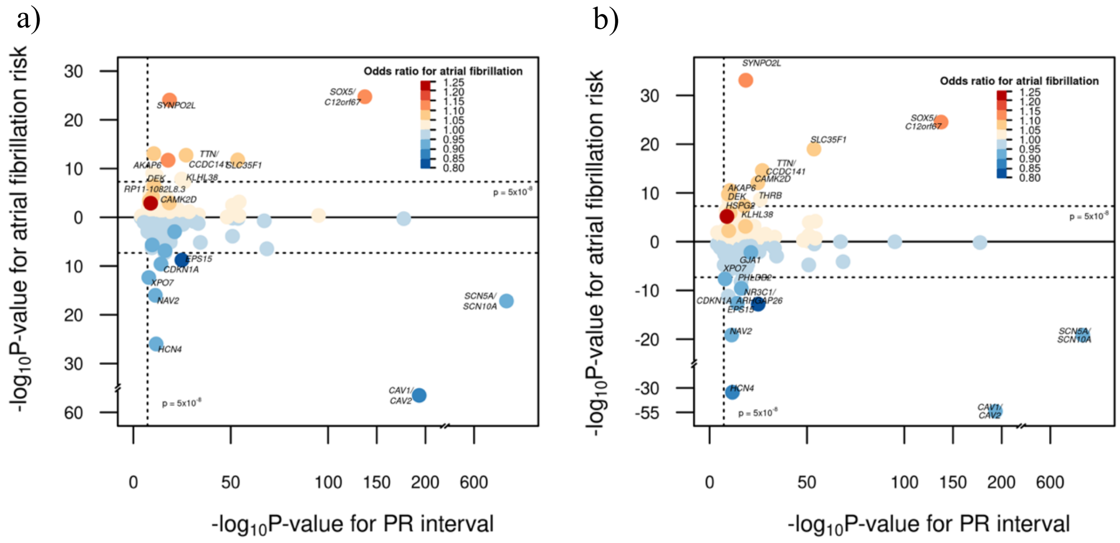

Panel (a) is the look-up results from Roselli, el al.[2], and panel (b) is the look-up results from Nielsen, et al.[27] The X axis refers to -log$_{10}$ P-value for PR interval from either multi-ancestry meta-analysis (N = 293,051) or European ancestry meta-analysis (N = 271,570). Y axis refers to -log$_{10}$ P-value for AF risk from prior AF GWAS.[2,27] Red/orange color indicates the same direction of effect for PR interval and AF risk, while blue color indicates the opposite direction of effect for PR interval and AF risk. The different color scheme shows different odds ratio of AF risk. The nearest gene names are labelled if variants were genome-wide significantly associated with both traits.

Supplementary Figure 12 Bubble plot of phenome-wide association analysis of multi-ancestry PR interval polygenic risk score.

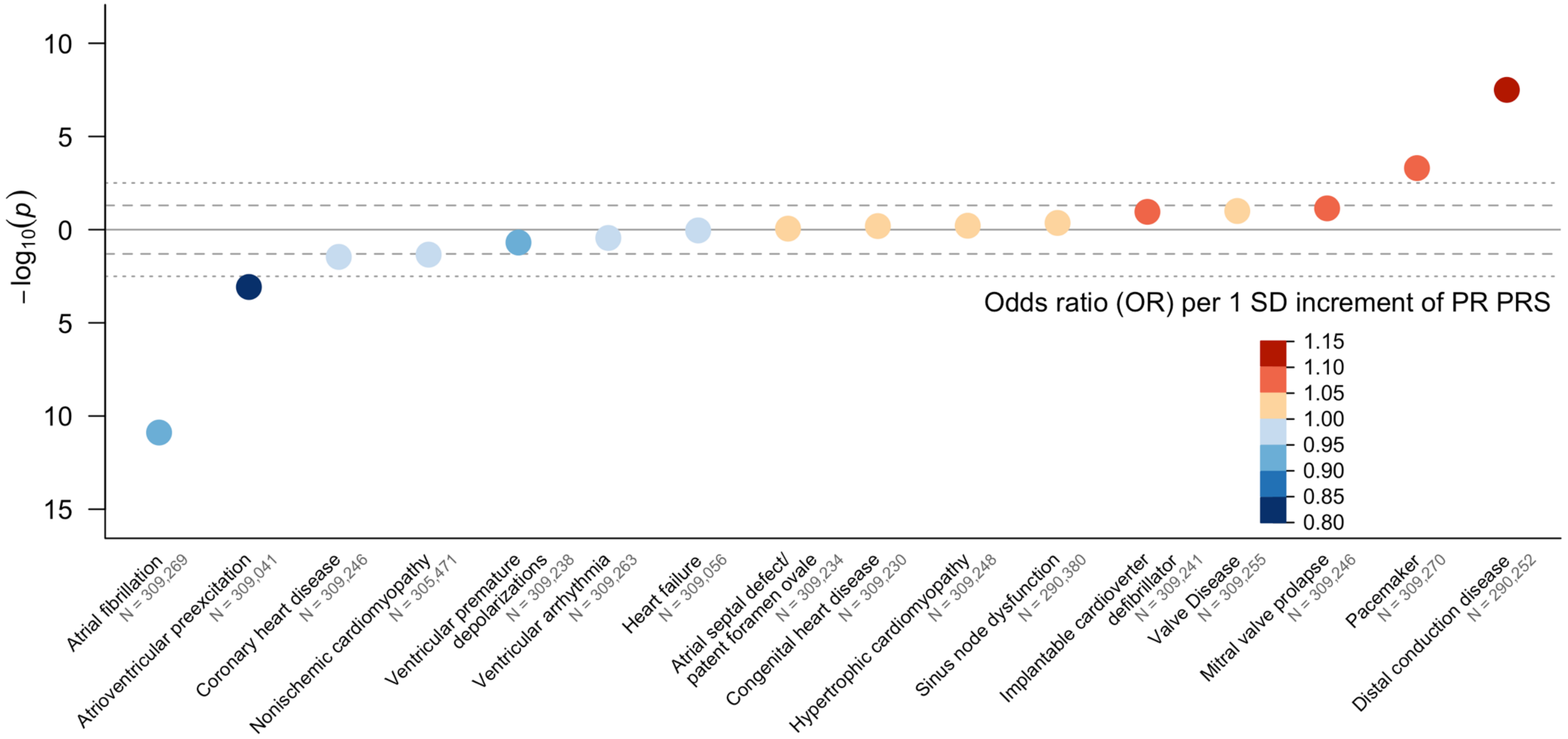

The polygenic risk score was derived from the multi-ancestry meta-analysis. Orange circles indicate that polygenic predisposition to longer PR intervals is associated with an increased risk of the condition, whereas blue circles indicate that polygenic predisposition to longer PR intervals is associated with lower risk of the condition. The darkness of the color reflects the effect size (odds ratio, OR) per 1 standard deviation increment of the polygenic risk score from logistic regression. Sample size (N) in each regression model is provided under X-axis. Given correlation between traits, we set significance threshold at $P<3.13 \times 10^{-3}$ after Bonferroni correction ($P<0.05/16$; dotted line) for the number of analyses performed and also report nominal associations ($P < 0.05$; dashed line).

4. Supplementary Notes

Supplementary Note 1: Acknowledgements

AMISH

We gratefully thank our Amish community and research volunteers for their long-standing partnership in research, and acknowledge the dedication of our Amish liaisons, field workers and the Amish Research Clinic staff, without which these studies would not have been possible.

ARIC

The authors thank the staff and participants of the ARIC study for their important contributions.

BioME

The Mount Sinai BioMe Biobank is supported by The Andrea and Charles Bronfman Philanthropies.

BRIGHT

The BRIGHT study is extremely grateful to all the patients who participated in the study and the BRIGHT nursing team and staff at the Genome Centre for genotyping. This work forms part of the research program of the National Institutes of Health Research (NIHR Cardiovascular Biomedical Research) Cardiovascular Biomedical Centre at Barts and The London, QMUL.

CHRIS

Full acknowledgements for the CHRIS study are reported here: http://translational-medicine.biomedcentral.com/articles/10.1186/s12967-015-0704-9#Declarations. The CHRIS study was funded by the Department of Innovation, Research, and University of the Autonomous Province of Bolzano-South Tyrol.

CROATIA-Korcula

We would like to acknowledge the contributions of the recruitment team in Korcula, the administrative teams in Croatia and Edinburgh and the people of Korcula. The SNP genotyping for the KORCULA cohort was performed in Helmholtz Zentrum München, Neuherberg, Germany and the Clinical Research Facility Genetics Core, University of Edinburgh at the Western General Hospital, Edinburgh, UK.

CROATIA-Split

We would like to acknowledge the contributions of the recruitment team from the Croatian Centre for Global Health, University of Split, the administrative teams in Croatia and Edinburgh and the people of Split. The SNP genotyping for the CROATIA_Split cohort was performed by AROS Applied Biotechnology, Aarhus, Denmark and the Clinical Research Facility Genetics Core, University of Edinburgh at the Western General Hospital, Edinburgh, UK.

deCODE

The deCODE researchers gratefully thank the study participants, the staff at deCODE and our recruitment center, and all our collaborators for invaluable contributions to the understanding of the genetics of health and disease.

ERF

We are grateful to all study participants and their relatives, general practitioners and neurologists for their contributions to the ERF study and to P Veraart for her help in genealogy, J Vergeer for the supervision of the laboratory work and P Snijders for his help in data collection.

FHS

We thank the FHS participants and staff for their contributions to this work.

FINCAVAS

The authors thank the staff of the Department of Clinical Physiology for collecting the exercise test data.

GAPP

We thank the GAPP staff and all GAPP study participants for their important contributions

GRAPHIC

This work falls under the portfolio of research supported by the NIHR Leicester Biomedical Research Centre.

GS:SFHS

We are grateful to all the families who took part, the general practitioners and the Scottish School of Primary Care for their help in recruiting them, and the whole Generation Scotland team, which includes interviewers, computer and laboratory technicians, clerical workers, research scientists, volunteers, managers, receptionists, healthcare assistants and nurses.

HCHS/SOL

We thank the participants and staff of the HCHS/SOL study for their contributions to this study.

Health 2000

The Health 2000 Survey is extremely grateful to all the participants of the study.

INGI-CARL/ INGI-FVG

We are very grateful to the municipal administrators for their collaboration on the project and for logistic support. We would like to thank all participants to this study.

JHS

We thank the Jackson Heart Study (JHS) participants and staff for their contributions to this work.

KORA F3/ KORA S4

The KORA study was initiated and financed by the Helmholtz Zentrum München – German Research Center for Environmental Health, which is funded by the German Federal Ministry of Education and Research (BMBF) and by the State of Bavaria. Furthermore, KORA research was supported within the Munich Center of Health Sciences (MC-Health), Ludwig-Maximilians-Universität, as part of LMUinnovativ.

Lifelines

We thank U. Bultmann (1), J.M. Geleijnse (2), P. van der Harst (3), S. Mulder (4), J.G.M. Rosmalen (5), E.F.C. van Rossum (6), H.A. Smit (7), M. Swertz (8), E.A.L.M. Verhagen (9)

(1) Department of Social Medicine, University Medical Center Groningen, University of Groningen, The Netherlands, (2) Department of Human Nutrition, Wageningen University, The Netherlands, (3) Department of Cardiology, University Medical Center Groningen, University of Groningen, The Netherlands, (4) Lifelines Cohort Study, The Netherlands, (5) Interdisciplinary Center of Psychopathology of Emotion Regulation (ICPE), Department of

Psychiatry, University Medical Center Groningen, University of Groningen, The Netherlands, (6) Department of Endocrinology, Erasmus Medical Center, Rotterdam, The Netherlands, (7) Department of Public Health, University Medical Center Utrecht, The Netherlands, (8) Department of Genetics, University Medical Center Groningen, University of Groningen, The Netherlands, (9) Department of Public and Occupational Health, VU Medical Center, Amsterdam, The Netherlands

MICROS

We owe a debt of gratitude to all participants, all primary care practitioners, and the personnel of the Hospital of Silandro (Department of Laboratory Medicine) for their participation and collaboration in the research project. The study was supported by the Ministry of Health and Department of Educational Assistance, University and Research of the Autonomous Province of Bolzano, the South Tyrolean Sparkasse Foundation, and the European Union framework program 6 EUROSPAN project (contract no. LSHG-CT-2006-018947).

NEO

The authors of the NEO study thank all individuals who participated in the Netherlands Epidemiology in Obesity study, all participating general practitioners for inviting eligible participants and all research nurses for collection of the data. We thank the NEO study group, Pat van Beelen, Petra Noordijk and Ingeborg de Jonge for the coordination, lab and data management of the NEO study. We also thank Arie Maan for the analyses of the electrocardiograms.

ORCADES

DNA extractions were performed at the Wellcome Trust Clinical Research Facility in Edinburgh. We would like to acknowledge the invaluable contributions of the research nurses in Orkney, the administrative team in Edinburgh and the people of Orkney.

PIVUS

The investigators express their deepest gratitude to the study participants.

PROSPER

The PROSPER study was supported by an investigator initiated grant obtained from Bristol-Myers Squibb. Prof. Dr. J. W. Jukema is an Established Clinical Investigator of the Netherlands Heart Foundation (grant 2001 D 032). Support for genotyping was provided by the seventh framework program of the European commission (grant 223004) and by the Netherlands Genomics Initiative (Netherlands Consortium for Healthy Aging grant 050-060-810).

RS

We thank Pascal Arp, Mila Jhamai, Dr Michael Moorhouse, Marijn Verkerk, and Sander Bervoets for their help in creating the GWAS database. The authors are very grateful to the participants and staff from the Rotterdam Study, the participating general practitioners and the pharmacists.

SardiNIA

We thank the many volunteers who generously participated in this study, the Mayors and citizens of the Sardinian towns involved, the head of the Public Health Unit ASL4, and the province of Ogliastra for their volunteerism and cooperation. In addition, we are grateful to the Mayor and the administration in Lanusei for providing and furnishing the clinic site. We are grateful to the physicians Angelo Scuteri, Marco Orrù, Maria Grazia Pilia, Liana Ferreli, Francesco Loi, nurses Paola Loi, Monica Lai and Anna Cau who carried out participant physical exams; the recruitment personnel Susanna Murino; and Mariano Dei, Sandra Lai, Andrea Maschio, Fabio Busonero for genotyping. This research was supported by the Intramural Research Program of the NIH, National Institute on Aging with contracts N01-AG-1-2109 and HHSN271201100005C, and by grant FaReBio2011 “Farmaci e Reti Biotecnologiche di Qualità”.

UK Biobank

This research has been conducted using the UK Biobank Resource (application 8256 - Understanding genetic influences in the response of the cardiac electrical system to exercise). The UK Biobank study was approved by the National Health Service National Research Ethics Service (ref.11/NW/0382 and all participants provided written informed consent. This work forms part of the research program of the National Institutes of Health Research (NIHR Cardiovascular Biomedical Research) Cardiovascular Biomedical Centre at Barts and The London, QMUL. PD Lambiase acknowledges support from the UCLH Biomedicine NIHR.

Polygenic risk score analyses were also conducted using the UK Biobank Resource (application 17488 - Understanding the Genetic Basis of Cardiac Arrhythmias).

ULSAM

The investigators express their deepest gratitude to the study participants.

WHI

The WHI program is supported by contracts from the National Heart, Lung and Blood Institute, NIH. The authors thank the WHI investigators and staff for their dedication, and the study participants for making the program possible. All contributors to WHI science are listed at https://www.whi.org/researchers/Documents%20%20Write%20a%20Paper/WHI%20Investigator%20Long%20List.pdf.https://www.whi.org/researchers/Documents%20%20Write%20a%20Paper/WHI%20Investigator%20Short%20List.pdf.

YFS

We thank the teams that collected data at all measurement time points; the persons who participated as both children and adults in these longitudinal studies; and biostatisticians Irina Lisinen, Johanna Ikonen, Noora Kartiosuo, Ville Aalto, and Jarno Kankaanranta for data management and statistical advice.

Additional acknowledgements

This research utilized Queen Mary's Apocrita HPC facility, supported by QMUL Research-IT. http://doi.org/10.5281/zenodo.438045

Figure 5 was designed using BioRender software (biorender.io).

Supplementary Note 2: Funding

AMISH

The Amish studies are supported by grants and contracts from the NIH, including R01 AG18728, R01 HL088119, U01 GM074518, U01 HL072515, U01 HL84756, U01 HL137181, R01 DK54261,the University of Maryland General Clinical Research Center, grant M01 RR 16500, and the Mid-Atlantic Nutrition Obesity Research Center grant P30 DK72488, the Baltimore Diabetes Research and Training Center grant P60DK79637.

ARIC

The Atherosclerosis Risk in Communities Study is carried out as a collaborative study supported by National Heart, Lung, and Blood Institute contracts (HHSN268201100005C, HHSN268201100006C, HHSN268201100007C, HHSN268201100008C, HHSN268201100009C, HHSN268201100010C, HHSN268201100011C, and HHSN268201100012C), R01HL087641, R01HL59367 and R01HL086694; National Human Genome Research Institute contract U01HG004402; and National Institutes of Health contract HHSN268200625226C. Infrastructure was partly supported by Grant Number UL1RR025005, a component of the National Institutes of Health and NIH Roadmap for Medical Research.

BAMBUÍ

Ministry of Science and Technology (Financiadora de Estudos e Projetos – FINEP and Conselho Nacional de Desenvolvimento Científico e Tecnologico – CNPq), and Fundação de Amparo a Pesquisa do Estado de Minas Gerais (FAPEMIG).

BRIGHT

This work was supported by the Medical Research Council of Great Britain (grant number G9521010D) and the British Heart Foundation (grant number PG/02/128).

Broad-AF

Dr. Lubitz is supported by NIH grant 1R01HL139731 and American Heart Association 18SFRN34250007. Dr. Fatkin is supported by the Victor Chang Cardiac Research Institute, National Health and Medical Research Council of Australia, Estate of the Late RT Hall, St Vincent's Clinic Foundation, and the Simon Lee Foundation. Dr. Olesen is supported by the Hallas-Møller Emerging Investigator Novo Nordisk (NNF17OC0031204). Dr. Smith was supported by grants from the Swedish Heart-Lung Foundation (2016-0134 and 2016-0315), the Swedish Research Council (2017-02554), the European Research Council (ERC-STG-2015-679242), the Crafoord Foundation, Skåne University Hospital, the Scania county, governmental funding of clinical research within the Swedish National Health Service, a generous donation from the Knut and Alice Wallenberg foundation to the Wallenberg Center for Molecular Medicine in Lund, and funding from the Swedish Research Council (Linnaeus grant Dnr 349-2006-237, Strategic Research Area Exodiab Dnr 2009-1039) and Swedish Foundation for Strategic Research (Dnr IRC15-0067) to the Lund University Diabetes Center. Dr. Weng is supported by the American Heart Association Postdoctoral Fellowship Award 17POST33660226. This work was also supported by an American Heart Association Strategically Focused Research Networks (SFRN) postdoctoral fellowship to Drs. Weng and Hall (18SFRN34110082).

This work was supported by grants from the National Institutes of Health to Dr. Ellinor (1RO1HL092577, R01HL128914, K24HL105780). Dr. Ellinor is also supported by the American Heart Association (18SFRN34110082) and by the Fondation Leducq (14CVD01). This research was also supported by the Carol and Roch Hillenbrand and the George L. Nardi, MD, funds at Massachusetts General Hospital. The GGAF is supported by funding to the 5 sources that form GGAF. The AF RISK study is supported by the Netherlands Heart Foundation (grant NHS2010B233), and the Center for Translational Molecular Medicine. Both the Young-AF and Biomarker-AF studies are supported by funding from the University Medical Center Groningen. The GIPS-III trial was supported by grant 95103007 from ZonMw, the Netherlands Organization for Health Research and Development. The PREVEND study is supported by the Dutch Kidney Foundation (grant E0.13) and the Netherlands Heart Foundation (grant NHS2010B280). Dr. Rienstra acknowledges support from the Netherlands Cardiovascular Research Initiative: an initiative supported by the Netherlands Heart Foundation, CVON 2014–9: “Reappraisal of Atrial Fibrillation: interaction between hyperCoagulability, Electrical remodelling, and Vascular destabilization in the progression of AF (RACE V). The Intermountain was supported by funding from the Dell Loy Hansen Heart Foundation. The re-examination of the MPP study was supported by The Swedish Heart-Lung Foundation, Merck, Sharp & Dohme, Hulda and E Conrad Mossfelts Foundation and Ernhold Lundströms Foundation. J. Gustav Smith was supported by the European Research Council, Swedish Heart-Lung Foundation, the Wallenberg Center for Molecular Medicine at Lund University, the Swedish Research Council, the Crafoord Foundation, governmental funding of clinical research within the Swedish National Health Service, Skåne University Hospital in Lund, and the Scania county. Vanderbilt University Medical Center’s BioVU projects are supported by numerous sources: institutional funding, private agencies, and federal grants. These include the NIH funded Shared Instrumentation Grant S10RR025141; CTSA grants

UL1TR002243, UL1TR000445, and UL1RR024975. Genomic data are also supported by investigator-led projects that include U01HG004798, R01NS032830, RC2GM092618, P50GM115305, U01HG006378, U19HL065962, R01HD074711; and additional funding sources listed at https://victr.vanderbilt.edu/pub/biovu/?sid=229. The Danish AF Study support from the John and Birthe Meyer Foundation, The Research Foundation at Rigshospitalet, Villadsen Family Foundation, The Arvid Nilsson Foundation, and The Hallas-Møller Emerging Investigator Novo Nordisk (NNF17OC0031204).

CHS

Cardiovascular Health Study: This CHS research was supported by NHLBI contracts HHSN268201200036C, HHSN268200800007C, HHSN268201800001C, N01HC55222, N01HC85079, N01HC85080, N01HC85081, N01HC85082, N01HC85083, N01HC85086, and HHSN268200960009C; and NHLBI grants U01HL080295, R01HL087652, R01HL105756, R01HL103612, R01HL120393, R01HL085251, and U01HL130114 with additional contribution from the National Institute of Neurological Disorders and Stroke (NINDS). Additional support was provided through R01AG023629 from the National Institute on Aging (NIA). A full list of principal CHS investigators and institutions can be found at CHS-NHLBI.org.

The provision of genotyping data was supported in part by the National Center for Advancing Translational Sciences, CTSI grant UL1TR001881, and the National Institute of Diabetes and Digestive and Kidney Disease Diabetes Research Center (DRC) grant DK063491 to the Southern California Diabetes Endocrinology Research Center.

The content is solely the responsibility of the authors and does not necessarily represent the official views of the National Institutes of Health.

CROATIA-Korcula/CROATIA-Split

Medical Research Council UK and the Ministry of Science, Education and Sport in the Republic of Croatia (number 108-1080315-0302).

deCODE

All authors affiliated with deCODE genetics/Amgen, Inc. are employed by the company

ERF

The ERF study as a part of EUROSPAN (European Special Populations Research Network) was supported by European Commission FP6 STRP grant number 018947 (LSHG-CT-2006-01947) and also received funding from the European Community's Seventh Framework Programme (FP7/2007-2013)/grant agreement HEALTH-F4-2007-201413 by the European Commission under the programme "Quality of Life and Management of the Living Resources" of 5th Framework Programme (no. QLG2-CT-2002-01254). The ERF study was further supported by ENGAGE consortium and CMSB. High-throughput analysis of the ERF data was supported by joint grant from Netherlands Organization for Scientific Research and the Russian Foundation for Basic Research (NWO-RFBR 047.017.043).

FHS

The Framingham Heart Study is funded by National Institutes of Health contract N01-HC-25195; HHSN268201500001I. The current project was also supported by 1R01HL128914; 2R01 HL092577; 1P50HL120163 and 18SFRN34110082.

FINCAVAS

The Finnish Cardiovascular Study (FINCAVAS) has been financially supported by the Competitive Research Funding of the Tampere University Hospital (Grant 9M048 and 9N035), the Finnish Cultural Foundation, the Finnish Foundation for Cardiovascular Research, the Emil Aaltonen Foundation, Finland, the Tampere Tuberculosis Foundation, and EU Horizon 2020 (grant 755320 for TAXINOMISIS).

GAPP

The GAPP study was supported by the Liechtenstein Government, the Swiss Heart Foundation, the Swiss Society of Hypertension, the University of Basel, the University Hospital Basel, the Hanela Foundation, the Mach-Gaensslen Foundation, Schiller AG, and Novartis.

GRAPHIC

The GRAPHIC Study was funded by the British Heart Foundation (BHF/RG/2000004). CPN and NJS are supported by the British Heart Foundation and NJS is a NIHR Senior Investigator

GS:SFHS

Generation Scotland received core support from the Chief Scientist Office of the Scottish Government Health Directorates [CZD/16/6] and the Scottish Funding Council [HR03006]. Genotyping of the GS:SFHS samples was carried out by the Genetics Core Laboratory at the Wellcome Trust Clinical Research Facility, Edinburgh, Scotland and was funded by the Medical Research Council UK and the Wellcome Trust (Wellcome Trust Strategic Award "STratifying Resilience and Depression Longitudinally" (STRADL) Reference 104036/Z/14/Z).

HCHS/SOL

The baseline examination of HCHS/SOL was carried out as a collaborative study supported by contracts from the National Heart, Lung, and Blood Institute (NHLBI) to the University of North Carolina (N01-HC65233), University of Miami (N01-HC65234), Albert Einstein College of Medicine (N01-HC65235), Northwestern University (N01-HC65236) and San Diego State University (N01-HC65237). The following Institutes/Centers/Offices contributed to the first phase of HCHS/SOL through a transfer of funds to the NHLBI: National Institute on Minority Health and Health Disparities, National Institute on Deafness and Other Communication Disorders, National Institute of Dental and Craniofacial Research (NIDCR), National Institute of Diabetes and Digestive and Kidney Diseases, National Institute of Neurological Disorders and Stroke, NIH Institution-Office of Dietary Supplements. The Genetic Analysis Center at University of Washington was supported by NHLBI and NIDCR contracts (HHSN268201300005C AM03 and MOD03). Genotyping efforts were supported by NHLBI HSN 26220/20054C, NCATS CTSI grant UL1TR000124, and NIDDK Diabetes Research Center (DRC) grant DK063491.

Health 2000

The Health 2000 Survey was funded by the National Institute for Health and Welfare (THL), the Finnish Centre for Pensions (ETK), the Social Insurance Institution of Finland (KELA), the Local Government Pensions Institution (KEVA) and other organizations listed on the website of the survey (https://thl.fi/fi/tutkimus-ja-kehittaminen/tutkimukset-ja-hankkeet/terveys-2000-2011/yhteistyokumppanit).

INGI-CARL/INGI-FVG

MH 5X1000 funding to IRCCS Burlo Garofolo

JHS

The JHS is supported by contracts HHSN268201300046C, HHSN268201300047C, HHSN268201300048C, HHSN268201300049C, HHSN268201300050C from the National Heart, Lung, and Blood Institute and the National Institute on Minority Health and Health Disparities. Dr. Wilson is supported by U54GM115428 from the National Institute of General Medical Sciences.

Lifelines

The LifeLines Cohort Study, and generation and management of GWAS genotype data for the LifeLines Cohort Study is supported by the Netherlands Organization of Scientific Research NWO (grant 175.010.2007.006), the Economic Structure Enhancing Fund (FES) of the Dutch

government, the Ministry of Economic Affairs, the Ministry of Education, Culture and Science, the Ministry for Health, Welfare and Sports, the Northern Netherlands Collaboration of Provinces (SNN), the Province of Groningen, University Medical Center Groningen, the University of Groningen, Dutch Kidney Foundation and Dutch Diabetes Research Foundation.

MESA

MESA and the MESA SHARe project are conducted and supported by the National Heart, Lung, and Blood Institute (NHLBI) in collaboration with MESA investigators. Support for MESA is provided by contracts HHSN268201500003I, N01-HC-95159, N01-HC-95160, N01-HC-95161, N01-HC-95162, N01-HC-95163, N01-HC-95164, N01-HC-95165, N01-HC-95166, N01-HC-95167, N01-HC-95168, N01-HC-95169, UL1-TR-000040, UL1-TR-001079, UL1-TR-001420, UL1-TR-001881, and DK063491. Funding for SHARe genotyping was provided by NHLBI Contract N02-HL-64278. Genotyping was performed at Affymetrix (Santa Clara, California, USA) and the Broad Institute of Harvard and MIT (Boston, Massachusetts, USA) using the Affymetrix Genome-Wide Human SNP Array 6.0.

NEO

The genotyping in the NEO study was supported by the Centre National de Génotypage (Paris, France), headed by Jean-Francois Deleuze. The NEO study is supported by the participating Departments, the Division and the Board of Directors of the Leiden University Medical Center, and by the Leiden University, Research Profile Area Vascular and Regenerative Medicine. Dennis Mook-Kanamori is supported by Dutch Science Organization (ZonMW-VENI Grant 916.14.023).

ORCADES

The Orkney Complex Disease Study (ORCADES) was supported by the Chief Scientist Office of the Scottish Government (CZB/4/276, CZB/4/710), a Royal Society URF to J.F.W., the MRC Human Genetics Unit quinquennial programme “QTL in Health and Disease”, Arthritis Research UK and the European Union framework program 6 EUROSPAN project (contract no. LSHG-CT-2006-018947).

PIVUS

PIVUS was supported by Knut and Alice Wallenberg Foundation (Wallenberg Academy Fellow), European Research Council (ERC Starting Grant), Swedish Diabetes Foundation (2013-024), Swedish Research Council (2012-1397, 2012-1727, and 2012-2215), Marianne and Marcus Wallenberg Foundation, County Council of Dalarna, Dalarna University, and Swedish Heart-Lung Foundation (20120197). Genotyping was funded by the Wellcome Trust under awards WT064890 and WT086596. Analysis of genetic data was funded by the Wellcome Trust under awards WT098017 and WT090532.

PREVEND

PREVEND genetics is supported by the Dutch Kidney Foundation (Grant E033), the EU project grant GENECURE (FP-6 LSHM CT 2006 037697), the National Institutes of Health (grant 2R01LM010098), The Netherlands organization for health research and development (NWO-Groot grant 175.010.2007.006, NWO VENI grant 916.761.70, ZonMw grant 90.700.441), and the Dutch Inter University Cardiology Institute Netherlands (ICIN). N

Verweij was supported by NWO VENI (016.186.125). We would like to thank the Center for Information Technology of the University of Groningen for their support and for providing access to the Peregrine high-performance computing cluster.

PROSPER

The PROSPER study was supported by an investigator initiated grant obtained from Bristol-Myers Squibb. Prof. Dr. J. W. Jukema is an Established Clinical Investigator of the Netherlands Heart Foundation (grant 2001 D 032). Support for genotyping was provided by the seventh framework program of the European commission (grant 223004) and by the Netherlands Genomics Initiative (Netherlands Consortium for Healthy Aging grant 050-060-810).

RS

The Rotterdam Study is supported by the Erasmus Medical Center and Erasmus University Rotterdam; the Netherlands Organization for Scientific Research (NWO); the Netherlands Organization for Health Research and Development (ZonMw); the Research Institute for Diseases in the Elderly (RIDE); the Netherlands Heart Foundation; the Ministry of Education, Culture and Science; the Ministry of Health Welfare and Sports; the European Commission; and the Municipality of Rotterdam. Support for genotyping was provided by the Netherlands Organization of Scientific Research NWO Investments (nr. 175.010.2005.011, 911-03-012), the Research Institute for Diseases in the Elderly (014-93-015; RIDE2), the Netherlands Genomics Initiative (NGI)/Netherlands Consortium for Healthy Aging (NCHA) project nr. 050- 060-810. Jacqueline Witteman is supported by NWO grant (vici, 918-76-619). Abbas Dehghan is supported by NWO grant (veni, 916.12.154) and the EUR Fellowship. O.H. Franco

works in ErasmusAGE, a center for aging research across the life course funded by Nestlé Nutrition (Nestec Ltd.) and Metagenics Inc. Nestlé Nutrition (Nestec Ltd.) and Metagenics Inc. had no role in design and conduct of the study; collection, management, analysis, and interpretation of the data; and preparation, review or approval of the manuscript. The GWA study was funded by the Netherlands Organization of Scientific Research NWO Investments (nr. 175.010.2005.011, 911-03-012), the Research Institute for Diseases in the Elderly (014-93-015; RIDE2), the Netherlands Genomics Initiative (NGI)/Netherlands Consortium for Healthy Aging (NCHA) project nr. 050-060-810.

SardiNIA

This study was funded in part by the National Institutes of Health (National Institute on Aging, National Heart Lung and Blood Institute, and National Human Genome Research Institute). This research was supported by the Intramural Research Program of the NIH, National Institute on Aging, with contracts N01-AG-1-2109 and HHSN271201100005C; by National Human Genome Research Institute grants HG005581, HG005552, HG006513, HG007089, HG007022, and HG007089; by National Heart Lung and Blood Institute grant HL117626; and by Sardinian Autonomous Region (L.R. no. 7/2009) grant cRP3-154.

SHIP/SHIP-Trend

SHIP is part of the Community Medicine Research net of the University of Greifswald, Germany, which is funded by the Federal Ministry of Education and Research (grants no. 01ZZ9603, 01ZZ0103, and 01ZZ0403), the Ministry of Cultural Affairs as well as the Social Ministry of the Federal State of Mecklenburg-West Pomerania, and the network 'Greifswald

Approach to Individualized Medicine (GANI_MED)' funded by the Federal Ministry of Education and Research (grant 03IS2061A). Genome-wide data have been supported by the Federal Ministry of Education and Research (grant no. 03ZIK012) and a joint grant from Siemens Healthcare, Erlangen, Germany and the Federal State of Mecklenburg- West Pomerania.

TwinsUK

TwinsUK is funded by the Wellcome Trust, Medical Research Council, European Union, the National Institute for Health Research (NIHR)-funded BioResource, Clinical Research Facility and Biomedical Research Centre based at Guy's and St Thomas'NHS Foundation Trust in partnership with King's College London. This work was supported by the British Heart Foundation (grant number PG/12/38/29615).

UK Biobank

This work is supported by Medical Research Council grant MR/N025083/1. MO is supported by an IEF 2013 Marie Curie fellowship. JR acknowledges support from the European Union's Horizon 2020 research and innovation programme under the Marie-Sklodowska-Curie grant agreement No. 786833.

ULSAM

ULSAM was supported by Knut and Alice Wallenberg Foundation (Wallenberg Academy Fellow), European Research Council (ERC Starting Grant), Swedish Diabetes Foundation (2013-024), Swedish Research Council (2012-1397, 2012-1727, and 2012-2215), Marianne

and Marcus Wallenberg Foundation, County Council of Dalarna, Dalarna University, and Swedish Heart-Lung Foundation (20120197). Genotyping was funded by the Wellcome Trust under awards WT064890 and WT086596. Analysis of genetic data was funded by the Wellcome Trust under awards WT098017 and WT090532.

WHI

The WHI program is funded by the National Heart, Lung, and Blood Institute, National Institutes of Health, U.S. Department of Health and Human Services through contracts HHSN268201600018C, HHSN268201600001C, HHSN268201600002C, HHSN268201600003C, and HHSN268201600004C. Within the WHI, GARNET was funded by the National Human Genome Research Institute through U01-HG005152 (Reiner). GECCO was funded by the National Cancer Institute through R01-CA059045 and U01-CA137088 (Peters). HIPFX was funded by the NHLBI through N01-WH74316 (Jackson). MOPMAP was funded by the National Institute of Environmental Health Sciences through R01-ES017794 (Whitsel) and the University of North Carolina Cancer Research Fund. WHIMS was funded by the NHLBI and NIA through N01-WH44221, 271201100004C and 263200700042C (Shumaker).

YFS

The Young Finns Study has been financially supported by the Academy of Finland: grants 286284, 134309 (Eye), 126925, 121584, 124282, 129378 (Salve), 117787 (Gendi), and 41071 (Skidi); the Social Insurance Institution of Finland; Competitive State Research Financing of the Expert Responsibility area of Kuopio, Tampere and Turku University Hospitals (grant

X51001); Juho Vainio Foundation; Paavo Nurmi Foundation; Finnish Foundation for Cardiovascular Research ; Finnish Cultural Foundation; The Sigrid Juselius Foundation; Tampere Tuberculosis Foundation; Emil Aaltonen Foundation; Yrjö Jahnsson Foundation; Signe and Ane Gyllenberg Foundation; Diabetes Research Foundation of Finnish Diabetes Association; and EU Horizon 2020 (grant 755320 for TAXINOMISIS); European Research Council (grant 742927 for MULTIEPIGEN project); and Tampere University Hospital Supporting Foundation.

5. Supplementary References

1. The Atherosclerosis Risk in Communities (ARIC) Study: design and objectives. The ARIC investigators. *Am J Epidemiol* **129**, 687-702 (1989).
2. Roselli, C. *et al.* Multi-ethnic genome-wide association study for atrial fibrillation. *Nat Genet* **50**, 1225-1233 (2018).
3. Conen, D. *et al.* Genetic and phenotypic determinants of blood pressure and other cardiovascular risk factors (GAPP). *Swiss Med Wkly* **143**, w13728 (2013).
4. Smith, B.H. *et al.* Cohort Profile: Generation Scotland: Scottish Family Health Study (GS:SFHS). The study, its participants and their potential for genetic research on health and illness. *Int J Epidemiol* **42**, 689-700 (2013).
5. McQuillan, R. *et al.* Runs of homozygosity in European populations. *Am J Hum Genet* **83**, 359-72 (2008).
6. Hofman, A. *et al.* The Rotterdam Study: 2016 objectives and design update. *Eur J Epidemiol* **30**, 661-708 (2015).
7. Bycroft, C. *et al.* The UK Biobank resource with deep phenotyping and genomic data. *Nature* **562**, 203-209 (2018).
8. Orini, M., Tinker, A., Munroe, P.B. & Lambiase, P.D. Long-term intra-individual reproducibility of heart rate dynamics during exercise and recovery in the UK Biobank cohort. *PLoS One* **12**, e0183732 (2017).
9. Ramirez, J. *et al.* Thirty loci identified for heart rate response to exercise and recovery implicate autonomic nervous system. *Nat Commun* **9**, 1947 (2018).
10. van Setten, J. *et al.* PR interval genome-wide association meta-analysis identifies 50 loci associated with atrial and atrioventricular electrical activity. *Nat Commun* **9**, 2904 (2018).
11. Verweij, N. *et al.* Genetic determinants of P wave duration and PR segment. *Circ Cardiovasc Genet* **7**, 475-81 (2014).
12. van Setten, J. *et al.* Genome-wide association meta-analysis of 30,000 samples identifies seven novel loci for quantitative ECG traits. *Eur J Hum Genet* **27**, 952-962 (2019).
13. Women's Health Initiative Study Group. WHI Extension Study Protocol. 2012. https://www.whi.org/researchers/studydoc/Consents/Protocol%202010-2015.pdf. (2012).
14. National Institutes of Environmental Health Sciences. Modification of PM-mediated Arrhythmogenesis in Populations. http://projectreporter.nih.gov/project_info_description.cfm?aid=7984809&icde=19283008. (2012).
15. Shumaker, S.A. *et al.* The Women's Health Initiative Memory Study (WHIMS): a trial of the effect of estrogen therapy in preventing and slowing the progression of dementia. *Control Clin Trials* **19**, 604-21 (1998).
16. Butler, A.M. *et al.* Novel loci associated with PR interval in a genome-wide association study of 10 African American cohorts. *Circ Cardiovasc Genet* **5**, 639-46 (2012).
17. Chambers, J.C. *et al.* Genetic variation in SCN10A influences cardiac conduction. *Nat Genet* **42**, 149-52 (2010).
18. Holm, H. *et al.* Several common variants modulate heart rate, PR interval and QRS duration. *Nat Genet* **42**, 117-22 (2010).
19. Hong, K.W. *et al.* Identification of three novel genetic variations associated with electrocardiographic traits (QRS duration and PR interval) in East Asians. *Hum Mol Genet* **23**, 6659-67 (2014).

20. Pfeufer, A. *et al.* Genome-wide association study of PR interval. *Nat Genet* **42**, 153-9 (2010).
21. Sano, M. *et al.* Genome-wide association study of electrocardiographic parameters identifies a new association for PR interval and confirms previously reported associations. *Hum Mol Genet* **23**, 6668-76 (2014).
22. Genomes Project, C. *et al.* A global reference for human genetic variation. *Nature* **526**, 68-74 (2015).
23. Purcell, S. *et al.* PLINK: a tool set for whole-genome association and population-based linkage analyses. *Am J Hum Genet* **81**, 559-75 (2007).
24. Consortium, E.P. An integrated encyclopedia of DNA elements in the human genome. *Nature* **489**, 57-74 (2012).
25. Bernstein, B.E. *et al.* The NIH Roadmap Epigenomics Mapping Consortium. *Nat Biotechnol* **28**, 1045-8 (2010).
26. Staley, J.R. *et al.* PhenoScanner: a database of human genotype-phenotype associations. *Bioinformatics* **32**, 3207-3209 (2016).
27. Nielsen, J.B. *et al.* Genome-wide Study of Atrial Fibrillation Identifies Seven Risk Loci and Highlights Biological Pathways and Regulatory Elements Involved in Cardiac Development. *Am J Hum Genet* **102**, 103-115 (2018).
