# Supplementary material for: Multi-ancestry GWAS of the electrocardiographic PR interval identifies 202 loci underlying cardiac conduction
Source: Nat Commun. 2020 May 21;11:2542. doi: 10.1038/s41467-020-15706-x (PMC7242331; doi:10.1038/s41467-020-15706-x)
Supplement: Supplementary file 3 — Description of Additional Supplementary Files [file 41467_2020_15706_MOESM3_ESM.pdf]

Description of Supplementary Data Files

Multi-ancestry GWAS of the electrocardiographic PR interval identifies 202 loci underlying cardiac conduction.

Ntalla et al.

Supplementary Data 1 Description of studies contributing to multi-ancestry meta-analysis: genotyping, quality control, pre-phasing, imputation, and association analyses.

Supplementary Data 2 Description of studies contributing to PR interval meta-analyses: descriptive statistics of PR interval and covariates included in the analyses separately for each ancestry group.

Supplementary Data 3 Previously not reported PR interval loci in the multi-ancestry meta-analysis (N = 141).

Supplementary Data 4 Previously reported genetic loci for PR interval.

Supplementary Data 5 Most significant variants within the 64 previously reported loci for PR interval in the multi-ancestry meta-analysis.

Supplementary Data 6 Genome-wide significant loci (N=127) not previously reported with PR interval identified by the European GWAS meta-analysis.

Supplementary Data 7 Genome-wide significant loci in the African (a) and Hispanic (b) ancestry meta-analyses of absolute PR interval.

Supplementary Data 8 Genome-wide significant loci reporting across the meta-analyses of absolute PR interval and rank-based inverse normal transformed PR interval residuals.

Supplementary Data 9 Genome-wide significant loci outside the 64 previously reported loci in the multi-ancestry meta-analysis of rank-based inverse normal transformed residuals of PR interval.

Supplementary Data 10 Genome-wide significant loci outside the 64 previously reported loci in the European meta-analysis of rank-based inverse normal transformed residuals of PR interval.

Supplementary Data 11 Conditional analysis results for independent variants at previously not reported (a) and previously reported (b) PR interval associated loci.

Supplementary Data 12 Annotation of lead and conditionally independent variants and their proxies (r^2>0.8) at all PR interval associated loci using Variant Effect Predictor.

Supplementary Data 13 Significant cis-eQTLs for PR interval associated variants at previously not reported (a) and previously reported loci (b).

Supplementary Data 14 Significant S-PrediXcan results for associations between gene expressions in heart tissues (left ventricle and right atrial appendage) and PR interval at previously not reported (a) and previously reported loci (b), and ≥ 500kb away from lead variants (c).

Supplementary Data 15 Chromatin states and DNase hypersensitivity sites of lead and conditionally independent variants and their proxies (r^2>0.8) at all PR interval associated loci from Roadmap Epigenomics across all tissues and heart tissues.

Supplementary Data 16 HiC results for all lead and conditionally independent variants or their proxies (r^2>0.8) within previously not reported (a) and previously reported loci (b) for PR interval.

Supplementary Data 17 Previously not reported PR interval loci associations with other traits from PhenoScanner.

Supplementary Data 18 Genes indicated by bioinformatics and in silico functional annotations at the 149 previously not reported loci for PR interval.

Supplementary Data 19 Literature review of nearest genes to the lead variants or biologically plausible genes in the region defined by an r^2>0.5 from the lead variant or genes indicated by bioinformatics analyses**.**

Supplementary Data 20 DEPICT tissue and cell type enrichment results across PR interval associated loci.

Supplementary Data 21 DEPICT geneset enrichment results across PR interval associated loci.

Supplementary Data 22 Results from the Ingenuity Pathway Analysis.

Supplementary Data 23 Disease definitions in UK Biobank.
