# Supplementary material for: Multi-ancestry GWAS of the electrocardiographic PR interval identifies 202 loci underlying cardiac conduction
Source: Nat Commun. 2020 May 21;11:2542. doi: 10.1038/s41467-020-15706-x (PMC7242331; doi:10.1038/s41467-020-15706-x)
Supplement: Supplementary file 27 — Reporting Summary [file 41467_2020_15706_MOESM27_ESM.pdf]

nature research

Corresponding author(s): Patricia B Munroe and Steven A Lubitz

Last updated by author(s): 15/3/20

Reporting Summary

Nature Research wishes to improve the reproducibility of the work that we publish. This form provides structure for consistency and transparency in reporting. For further information on Nature Research policies, see Authors & Referees and the Editorial Policy Checklist .

Statistics

For all statistical analyses, confirm that the following items are present in the figure legend, table legend, main text, or Methods section.

n/a	Confirmed	
☐	☒	The exact sample size (*n*) for each experimental group/condition, given as a discrete number and unit of measurement
☐	☒	A statement on whether measurements were taken from distinct samples or whether the same sample was measured repeatedly
☐	☒	The statistical test(s) used AND whether they are one- or two-sided<br>*Only common tests should be described solely by name; describe more complex techniques in the Methods section.*
☐	☒	A description of all covariates tested
☐	☒	A description of any assumptions or corrections, such as tests of normality and adjustment for multiple comparisons
☐	☒	A full description of the statistical parameters including central tendency (e.g. means) or other basic estimates (e.g. regression coefficient) AND variation (e.g. standard deviation) or associated estimates of uncertainty (e.g. confidence intervals)
☐	☒	For null hypothesis testing, the test statistic (e.g. *F*, *t*, *r*) with confidence intervals, effect sizes, degrees of freedom and *P* value noted<br>*Give P values as exact values whenever suitable.*
☒	☐	For Bayesian analysis, information on the choice of priors and Markov chain Monte Carlo settings
☐	☒	For hierarchical and complex designs, identification of the appropriate level for tests and full reporting of outcomes
☐	☒	Estimates of effect sizes (e.g. Cohen's *d*, Pearson's *r*), indicating how they were calculated

Our web collection on statistics for biologists contains articles on many of the points above.

Software and code

Policy information about availability of computer code

Data collection	N/A

Data analysis

GTEx v7; https://gtexportal.org/home
DEPICT; https://data.broadinstitute.org/mpg/depict.
IPA; https://www.qiagenbioinformatics.com/products/ingenuity-pathway-analysis
EasyQC version 11.4; https://www.uni-regensburg.de/medizin/epidemiologie-praeventivmedizin/genetische-epidemiologie/software/#
MACH, http://csg.sph.umich.edu/abecasis/mach/tour/imputation.html
IMPUTE2; http://mathgen.stats.ox.ac.uk/impute/impute_v2.html
METAL; http://csg.sph.umich.edu/abecasis/metal/, release on 2011/03/25
LocusZoom; http://locuszoom.org/
GCTA v1.91.3; https://cnsgenomics.com/software/gcta/#Overview
BOLT-REML v2.3.4; (https://data.broadinstitute.org/alkesgroup/BOLT-LMM/
HaploReg v4; https://pubs.broadinstitute.org/mammals/haploreg/haploreg.php
FORGE; https://github.com/iandunham/Forge
VEP; https://www.ensembl.org/info/docs/tools/vep/index.html
S-PrediXcan; https://github.com/hakyimlab/MetaXcan
COLOC package (R version 3.5); https://cran.r-project.org/web/packages/coloc/index.html
GARFIELD;https://www.ebi.ac.uk/birney-srv/GARFIELD/
Regulome DB; http://www.regulomedb.org/
PLINK v1.90; https://www.cog-genomics.org/plink/
R v3.5.2; https://cran.r-project.org/

For manuscripts utilizing custom algorithms or software that are central to the research but not yet described in published literature, software must be made available to editors/reviewers. We strongly encourage code deposition in a community repository (e.g. GitHub). See the Nature Research guidelines for submitting code & software for further information.

Data

Policy information about availability of data

All manuscripts must include a data availability statement. This statement should provide the following information, where applicable:
- Accession codes, unique identifiers, or web links for publicly available datasets
- A list of figures that have associated raw data
- A description of any restrictions on data availability

"Summary GWAS statistics are publicly available on the Cardiovascular Disease Knowledge portal (http://www.broadcvdi.org). All other data are contained in the article file and its supplementary information or are available upon request"

Field-specific reporting

Please select the one below that is the best fit for your research. If you are not sure, read the appropriate sections before making your selection.

☒ Life sciences ☐ Behavioural & social sciences ☐ Ecological, evolutionary & environmental sciences

For a reference copy of the document with all sections, see nature.com/documents/nr-reporting-summary-flat.pdf

Life sciences study design

All studies must disclose on these points even when the disclosure is negative.

Sample size

The aim of our study was to discover new loci for the ECG PR interval using the largest sample size possible, thus power calculations were not performed.

Data exclusions

An analysis plan was prepared for the project and this was shared with all studies.

We agreed the phenotypic exclusion criteria in advance for this study and this is copied here: We requested exclusion of individuals with extreme PR interval values (<80ms or >320ms), second/third degree heart block, AF on the ECG, or a history of myocardial infarction or heart failure, Wolff-Parkinson-White syndrome, those who had a pacemaker, individuals receiving class I and class III antiarrhythmic medications, digoxin, and pregnancy. Where data was available these exclusions were applied.

Each contributing study performed the association analyses, centrally we performed genetic QC. The full details are provided in the methods section of the paper, and copied below: We removed variants that were monomorphic, had a minor allele count (MAC) <6, imputation quality metric <0.3 (imputed by MACH; http://csg.sph.umich.edu/abecasis/mach/tour/imputation.html) or 0.4 (imputed by IMPUTE2; http://mathgen.stats.ox.ac.uk/impute/impute_v2.html), had invalid or mismatched alleles, were duplicated, or if they were allele frequency outliers (difference >0.2 from the allele frequency in 1000G project). We inspected PZ plots, effect allele frequency plots, effect size distributions, QQ plots, and compared effect sizes in each study to effect sizes from prior reports for established PR interval loci to identify genotype and study level anomalies. Variants with effective MAC (=2×N×MAF×imputation quality metric) <10 were omitted from each study prior to meta-analysis.

Replication	We did not perform replication analyses as we instead sought to maximize discovery in a large sample with available genome-wide genotyping data. Therefore we imposed conservative thresholds for discovery, and considered only variants present in >60% of the maximum sample size for reporting to ensure robustness of associated loci.
Randomization	Randomisation was not necessary as our analysis was on a quantitative trait across all samples with both measures of the PR interval and genetic data.
Blinding	Blinding was not relevant to our study as we performed a meta-analysis of GWAS and required access to all the data.

Reporting for specific materials, systems and methods

We require information from authors about some types of materials, experimental systems and methods used in many studies. Here, indicate whether each material, system or method listed is relevant to your study. If you are not sure if a list item applies to your research, read the appropriate section before selecting a response.

Materials & experimental systems

n/a	Involved in the study	
x		Antibodies
x		Eukaryotic cell lines
x		Palaeontology
x		Animals and other organisms
	x	Human research participants
x		Clinical data

Methods

n/a	Involved in the study	
x		ChIP-seq
x		Flow cytometry
x		MRI-based neuroimaging

Human research participants

Policy information about studies involving human research participants

Population characteristics	Our analyses includes individuals from 40 different studies and included population samples and controls from case-control studies. In supplementary tables we provide information on study-specific design, and descriptive statistics.
Recruitment	Individuals from 40 studies contributed data for this project. Studies were recruited to the project from the CHARGE-EKG working group, an international collaborative group studying ECG traits. To participate studies had to have PR interval measures from the ECG, all covariates for our analysis model and genetic data. We created an analysis plan which we sent to all studies and centrally performed the meta-analysis.
Ethics oversight	All participating institutions and co-ordinating centres approved this project, and informed consent was obtained from all study participants.

Note that full information on the approval of the study protocol must also be provided in the manuscript.
